# Abnormal Liver Function Tests Were Associated With Adverse Clinical Outcomes: An Observational Cohort Study of 2,912 Patients With COVID-19

**DOI:** 10.3389/fmed.2021.639855

**Published:** 2021-06-09

**Authors:** Yong Lv, Xiaodi Zhao, Yan Wang, Jingpu Zhu, Chengfei Ma, Xiaodong Feng, Yao Ma, Yipeng Zheng, Liyu Yang, Guohong Han, Huahong Xie

**Affiliations:** ^1^State Key Laboratory of Cancer Biology, National Clinical Research Center for Digestive Diseases and Xijing Hospital of Digestive Diseases, Fourth Military Medical University, Xi'an, China; ^2^Endoscopy Center, 986 Air Force Hospital, Xi'an, China; ^3^Student Brigade of Basic Medicine School, Fourth Military Medical University, Xi'an, China; ^4^Department of Liver Diseases and Digestive Interventional Radiology, National Clinical Research Center for Digestive Diseases and Xijing Hospital of Digestive Diseases, Fourth Military Medical University, Xi'an, China; ^5^Huoshen Shan Hospital, Wuhan, China

**Keywords:** coronavirus disease-2019, liver function test abnormality, mortality, severe acute respiratory syndrome coronavirus 2, mechanical ventilation

## Abstract

**Background and Aim:** The impact of liver function test (LFTs) abnormality on adverse clinical outcomes in coronavirus disease 2019 (COVID-19) patients remains controversial. The aim of this study was to assess the impact of abnormal LFTs on clinical outcomes in a large cohort of hospitalized patients with COVID-19.

**Methods:** We retrospectively collected data on 2,912 consecutive patients with COVID-19 who were admitted to a makeshift hospital in China between 5 February and 23 March 2020. The association between LFTs abnormalities (baseline and peak values) and clinical outcomes was measured by using Cox regression models.

**Results:** On admission 1,414 patients (48.6%) had abnormal LFTs, with alanine aminotransferase (ALT), aspartate aminotransferase (AST), total bilirubin (TBIL), alkaline phosphatase (ALP), and gamma-glutamyltransferase (GGT) elevation in 662 (22.7%), 221 (7.6%), 52 (1.8%), 135 (4.6%), and 536 (18.5%) patients, respectively, and hypoalbuminemia in 737 (25.3%) patients. During a median 13 (IQR: 8–19) days of hospitalization, 61 patients (2.1%) died, 106 patients (3.6%) admitted to intensive care unit (ICU), and 75 patients (2.6%) required mechanical ventilation. After adjustment for confounders, baseline abnormal LFTs were independently associated with increased risks of mortality (adjusted HR 3.66, 95%CI 1.64–8.19, *p* = 0.002), ICU admission (adjusted HR 3.12 95%CI 1.86–5.23, *p* < 0.001), and mechanical ventilation (adjusted HR 3.00, 95%CI 1.63–5.52, *p* < 0.001), which was homogeneous across the severity of COVID-19 infection. Among the parameters of LTFs, the associations with the outcomes were more pronounced for AST and albumin abnormality. In contrast, ALT elevation was not significantly associated with those outcomes. Similar results were observed for peak values of LFTs during hospitalization.

**Conclusions:** Abnormality of AST, albumin, TBIL, ALP, and GGT but not ALT were independently associated with adverse outcomes.

## Introduction

The current coronavirus disease 2019 (COVID-19) pandemic, caused by severe acute respiratory syndrome coronavirus 2 (SARSCoV-2), has become a serious threat to global public health (1–4). Although initially reported in Wuhan, China, it has rapidly spread around the world (5). Outcomes of COVID-19 range from asymptomatic infection to death (6, 7). Older age; male gender; and comorbid conditions, such as hypertension and diabetes, have been identified as risk factors for severe outcomes (7, 8). While COVID-19 is typically characterized by symptoms of viral pneumonia, SARS-CoV-2 causes a systemic disease, with possible involvement of the heart, liver, pancreas, and kidneys, as well as alterations in circulating lymphocytes and the immune system, because of the ubiquitous distribution of the main viral entry receptor, namely angiotensin converting enzyme 2 (ACE2) (2, 9, 10).

Liver impairment has been reported as a common manifestation, with a derangement of liver function tests (LFTs) ranging from 14 to 75% (11–27). Nevertheless, the clinical relevance of LFTs abnormalities remains controversial, with some studies suggesting its association with the severity of COVID-19 pneumonia and adverse outcomes, while others not. Most of those reports were small-sized and the parameters of LFTs, the diagnostic time point (i.e., on admission or during disease progression) and cut-off values of abnormal LFTs varies among studies (28, 29). Furthermore, composite outcomes combining admission to intensive care unit (ICU), mechanical ventilation, and/or death, are used in a majority of studies, thus it is difficult to determine whether LFTs abnormalities are equally predictive of all the outcomes evaluated. In addition, due to LFTs were categorized in almost all previous studies, the actual relationship between the LFTs and outcomes (liner, dose-response, threshold/saturation effect pattern, or others) remains unknown. It is also yet unclear whether the effect of LFTs on the outcomes equal or differ among patients with different severity of COVID-19 infection.

Thus, the aim of this study was to assess the clinical features and the impact of abnormal LFTs on the outcomes (mortality, ICU admission, and mechanical ventilation) in a large cohort of hospitalized patients with COVID-19.

## Methods

### Study Design and Participants

We retrospectively extracted the data from the electronic charts of consecutive patients with confirmed COVID-19 at Huoshenshan hospital (Wuhan, China) from 5 February to 23 March 2020. The Huoshenshan hospital, a makeshift hospital with 1,000 beds, was opened by the government on 5 February 2020, and assigned to treat exclusively COVID-19 patients. This study was approved by the National Health Commission of China and the institutional review board at Huoshenshan hospital. Written informed consent was waived by the ethics committee of the Huoshenshan hospital for patients with emerging infectious diseases.

Inclusion criteria for the study were (i) hospitalized patients with confirmed COVID-19 infection; (ii) age >18 years old. Patients with no data on LFTs were excluded from the study. COVID-19 was diagnosed by clinical manifestations, chest computed tomography (CT), and confirmed by real-time polymerase chain reaction (RT-PCR) according to World Health Organization (WHO) interim guidance (30), and the New Coronavirus Pneumonia Prevention and Control Program (7th edition) published by the National Health Commission of China (31). The severity of COVID-19 was categorized as mild, severe, or critical (31, 32). Mild type was defined as having slight clinical symptoms without signs of pneumonia or with mild pneumonia (multiple small patchy shadows and interstitial changes, mainly in the outer zone of the lung and under the pleura) by radiography (31, 32). Severe cases were characterized by dyspnoea, respiratory frequency ≥30/min, blood oxygen saturation ≤ 93%, PaO_2_/FiO_2_ ratio <300 mmHg, and/or lung infiltrates >50% within 24–48 h (31, 32). Such patients were considered as critical case if they developed respiratory failure requiring mechanic ventilation, septic shock, and/or multiple organ dysfunction/failure (31, 32).

### Data Collection

Baseline data collected within 24 h after admission include patient demographics, clinical features at inclusion, clinical history, comorbidities, initial blood pressure, and heart rate, laboratory values (peripheral white blood cell, neutrophil, lymphocyte, hemoglobin, platelet count, creatinine, blood urea nitrogen, potassium, sodium, D-dimer, prothrombin time, activated partial thromboplastin time, international normalized ratio, creatine kinase, lactate dehydrogenase, procalcitonin, and c-reactive protein), and radiological reports. Data regarding the specific drug therapy provided during the hospitalization also were collected. Liver function tests [alanine aminotransferase (ALT), aspartate aminotransferase (AST), albumin, total bilirubin, (TBIL), alkaline phosphatase (ALP), and gamma-glutamyltransferase (GGT)] from the time of hospital admission until discharge or death were obtained. The performing of LFT was determined by the attending physicians based on the demand of clinical decision. LFTs were considered as abnormal when at least one among AST, ALT, albumin, TBIL, ALP, and GGT were above the upper limit of normal (ULN) of laboratory reference range standards (i.e., AST >40 U/L, ALT >45 U/L, albumin <35 g/L, TBIL >26 μmol/L, ALP >125 U/L, GGT >60 U/L). All data were reviewed and confirmed by two certified investigators (Yong Lv and Huahong Xie) to ensure accuracy.

### Outcome and Definitions

The primary endpoint was all-cause mortality during hospitalization. Secondary endpoints included ICU admission and use of mechanical ventilation. All clinical outcomes were obtained from clinical charts and assessed on April 15, 2020, when all survived patients were discharged and the Huoshenshan Hospital was shut down. The criteria for discharge are: (i) throat swab specimens collected 24 h apart were negative for tests of SARS-CoV-2; (ii) body temperature was normal for three consecutive days; (iii) symptoms of COVID-19 were resolved; (iv) the radiographic findings of COVID-19 significantly improved (31).

### Statistical Analysis

For all analyses, missing data of the covariates were imputed with multiple imputations methods (detailed in Supplementary Materials and Methods). Data are presented as frequencies (percentage), mean ± standard deviation (SD), or medians with interquartile range (IQR) as appropriate. Comparisons of variables between groups were performed using Student *t*-test, non-parametric Mann-Whitney U-test, chi-squared test, or Fisher's exact test as appropriate. Dynamic changes in liver function were presented using locally weighted scatterplot smoothing (LOESS). The cumulative probability model [an ordinal regression model for continuous outcomes (33)] was used to evaluate the association of baseline characteristics and treatment before peaking of FLTs with the peak levels of LFTs in hospital, where the liver function markers were treated as continuous response variables. The non-linear relationships between liver function markers and the risk of the evaluated outcomes were visualized using restricted cubic splines by entering the liver function markers as a continuous variable into the logistic regression analysis. Cumulative risks of death was assessed with Kaplan-Meier curves and compared using the log-rank test. Cumulative incidences of ICU admission or mechanical ventilation were estimated in a competing risks setting, where the death competed with the event of interest. The contribution of each variable to the risk of developing the endpoint was reported as a hazard ratio (HR) with 95% confidence interval (CI). We assessed the unadjusted and confounder-adjusted effects of LFTs on the evaluated outcomes using Cox regression models. Age, gender, severity of COVID-19 (severe/critical vs. mild), comorbidities (include hypertension, cardiovascular disease, diabetes, chronic pulmonary diseases, cerebrovascular disease, malignancy, and autoimmune disease) and chronic liver diseases (include hepatitis B virus infection, hepatitis C virus infection, and autoimmune liver disease) were considered as potential confounders. We assessed the heterogeneity in the effect of LFTs across the severity of COVID-19 by including a LFTs-by-COVID-19-severity interaction term in the Cox regression models. A significant interaction would indicate that the effect of LFTs was different across the severity of COVID-19. Significance was established at *p* < 0.05. All statistical calculations were performed using R 3.6.1(http://www.R-project.org/) with the add-on packages *Hmisc, rms, riskRegression, pec, prodlim*, and *cmprsk*.

## Results

### Baseline Clinical Features of Patients With COVID-19

During the study period, 2,922 patients with confirmed COVID-19 were admitted to the Huoshenshan hospital, and 10 patients were excluded because of incomplete relevant data. Ultimately, 2,912 patients with COVID-19 were included in the study. In the entire cohort, the mean age was 58.4 ± 14.4 years, and 1,512 (51.9%) were female. On admission, the severity of COVID-19 was mild in 2,160 (74.2%) patients, severe in 714 (24.5%) and critical in 38 (1.3%). Among the 752 serious and critically ill patients, 54 (7.2%) patients had multiple organ dysfunction syndromes. A total of 1236 (42.4%) patients had comorbidities, with hypertension (910 patients [31.2%]) being the most common one, followed by diabetes (392 patients [13.5%]). Sixty-eight patients (2.3%) had chronic liver disease, among which 58 had hepatitis B virus infection, 8 had hepatitis C virus infection, and 2 autoimmune liver disease. No patients had cirrhosis. The most common symptoms of COVID-19 were fever (2,057 patients [70.6%]), followed by cough (2,001 patients [68.7%]), fatigue (1,461 patients [50.2%]), dyspnea (1,394 patients [47.9%]), myalgia (774 patients [26.6%]), anorexia (523 patients [18.0%]), and expectoration (420 patients [14.4%]). Nausea, vomiting, abdominal pain, diarrhea, headache, dizziness disorders of consciousness were rare.

On admission 1,414 patients (48.6%) had abnormal LFTs, with ALT, AST, TBIL, ALP, and GGT above ULN in 662 (22.7%), 221 (7.6%), 52 (1.8%), 135 (4.6%), and 536 (18.5%) patients, respectively, and hypoalbuminemia (<35g/L) in 737 (25.3%) patients. The baseline characteristics of the study population according to normal and abnormal LFTs on admission are summarized in Table 1. Compared with patients with normal LFTs, patients with abnormal LFTs were older, with more severe COVID-19 disease and more likely to have symptoms of fever, cough, expectoration, dyspnea, fatigue, myalgia, anorexia, and nausea. The mean values of white blood cell count, neutrophil count, lymphocyte count, hemoglobin, platelet count, creatinine, D-dime, activated partial thromboplastin time, creatine kinase, lactate dehydrogenase, procalcitonin, and C-reactive protein were also higher in patients with abnormal LFTs. Mover, patients with abnormal LFTs had a higher likelihood of receiving antiviral therapy, antibiotics, immunoglobin, glucocorticoid therapy, high flow nasal cannula, and continuous renal replacement therapy during hospitalization (Table 2).

**Table 1:** Baseline characteristics of patients according to normal vs. abnormal liver function test on admission.

**Variable**	**All (*n* = 2,912)**	**Normal LFTs (*n* = 1,498)**	**Abnormal LFTs (*n* = 1,414)**	***P*-value**
Age (years)	58.4 ± 14.4	56.6 ± 14.2	60.3 ± 14.4	<0.001
Female gender, *n* (%)	1,512 (51.9%)	897 (59.9%)	615 (43.5%)	<0.001
Smoking history, *n* (%)	217 (7.5%)	110 (7.3%)	107 (7.6%)	0.873
Drinking history, *n* (%)	130 (4.5%)	70 (4.7%)	60 (4.2%)	0.637
**Severity of COVID19**, *n* (%)				<0.001
Mild	2,160 (74.2%)	1,174 (78.4%)	986 (69.7%)	
Severe	714 (24.5%)	319 (21.3%)	395 (27.9%)	
Critical	38 (1.3%)	5 (0.3%)	33 (2.3%)	
**Comorbidities on admission**	1,236 (42.4%)	616 (41.1%)	620 (43.8%)	0.147
Hypertension, *n* (%)	910 (31.2%)	461 (30.8%)	449 (31.8%)	0.596
Cardiovascular disease, *n* (%)	219 (7.5%)	107 (7.1%)	112 (7.9%)	0.468
Diabetes, *n* (%)	392 (13.5%)	205 (13.7%)	187 (13.2%)	0.757
Chronic pulmonary diseases, *n* (%)	141 (4.8%)	63 (4.2%)	78 (5.5%)	0.119
Cerebrovascular disease, *n* (%)	125 (4.3%)	55 (3.7%)	70 (5.0%)	0.107
Malignancy, *n* (%)	63 (2.2%)	25 (1.7%)	38 (2.7%)	0.078
Gastrointestinal diseases, *n* (%)	53 (1.8%)	22 (1.5%)	31 (2.2%)	0.186
Autoimmune disease, *n* (%)	20 (0.7%)	7 (0.5%)	13 (0.9%)	0.211
**Chronic liver diseases**, ***n*** **(%)**	68 (2.3%)	31 (2.1%)	37 (2.6%)	0.390
Hepatitis B virus infection, *n* (%)	58 (2.0%)	26 (1.7%)	32 (2.3%)	0.376
Hepatitis C virus infection, *n* (%)	8 (0.3%)	5 (0.3%)	3 (0.2%)	0.785
Autoimmune liver disease, *n* (%)	2 (0.1%)	0 (0.0%)	2 (0.1%)	0.454
**Clinical characteristics on admission**				
Fever (>37.5°C), *n* (%)	2,057 (70.6%)	1,029 (68.7%)	1,028 (72.7%)	0.020
Cough, *n* (%)	2,001 (68.7%)	998 (66.6%)	1,003 (70.9%)	0.014
Expectoration, *n* (%)	420 (14.4%)	186 (12.4%)	234 (16.5%)	<0.001
Dyspnea, *n* (%)	1,394 (47.9%)	651 (43.5%)	743 (52.5%)	<0.001
Fatigue, *n* (%)	1,461 (50.2%)	697 (46.5%)	764 (54.0%)	<0.001
Myalgia, *n* (%)	774 (26.6%)	357 (23.8%)	417 (29.5%)	<0.001
Anorexia, *n* (%)	523 (18.0%)	228 (15.2%)	295 (20.9%)	<0.001
Nausea, *n* (%)	63 (2.2%)	38 (2.5%)	25 (1.8%)	0.194
Vomiting, *n* (%)	47 (1.6%)	31 (2.1%)	16 (1.1%)	0.063
Abdominal pain, *n* (%)	31 (1.1%)	17 (1.1%)	14 (1.0%)	0.842
Diarrhea, *n* (%)	126 (4.3%)	56 (3.7%)	70 (5.0%)	0.130
Headache, *n* (%)	54 (1.9%)	30 (2.0%)	24 (1.7%)	0.636
Dizziness, *n* (%)	36 (1.2%)	24 (1.6%)	12 (0.8%)	0.095
Disorders of consciousness, *n* (%)	19 (0.7%)	5 (0.3%)	14 (1.0%)	0.049
Systolic blood pressure (mmHg)	129.7 ± 16.2	130.3 ± 16.6	129.1 ± 15.7	0.059
Diastolic blood pressure (mmHg)	80.8 ± 11.6	81.5 ± 11.3	80.2 ± 11.9	<0.001
Heart rate (beat per minute)	86.8 ± 13.4	87.2 ± 13.3	86.3 ± 13.5	0.091
Respiratory rate (breaths per minute)	20.4 ± 3.0	20.1 ± 2.7	20.6 ± 3.3	<0.001
**Chest radiography or CT on admission**, *n* (%)				<0.001
Normal	50 (1.7%)	33 (2.2%)	17 (1.2%)	
Interstitial pneumonia	1,389 (47.7%)	707 (47.2%)	682 (48.2%)	
Ground glass opacity	1,362 (46.8%)	719 (48.0%)	643 (45.5%)	
Local consolidation	69 (2.4%)	29 (1.9%)	40 (2.8%)	
Bilateral consolidation	42 (1.4%)	10 (0.7%)	32 (2.3%)	
**Laboratory examination on admission**				
White blood cell count (×10^9^/L)	6.2 ± 2.8	5.9 ± 2.1	6.5 ± 3.4	<0.001
Neutrophil count (×10^9^/L)	4 ± 2.7	3.6 ± 1.6	4.5 ± 3.5	<0.001
Lymphocyte count (×10^9^/L)	0.7 ± 2.6	0.6 ± 1.2	0.8 ± 3.6	0.062
Hemoglobin (g/L)	124.2 ± 18.4	124.9 ± 16.2	123.4 ± 20.4	0.025
Platelet count (×10^9^/L)	232.1 ± 82.4	225.3 ± 69.9	239.3 ± 93.3	<0.001
Alanine aminotransferase (ALT), U/L	33.0 ± 34.8	19.6 ± 8.4	47.3 ± 45.0	<0.001
ALT <40 U/L, *n* (%)	2,250 (77.3%)	1,498 (100.0%)	752 (53.2%)	
ALT 40–120 U/L, *n* (%)	584 (20.0%)	0 (0.0%)	584 (41.3%)	
ALT >120 U/L, *n* (%)	78 (2.7%)	0 (0.0%)	78 (5.5%)	
Aspartate aminotransferase (AST), U/L	25.7 ± 41.3	18.2 ± 5.2	33.6 ± 58.1	<0.001
AST <45 U/L, *n* (%)	2,691 (92.4%)	1,498 (100.0%)	1,193 (84.4%)	
AST 45–135 U/L, *n* (%)	200 (6.9%)	0 (0.0%)	200 (14.1%)	
AST >135 U/L, *n* (%)	21 (0.7%)	0 (0.0%)	21 (1.5%)	
Albumin (g/L)	37.8 ± 9.4	39.7 ± 9.4	35.8 ± 9.1	<0.001
Albumin >40 g/L, *n* (%)	826 (28.4%)	563 (37.6%)	263 (18.6%)	
Albumin 30–40 g/L, *n* (%)	1,925 (66.1%)	935 (62.4%)	990 (70.0%)	
Albumin <30 g/L, *n* (%)	161 (5.5%)	0 (0.0%)	161 (11.4%)	
Total bilirubin (TBIL), μmol/L	10.3 ± 6.6	9.6 ± 4.2	10.9 ± 8.3	<0.001
TBIL ≥26 μmol/L	52 (1.8%)	0 (0.0%)	52 (3.7%)	<0.001
Alkaline phosphatase (ALP), U/L	75.6 (32.8)	68.8 (17.6)	82.8 (42.3)	<0.001
ALP ≥125 U/L, *n* (%)	135 (4.6%)	0 (0.0%)	135 (9.5%)	<0.001
γ-Glutamyl transpeptidase (GGT), U/L	45.3 ± 49.3	26.9 ± 12.3	64.9 ± 64.0	<0.001
GGT <60 U/L, *n* (%)	2,376 (81.5%)	1,498 (99.8%)	878 (62.1%)	
GGT 60–180 U/L, *n* (%)	456 (15.8%)	0 (0.0%)	456 (32.2%)	
GGT >180 U/L, *n* (%)	80 (2.7%)	0 (0.0%)	80 (5.7%)	
Creatinine (μmol/L)	70.5 ± 48.8	67.4 ± 48.3	73.9 ± 49.1	<0.001
Blood urea nitrogen (mmol/L)	5.4 ± 12.1	5.2 ± 15.2	5.5 ± 7.4	0.496
Potassium (mmol/L)	4.4 ± 2.9	4.3 ± 1.5	4.4 ± 3.8	0.326
Sodium (mmol/L)	141.7 ± 24.3	141.7 ± 4.3	141.7 ± 34.5	0.93
D-dimer (μg/ml)	1.1 ± 4.4	0.7 ± 2.5	1.5 ± 5.7	<0.001
Prothrombin time (s)	10.8 ± 6	10.8 ± 5.3	10.8 ± 6.7	0.786
Activated partial thromboplastin time (s)	28.1 ± 6.8	27.8 ± 3.9	28.4 ± 8.8	<0.001
International normalized ratio	1.2 ± 3.1	1.2 ± 3.8	1.2 ± 2.1	0.838
Creatine kinase (U/L)	62.1 ± 64.2	59.1 ± 42.8	65.3 ± 80.8	<0.001
Lactate dehydrogenase (U/L)	198.5 ± 90	175.8 ± 64.2	222.5 ± 105.8	<0.001
Procalcitonin (ng/ml)	0.2 ± 0.7	0.1 ± 0.7	0.2 ± 0.7	<0.001
C-reactive protein (mg/L)	13.6 ± 30.4	5.5 ± 15.8	22.2 ± 38.6	<0.001
**Liver function tests during hospitalization**				
Peak aspartate aminotransferase (ALT), U/L	41.4 ± 60.3	31.6 ± 56.7	51.7 ± 62.3	<0.001
ALT <40 U/L, *n* (%)	2,057 (70.6%)	1,303 (87.0%)	754 (53.3%)	
ALT 40–120 U/L, *n* (%)	721 (24.8%)	156 (10.4%)	565 (40.0%)	
ALT >120 U/L, *n* (%)	134 (4.6%)	39 (2.6%)	95 (6.7%)	<0.001
Peak aspartate aminotransferase (AST), U/L	31.4 ± 50.1	27.2 ± 51.6	35.8 ± 48.1	<0.001
AST <45 U/L, *n* (%)	2,591 (89.0%)	1,409 (94.1%)	1,182 (83.6%)	
AST 45–135 U/L, *n* (%)	270 (9.2%)	67 (4.5%)	203 (14.4%)	
AST >135 U/L, *n* (%)	51 (1.8%)	22 (1.5%)	29 (2.1%)	<0.001
Nadir albumin, g/L	35.9 ± 5.5	37.6 ± 4.8	34.1 ± 5.5	<0.001
Albumin >40 g/L, *n* (%)	567 (19.5%)	391 (26.1%)	176 (12.4%)	
Albumin 30–40 g/L, *n* (%)	2,000 (68.7%)	1,015 (67.8%)	985 (69.7%)	
Albumin <30 g/L, *n* (%)	345 (11.8%)	92 (6.1%)	253 (17.9%)	<0.001
Peak total bilirubin (TBIL), μmol/L	11.8 ± 16.8	10.9 ± 8.0	12.7 ± 22.6	<0.001
TBIL ≥ 26 μmol/L, *n* (%)	103 (3.5%)	34 (2.3%)	69 (4.9%)	<0.001
Peak alkaline phosphatase (ALP), U/L	79.4 ± 42.8	73.1 ± 28.8	86.1 ± 53.0	<0.001
ALP ≥125 U/L, *n* (%)	166 (5.7%)	34 (2.3%)	132 (9.3%)	<0.001
Peak γ-glutamyl transpeptidase (GGT), U/L	49.1 ± 54.3	32.9 ± 30.9	66.3 ± 66.9	<0.001
GGT <60 U/L, *n* (%)	2,270 (78.0%)	1,396 (93.2%)	874 (61.8%)	
GGT 60–180 U/L, *n* (%)	560 (19.2%)	92 (6.1%)	468 (33.1%)	
GGT >180 U/L, *n* (%)	82 (2.8%)	10 (0.7%)	72 (5.1%)	<0.001

*COVID-19, coronavirus disease 2019, CT, computed tomography; LFTs, liver function tests. Plus-minus values are means ± standard deviation*.

**Table 2:** In-hospital treatment and outcomes according to normal vs. abnormal liver function on admission.

**Variable**	**All**	**Normal LFTs**	**Abnormal LFTs**	***P*-value**
	**(*n* = 2,912)**	**(*n* = 1,498)**	**(*n* = 1,414)**	
Antiviral therapy, *n* (%)	1,338 (45.9%)	594 (39.7%)	744 (52.6%)	<0.001
Include abidor, *n* (%)	1,191 (40.9%)	556 (37.1%)	635 (44.9%)	<0.001
Include ribavirin, *n* (%)	89 (3.1%)	22 (1.5%)	67 (4.7%)	<0.001
Include oseltamivir, *n* (%)	223 (7.7%)	63 (4.2%)	160 (11.3%)	<0.001
Include interferon, *n* (%)	235 (8.1%)	113 (7.5%)	122 (8.6%)	0.314
Antibiotics, *n* (%)	964 (33.1%)	372 (24.8%)	592 (41.9%)	<0.001
Quinolones, *n* (%)	699 (24.0%)	265 (17.7%)	434 (30.7%)	<0.001
Cephalosporins, *n* (%)	87 (3.0%)	14 (0.9%)	73 (5.2%)	<0.001
Macrolides, *n* (%)	31 (1.1%)	12 (0.8%)	19 (1.3%)	0.213
Traditional Chinese medicine, *n* (%)	2,627 (90.2%)	1,362 (90.9%)	1,265 (89.5%)	0.207
Immunoglobin, *n* (%)	134 (4.6%)	35 (2.3%)	99 (7.0%)	<0.001
Glucocorticoid therapy, *n* (%)	414 (14.2%)	116 (7.7%)	298 (21.1%)	<0.001
High flow nasal cannula, *n* (%)	1,771 (60.8%)	848 (56.6%)	923 (65.3%)	<0.001
Continuous renal replacement therapy, *n* (%)	10 (0.3%)	0 (0.0%)	10 (0.7%)	<0.001
Extracorporeal membrane oxygenation, *n* (%)	3 (0.1%)	0 (0.0%)	3 (0.2%)	0.228
Mechanical ventilation, *n* (%)	75 (2.6%)	13 (0.9%)	62 (4.4%)	<0.001
Non-invasive	28 (1.0%)	7 (0.5%)	21 (1.5%)	
Invasive	12 (0.4%)	3 (0.2%)	9 (0.6%)	
Non-invasive + Invasive	35 (1.2%)	3 (0.2%)	32 (2.3%)	
Admission or transfer to ICU, *n* (%)	106 (3.6%)	18 (1.2%)	88 (6.2%)	<0.001
Death, *n* (%)	61 (2.1%)	7 (0.5%)	54 (3.8%)	<0.001
Length of hospital stay (days)	14.9 ± 9.0	12.8 ± 7.4	17.2 ± 9.9	<0.001
Composite endpoin^†^	121 (4.2%)	22 (1.5%)	99 (7.0%)	<0.001

*Plus-minus values are means ± standard deviation. ICU, intensive care unit, LFTs, Liver function tests*.

† *The composite end-points consist of admission to intensive care unit, mechanical ventilation, and/or death*.

**Figure 1:**
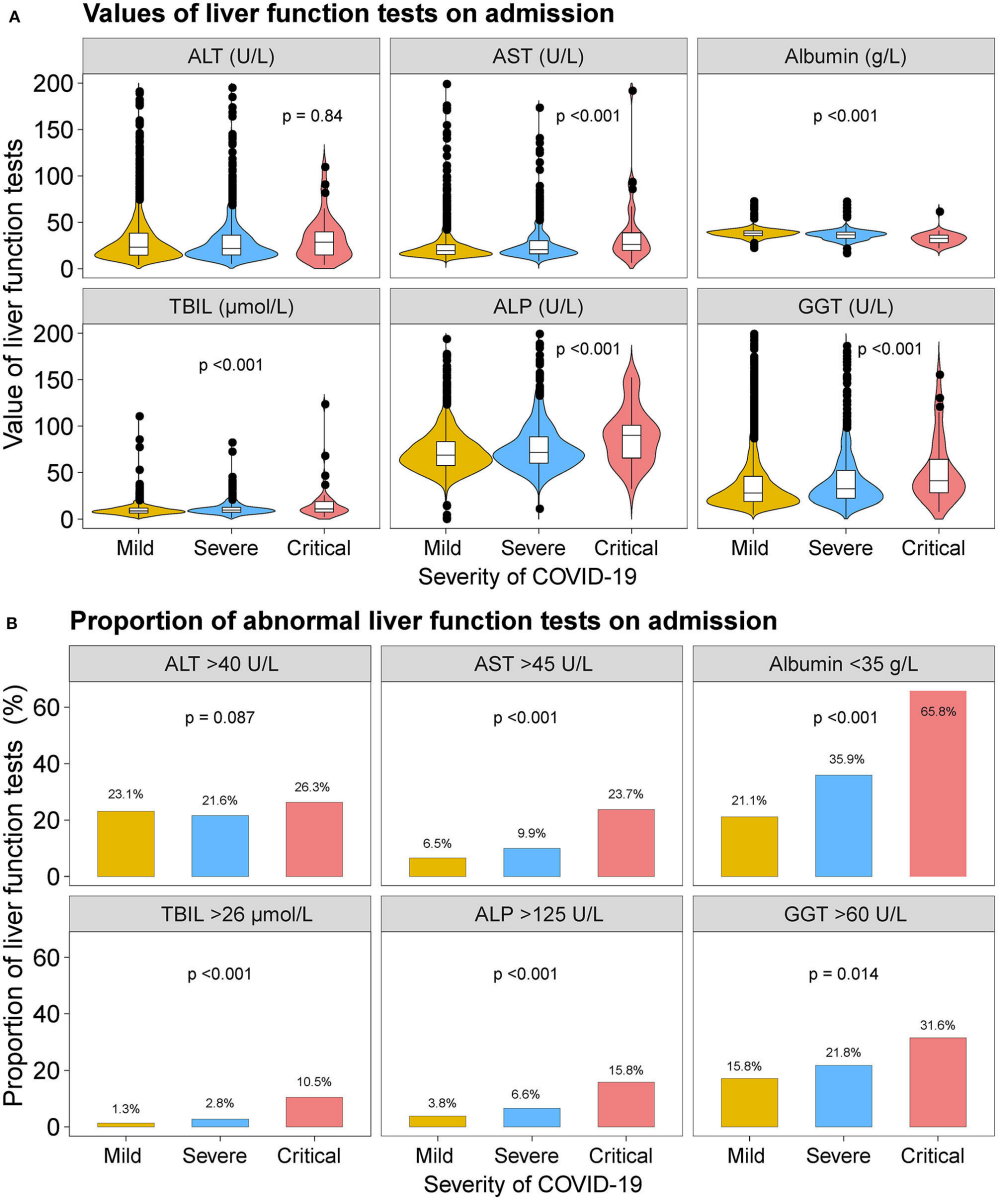
Liver function tests on admission. **(A)** Violin and box plots showing the median values of ALT, AST, albumin, TBIL, ALP, and GGT by severity of the COVID-19 disease. **(B)** Bar plots showing the proportion of abnormal ALT, AST, albumin, TBIL, ALP, and GGT tests on admission by severity of the COVID-19 disease. ALT, alanine aminotransferase; AST, aspartate transaminase; ALP, alkaline phosphatase; GGT, gamma-glutamyltransferase; TBIL, total bilirubin abnormal.

When stratified according to the severity of COVID-19 infection, patients with severe or critical COVID-19 had higher median values of AST, TBIL, ALP, and GGT and lower median value of albumin. The proportion of patients with abnormal AST, albumin, TBIL, ALP, and GGT were higher in severe or critical cases (Figure 1 and Supplementary Tables 1, 2). Nevertheless, the median value of ALT and the proportion of patients with abnormal ALT were not significantly across the severity of COVID-19.

### Dynamic Changes of Liver Functions

**Figure 2:**
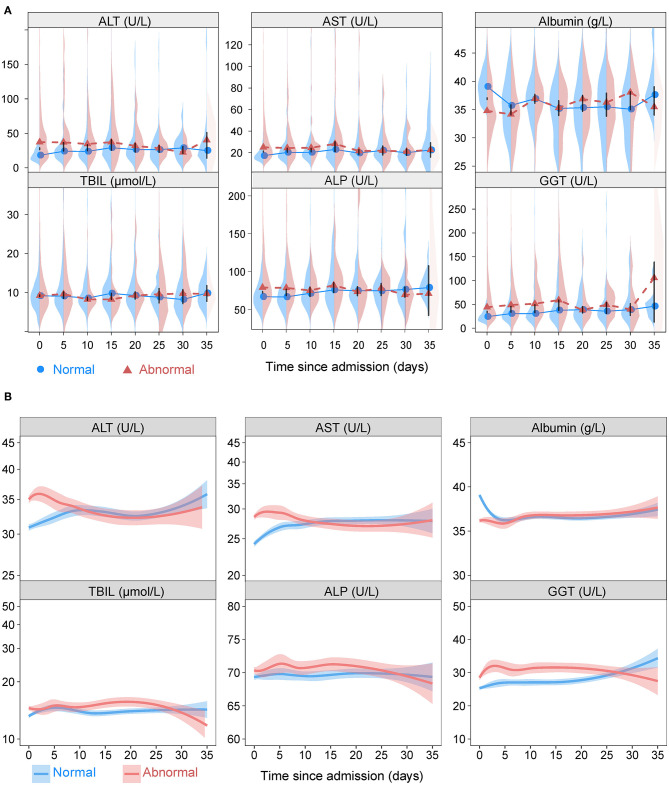
The liver function variations. **(A)** Longitudinal back-to-back violin plots showing the variations of ALT, AST, albumin, TBIL, ALP, and GGT during follow-up stratified by presence or absence of abnormal liver function tests on admission. Circles and triangles indicate medians. The black vertical bars have lengths equal to one-half the length of the 95% confidence interval for the difference in medians. When this bar does not touch the circles and triangles, there is a significant difference in medians at the 0.05 level. **(B)** Smooth trajectories of mean ALT, AST, albumin, TBIL, ALP, and GGT by disease severity with 95% confidence band based on locally weighted scatterplot smoothing stratified by presence or absence of abnormal liver function tests on admission. ALT, alanine aminotransferase; AST, aspartate transaminase; ALP, alkaline phosphatase; GGT, gamma-glutamyltransferase; TBIL, total bilirubin abnormal.

Figure 2 depicts the dynamic trajectories of ALT, AST, albumin, TBIL, ALB, and GGT according to normal or abnormal LFTs on admission. The ALT, AST, TBIL, ALP, and GGT values in the abnormal LFTs group increased slightly within the first 5 days after admission and trended downwards thereafter, while those values in the normal LFTs group tended upwards for the entire in-hospital duration. The albumin values trended downwards in both groups within the first 5 days of hospitalization and then fluctuated slightly for the entire duration of follow-up.

When stratified according to the severity of COVID-19 (mild vs. severe/critical), the dynamic curves of LFTs showed downward trends of ALT, AST, TBIL, ALP, and GGT and a upward trend of albumin in both mild and severe/critical groups (Supplementary Figure 2). Furthermore, the values of ALT, AST, TBIL, ALP, and GGT were higher and albumin was lower in patents with the outcomes of death, ICU admission, and mechanical ventilation compared those without in most time-points (Supplementary Figures 3–5).

### Predictors of Peak (Nadir) Value of Liver Function Test During Hospitalization in COVID-19

**Figure 3:**
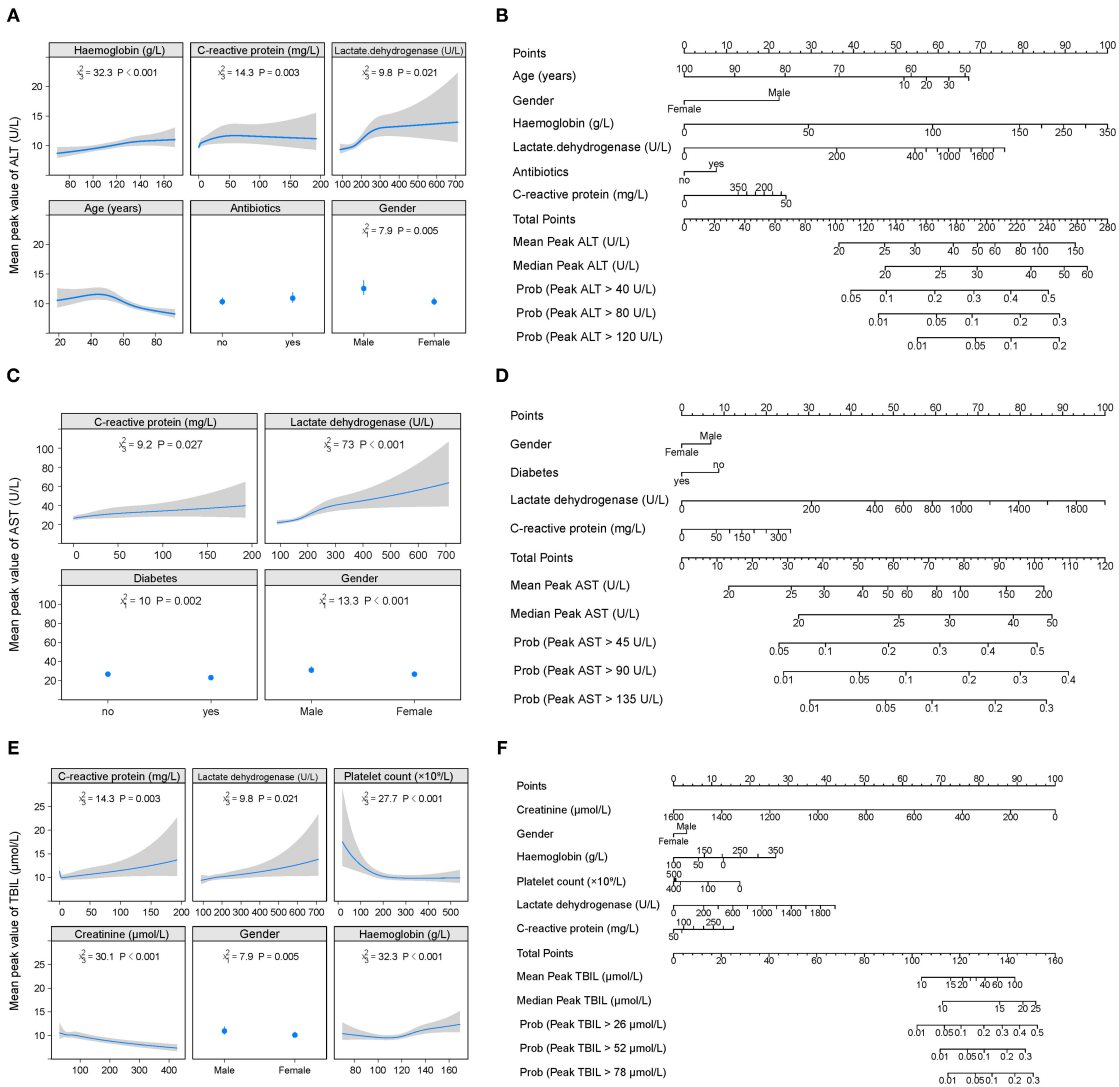
Factors and nomogram for predicting the peak values of ALT, AST, and TBIL during hospitalization. **(A, C, E)** Multivariable analysis of factors associated with the peak values of ALT, AST, and TBIL during hospitalization. The non-linearity of continuous variables were considered and analyzed with restricted cubic splines. **(B, D, F)** Nomogram for predicting the peak values of ALT, AST, and TBIL during hospitalization. To use the nomogram, first draw a vertical line to the top points row to assign points for each variable; then, add the points from each variable together and drop a vertical line from the total points row to obtain the median, mean values of peak ALT, AST, and TBIL during hospitalization as well as the probability of above the 1-, 2-, 3-time upper limit of normal of these parameters.

**Figure 4:**
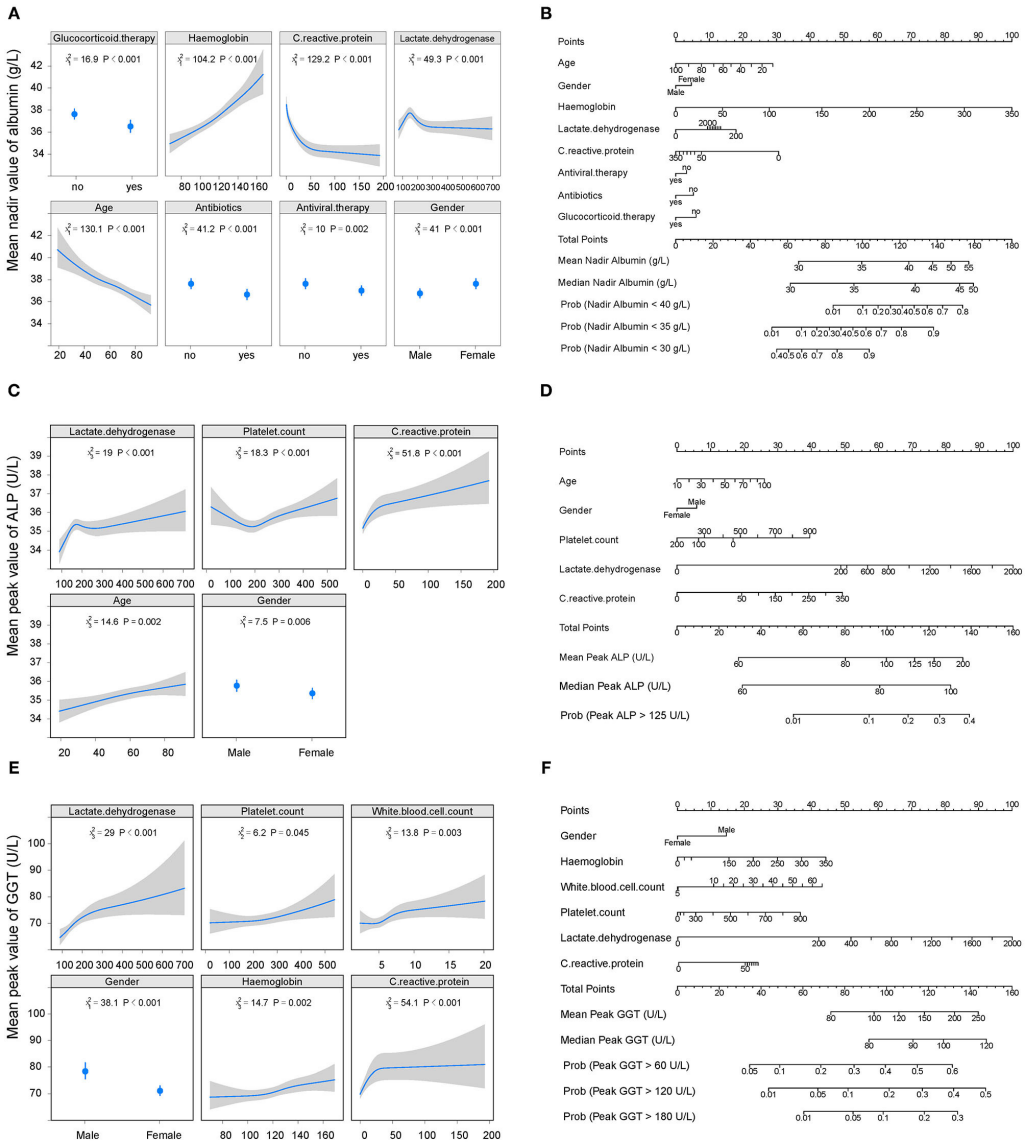
Factors and nomogram for predicting the nadir albumin and peak ALP, GGT during hospitalization. **(A, C, E)** Multivariable analysis of factors associated with nadir albumin and peak ALP, GGT during hospitalization. The non-linearity of continuous variables were considered and analyzed with restricted cubic splines. **(B, D, F)** Nomogram for predicting the peak values of nadir albumin and peak ALP, GGT during hospitalization. To use the nomogram, first draw a vertical line to the top points row to assign points for each variable; then, add the points from each variable together and drop a vertical line from the total points row to obtain the median, mean values of nadir albumin and peak ALP, GGT during hospitalization as well as the probability of above the 1-, 2-, 3-time upper limit of normal of these parameters. ALP, alkaline phosphatase; GGT, gamma-glutamyltransferase.

The cumulative probability model revealed the association between baseline characteristics and hospital treatment on peak ALT, AST, TBIL, ALP, GGT levels, and nadir albumin levels in the entire cohort (Supplementary Figure 6 and Figures 3, 4). Younger age, male gender, use of antibiotics, increased hemoglobin, increased C-reactive protein, and increased lactate dehydrogenase were factors positively associated with elevated ALT levels. Male gender, diabetes, higher C-reactive protein, and increased lactate dehydrogenase were the leading factors positively associated with elevated AST levels. Total bilirubin levels rise were tightly associated with male gender, decreased creatinine, decreased platelet count, increased C-reactive protein, and increased lactate dehydrogenase. Older age, male gender, antiviral, antibiotics, systemic corticosteroids use, hemoglobin reduction, C-reactive protein, and lactate dehydrogenase elevation were main factors positively correlated with decreased albumin levels. Alkaline phosphatase levels were closely linked with older age, male gender, platelet count, C-reactive protein, and lactate dehydrogenase elevation. Male gender, white blood cell, platelet count, hemoglobin, C-reactive protein, and lactate dehydrogenase increase were identified as factors positively associated with elevated GGT levels. C-reactive protein, lactate dehydrogenase platelet count, hemoglobin, and male gender were common factors positively associated with ALT, AST, TBIL, ALP, GGT elevation, and albumin reduction during hospitalization. To predict the peak (nadir) value of these LFTs, nomograms that incorporated the significant risk factors were established.

### Associations Between Abnormal Liver Function Test on Admission and Clinic Outcomes

**Figure 5:**
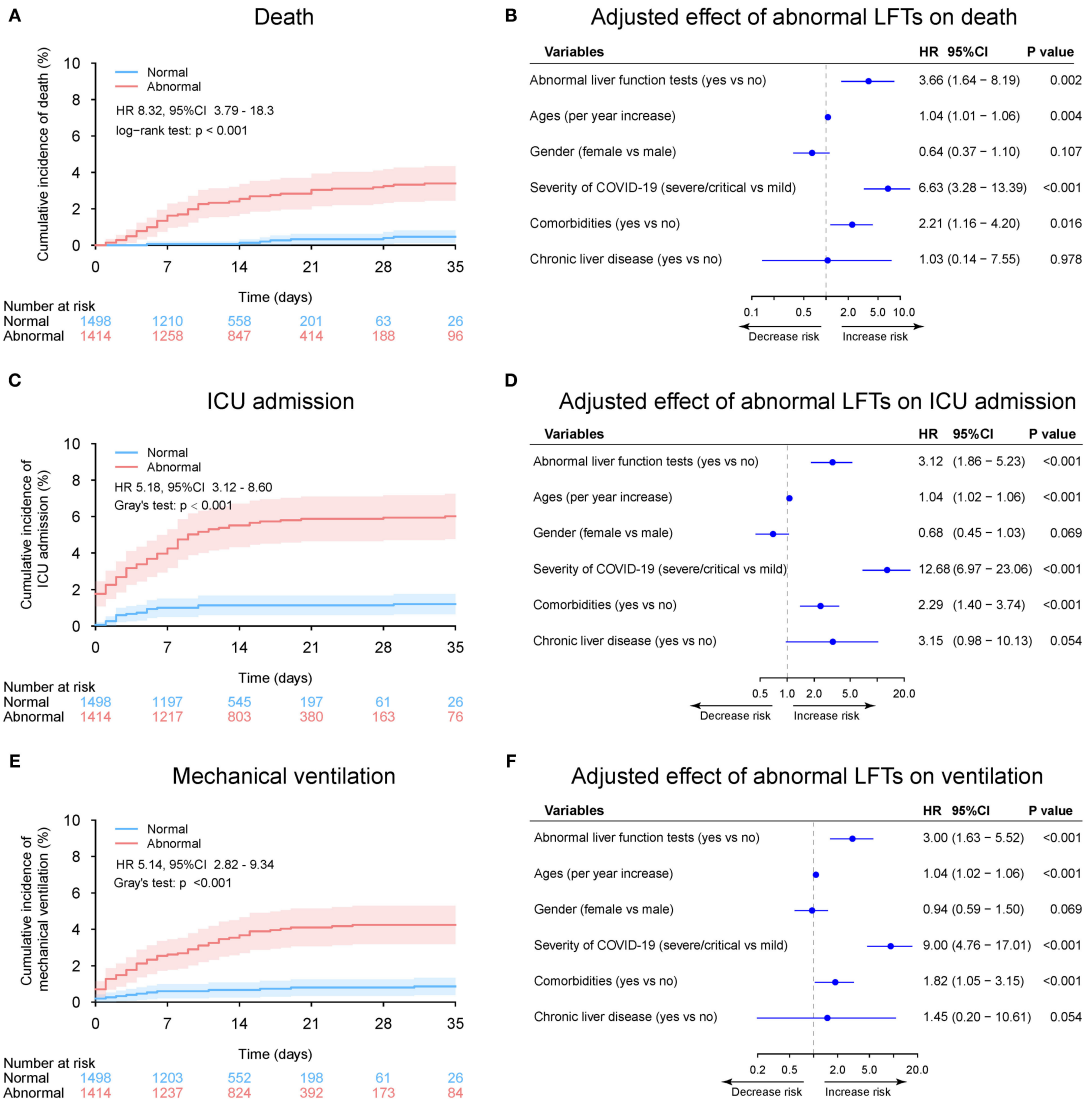
Outcome analysis according to admission abnormal vs. normal liver function tests (LFTs). **(A)** Cumulative incidence of death in patients with abnormal vs. normal LFTs on admission. **(B)** Independent effect (hazard ratio with 95% confidence intervals) of admission abnormal vs. normal LFTs on all-cause mortality adjusted for potential confounders using the Cox multivariable regression models. **(C)** Cumulative incidence of ICU admission in patients with abnormal vs. normal LFTs on admission based on competing risk approach (the Fine and Gray method) with death being the competing events. **(D)** Independent effect (hazard ratio with 95% confidence intervals) of admission abnormal vs. normal LFTs on ICU admission adjusted for potential confounders using the Cox multivariable regression models. **(E)** Cumulative incidence of mechanical ventilation in patients with abnormal vs. normal LFTs on admission based on competing risk approach (the Fine and Gray method) with death being the competing events. **(F)** Independent effect (hazard ratio with 95% confidence intervals) of admission abnormal vs. normal LFTs on mechanical ventilation adjusted for potential confounders using the Cox multivariable regression models. Comorbidities include hypertension, cardiovascular disease, diabetes, chronic pulmonary diseases, cerebrovascular disease, malignancy, and autoimmune disease. Chronic liver diseases include hepatitis B virus infection, hepatitis C virus infection, and autoimmune liver disease. LFTs, liver function tests; ICU, intensive care unit.

**Figure 6:**
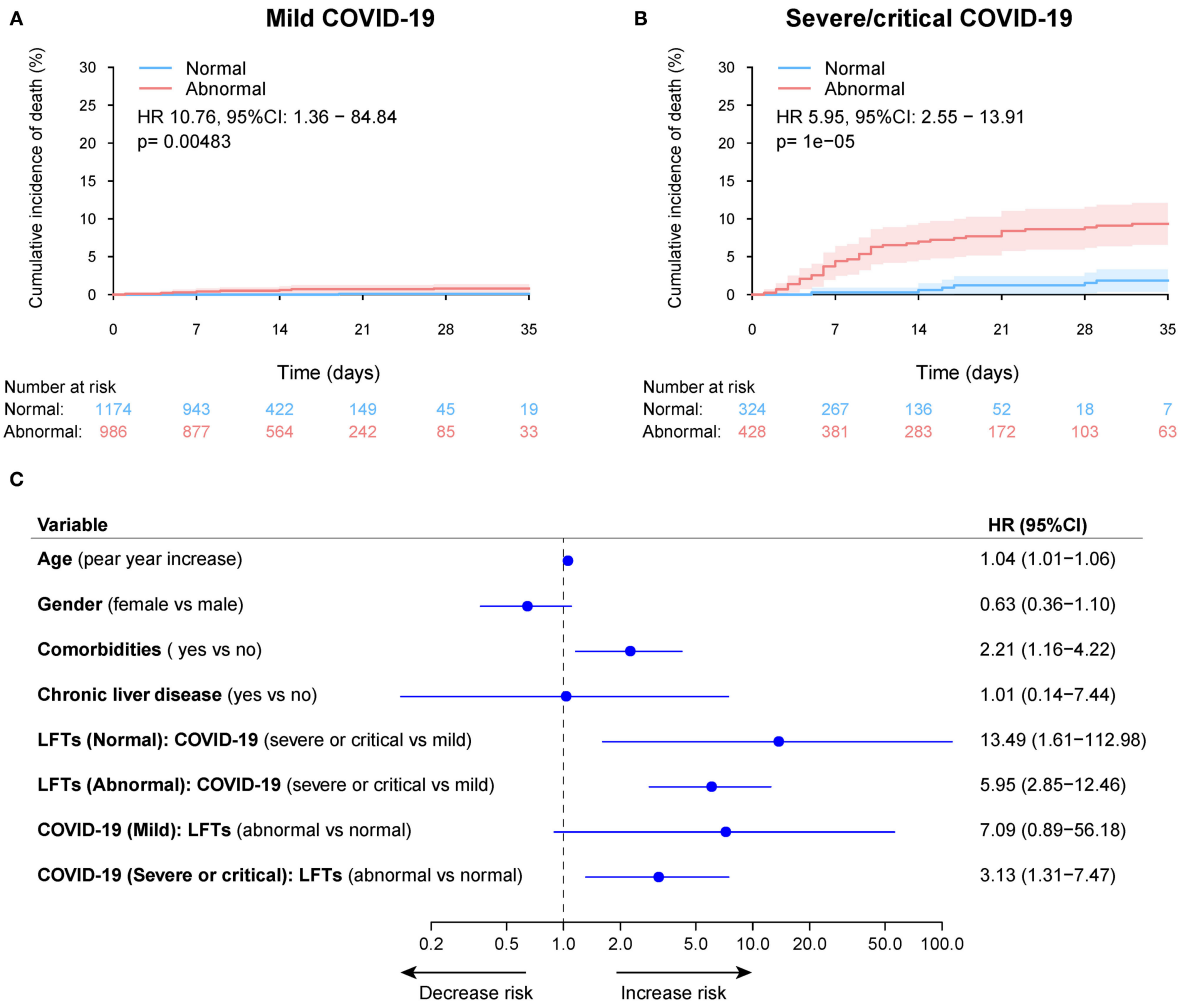
Cumulative incidence of death according to admission abnormal vs. normal liver function tests (LFTs) and severity of COVID-19 infection. **(A)** Cumulative incidence of death in patients with abnormal vs. normal LFTs on admission and mild COVID-19 infection. **(B)** Cumulative incidence of death in patients with abnormal vs. normal LFTs on admission and severe/critical COVID-19 infection. **(C)** Forest plot showing the interaction test of the LFTs (normal vs. abnormal) and severity of COVID-19 infection (mild vs. severe/critical) on death after adjustment for potential confounders using the Cox multivariable regression models. *P*_interaction_ = 0.521, showing a homogeneous effect of LFTs on death across the severity of COVID-19 infection. Comorbidities include hypertension, cardiovascular disease, diabetes, chronic pulmonary diseases, cerebrovascular disease, malignancy, and autoimmune disease. Chronic liver diseases include hepatitis B virus infection, hepatitis C virus infection, and autoimmune liver disease. COVID-19, coronavirus disease 2019; LFTs, liver function tests.

During a median 13 (IQR: 8–19) days of hospitalization, 61 patients (2.1%) died, 106 patients (3.6%) admitted or transfer to ICU, and 75 patients (2.6%) required mechanical ventilation (Table 2 and Supplementary Figure 7). The 30-day cumulative incidences of death was significantly higher in patients with abnormal LFTs on admission compared with those with normal LFTs (abnormal vs. normal: 3.3 vs. 0.47%; HR 8.32, [95%CI 3.79 −18.26]; *p* < 0.001, Figure 5A). Similarly, patients with abnormal LFTs on admission had a higher 30-day cumulative incidences of ICU admission (5.9 vs. 1.2%; HR 5.18 [95%CI 3.12–8.60]; *p* < 0.001; Figure 5C) and mechanical ventilation requirement (4.2 vs. 0.8%; HR 5.14 [95%CI 2.82–9.34]; *p* < 0.001, Figure 5E). This pattern persisted after adjusting for potential confounders, with the adjusted HRs of abnormal LFTs were 3.66 (95%CI 1.64–8.19, *p* = 0.002, Figure 5B) for death, 3.12 (95%CI 1.86–5.23, *p* < 0.001, Figure 5D) for ICU admission, and 3.00 (95%CI 1.63–5.52, *p* < 0.001, Figure 5F) for mechanical ventilation requirement. Furthermore, these effects were homogeneous across the severity of COVID-19 (*P*_interaction_ > 0.1 for all comparisons, Figure 6 and Supplementary Figures 8, 9). Notably, chronic liver disease was not associated with an increased risk of either of these adverse outcomes (Figures 5, 6 and Supplementary Figures 8, 9).

**Figure 7:**
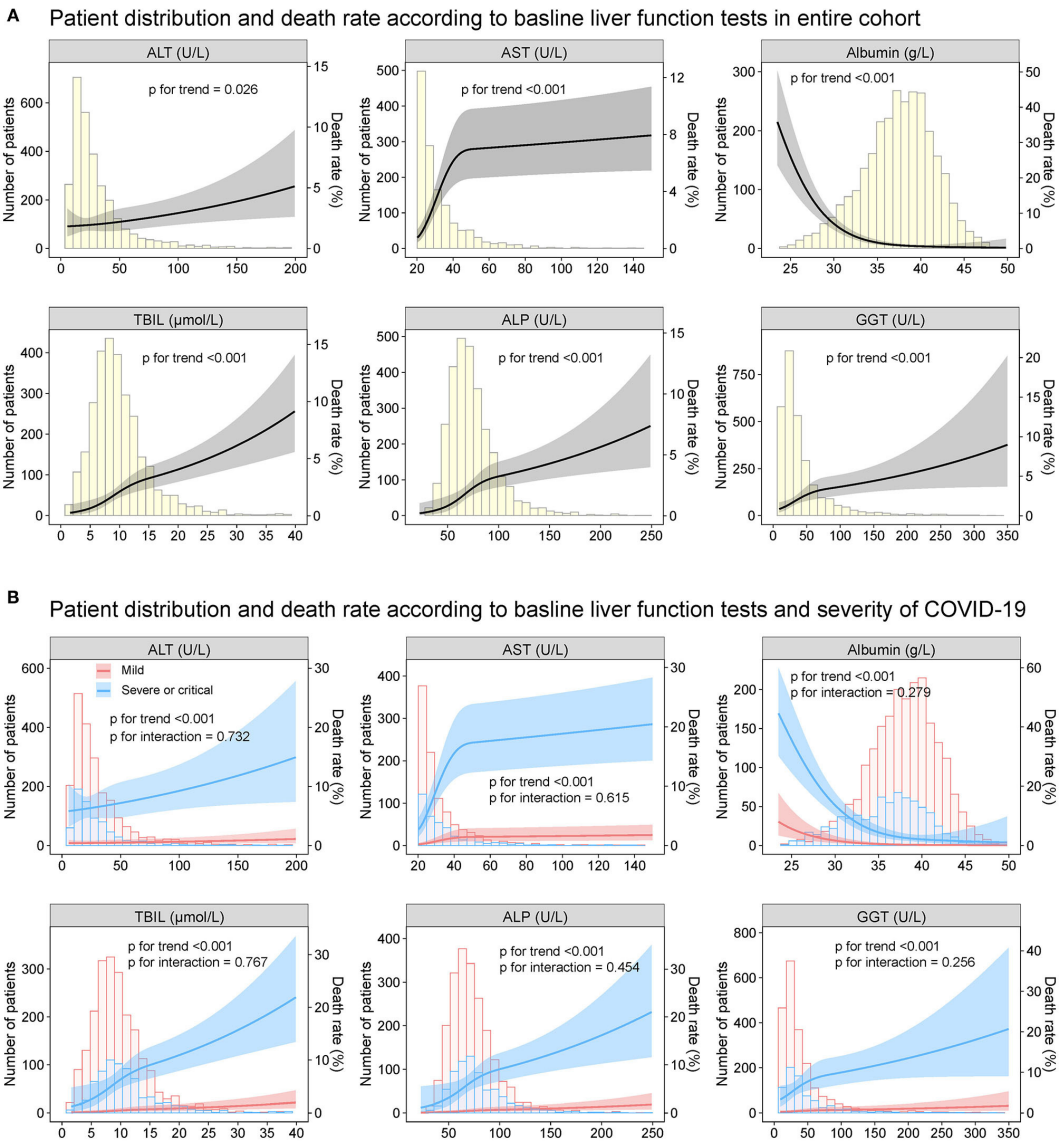
Patient distribution and death rate according to baseline liver function tests Patient distribution and death rate according to baseline ALT, AST, albumin, TBIL, ALP, and GGT **(A)** in entire cohort and **(B)** by severity of COVID19 infection (mild vs. severe/critical). Restricted cubic splines were generated using logistic regression models. ALT, alanine aminotransferase; AST, aspartate transaminase; ALP, alkaline phosphatase; GGT, gamma-glutamyltransferase; TBIL, total bilirubin abnormal.

The relationship between the baseline ALT, AST, albumin TBIL, ALP as well as GGT and death rate during hospitalization was depicted in Figure 7. The increased ALT, AST, TBIL, ALP, GGT, and decreased albumin on admission had a non-linear positive association with the risk of death, which was homogeneous across the severity of COVID-19. Similar results were observed for the secondary endpoint of ICU admission (Supplementary Figure 10) as well as mechanical ventilation requirement (Supplementary Figure 11).

**Figure 8:**
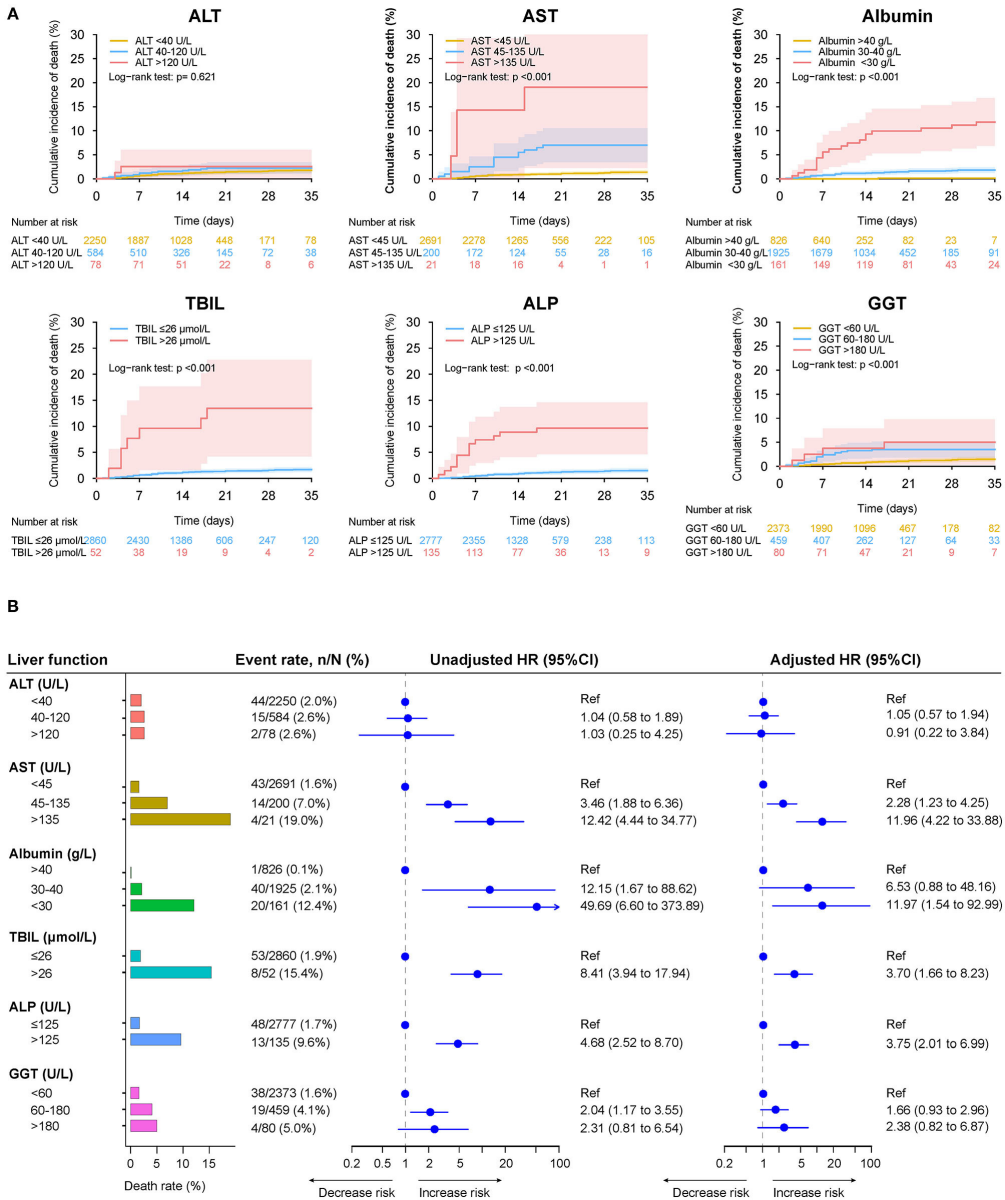
Mortality during hospitalization in patients with different level of baseline liver function tests. **(A)** Cumulative incidence of death in patients with different level of liver function test on admission. **(B)** Death rate in patients with different level of liver function test on admission, the unadjusted adjusted effect of liver function test at different level on the mortality during hospitalization. Adjusted HRs are derived from multivariate Cox regression models, adjusted for age, gender, comorbidities (hypertension, cardiovascular disease, diabetes, chronic pulmonary diseases, cerebrovascular disease, malignancy, autoimmune disease) and chronic liver diseases (hepatitis B virus infection, hepatitis C virus infection, autoimmune liver disease). ALT, alanine aminotransferase; ALP, alkaline phosphatase; AST, aspartate aminotransferase; CI, confidence interval; GGT, gamma-glutamyltransferase; HR, hazard ratio; TBIL, total bilirubin.

When stratified according to different levels of LFTs, abnormal levels of baseline AST, albumin, TBIL, ALP, and GGT were significantly associated with the risk of death, ICU admission, and mechanical ventilation (Figure 8 and Supplementary Figures 12, 13). Among them, AST over three-fold ULN and albumin <30 g/L had the highest risks of death, ICU admission, and mechanical ventilation. The elevation of ALT tended to be associated with increased risks of those outcomes. Nevertheless, the difference did not reach significance.

### Associations Between *De novo* Abnormal Liver Function Test During Hospitalization and Clinic Outcomes

**Figure 9:**
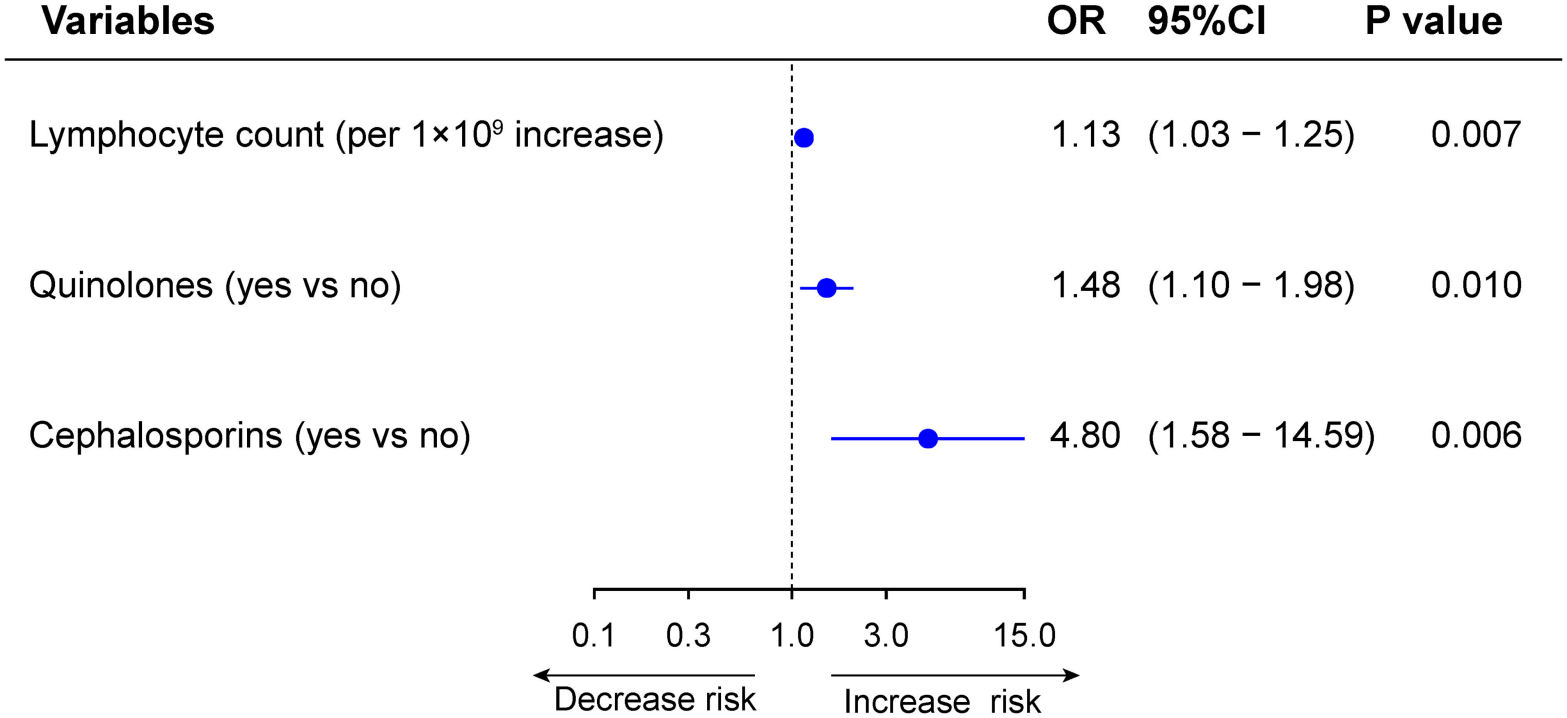
Multivariable analysis of factors associated with *de novo* abnormal vs. normal liver function tests (LFTs) during hospitalization.

**Figure 10:**
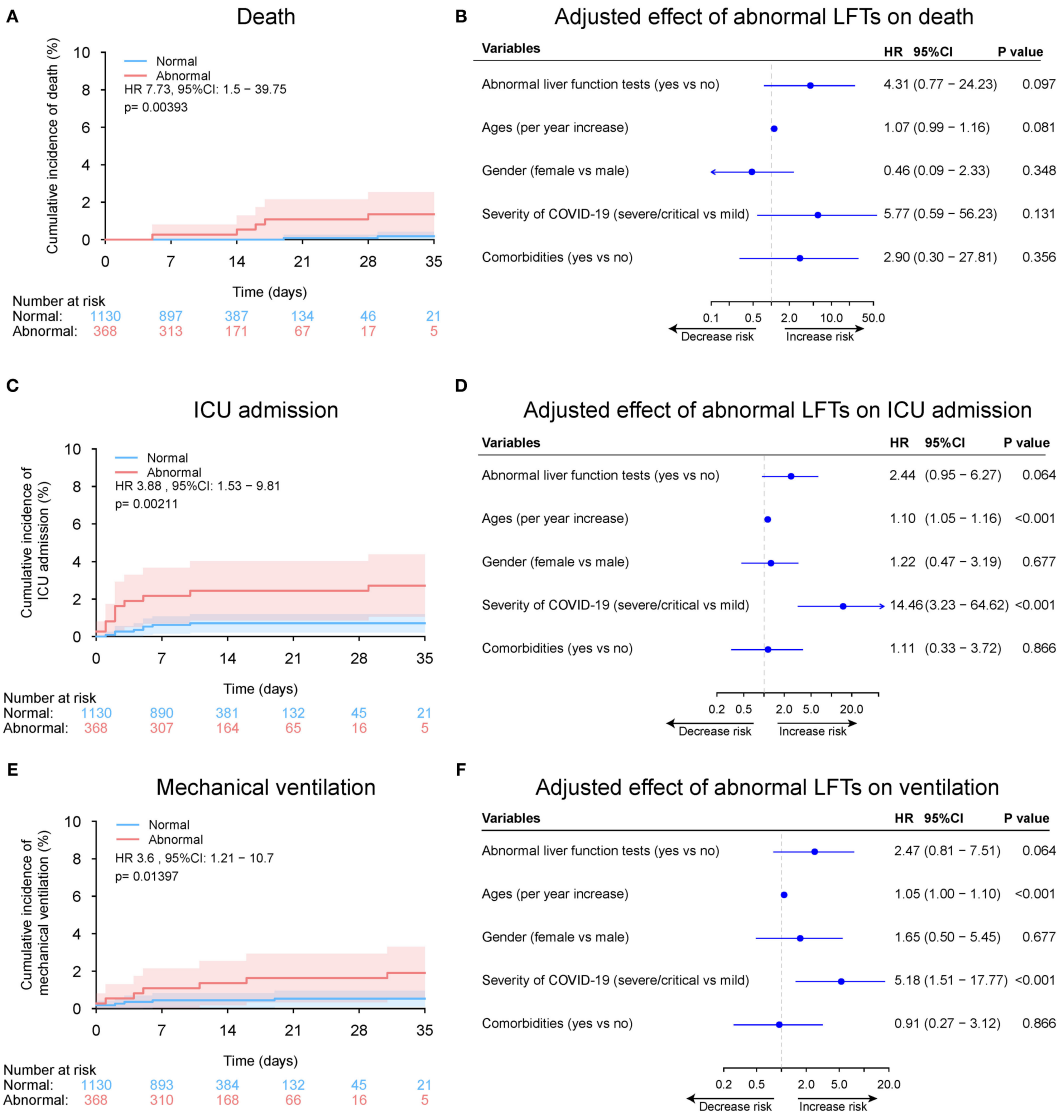
Outcome analysis according to *de novo* abnormal vs. normal liver function tests (LFTs) during hospitalization. **(A)** Cumulative incidence of death in patients with *de novo* abnormal vs. normal LFTs during hospitalization. **(B)** Independent effect (hazard ratio with 95% confidence intervals) of *de novo* abnormal vs. normal LFTs during hospitalization on all-cause mortality adjusted for potential confounders using the Cox multivariable regression models. **(C)** Cumulative incidence of ICU admission in patients with *de novo* abnormal vs. normal LFTs during hospitalization based on competing risk approach (the Fine and Gray method) with death being the competing events. **(D)** Independent effect (hazard ratio with 95% confidence intervals) of *de novo* abnormal vs. normal LFTs during hospitalization on ICU admission adjusted for potential confounders using the Cox multivariable regression models. **(E)** Cumulative incidence of mechanical ventilation in patients with *de novo* abnormal vs. normal LFTs during hospitalization based on competing risk approach (the Fine and Gray method) with death being the competing events. **(F)** Independent effect (hazard ratio with 95% confidence intervals) of *de novo* abnormal vs. normal LFTs during hospitalization on mechanical ventilation adjusted for potential confounders using the Cox multivariable regression models. Comorbidities include hypertension, cardiovascular disease, diabetes, chronic pulmonary diseases, cerebrovascular disease, malignancy, and autoimmune disease. Chronic liver diseases include hepatitis B virus infection, hepatitis C virus infection, and autoimmune liver disease. LFTs, liver function tests; ICU, intensive care unit.

Among the 1,498 patients with normal LFTs upon admission, 368 patients (24.6%) developed *de novo* abnormalities of LFTs (Supplementary Table 3). Univariable and multivariable logistic regression analysis showed that lymphocyte count (OR 1.13, 95%CI: 1.03–1.25, *p* = 0.007), use of quinolones (OR 1.48, 95%CI: 1.10–1.98, *p* = 0.010), and cephalosporins (OR 4.80, 95%CI: 1.58–14.59, *p* = 0.006) were independently were associated with *de novo* abnormalities of LFTs (Supplementary Figure 14 and Figure 9). Compared with those without *de novo* abnormal LFTs, patients with *de novo* abnormal LFTs had higher risk of death, ICU admission as well as the mechanical ventilation requirement. The trends persisted after adjusting for potential confounders, but the differences were not significant (Supplementary Table 4 and Figure 10).

### Associations Between Peak (Nadir) Liver Function Test During Hospitalization and Clinic Outcomes

**Figure 11:**
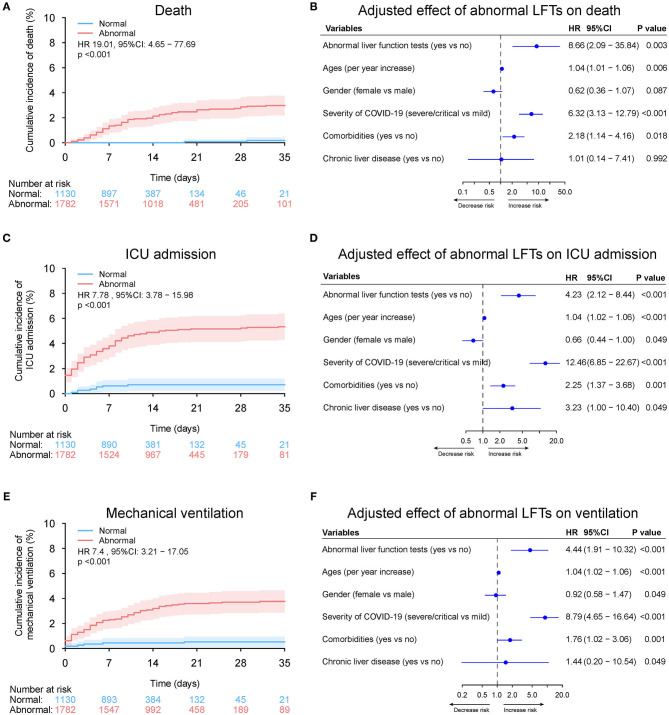
Outcome analysis according to abnormal vs. normal liver function tests (LFTs) during hospitalization in entire cohort. **(A)** Cumulative incidence of death in patients with abnormal vs. normal LFTs during hospitalization in entire cohort. **(B)** Independent effect (hazard ratio with 95% confidence intervals) of abnormal vs. normal LFTs during hospitalization on all-cause mortality adjusted for potential confounders using the Cox multivariable regression models. **(C)** Cumulative incidence of ICU admission in patients with abnormal vs. normal LFTs during hospitalization based on competing risk approach (the Fine and Gray method) with death being the competing events. **(D)** Independent effect (hazard ratio with 95% confidence intervals) of abnormal vs. normal LFTs during hospitalization on ICU admission adjusted for potential confounders using the Cox multivariable regression models. **(E)** Cumulative incidence of mechanical ventilation in patients with abnormal vs. normal LFTs during hospitalization based on competing risk approach (the Fine and Gray method) with death being the competing events. **(F)** Independent effect (hazard ratio with 95% confidence intervals) of abnormal vs. normal LFTs during hospitalization on mechanical ventilation adjusted for potential confounders using the Cox multivariable regression models. Comorbidities include hypertension, cardiovascular disease, diabetes, chronic pulmonary diseases, cerebrovascular disease, malignancy, and autoimmune disease. Chronic liver diseases include hepatitis B virus infection, hepatitis C virus infection, and autoimmune liver disease. LFTs, liver function tests; ICU, intensive care unit.

**Figure 12:**
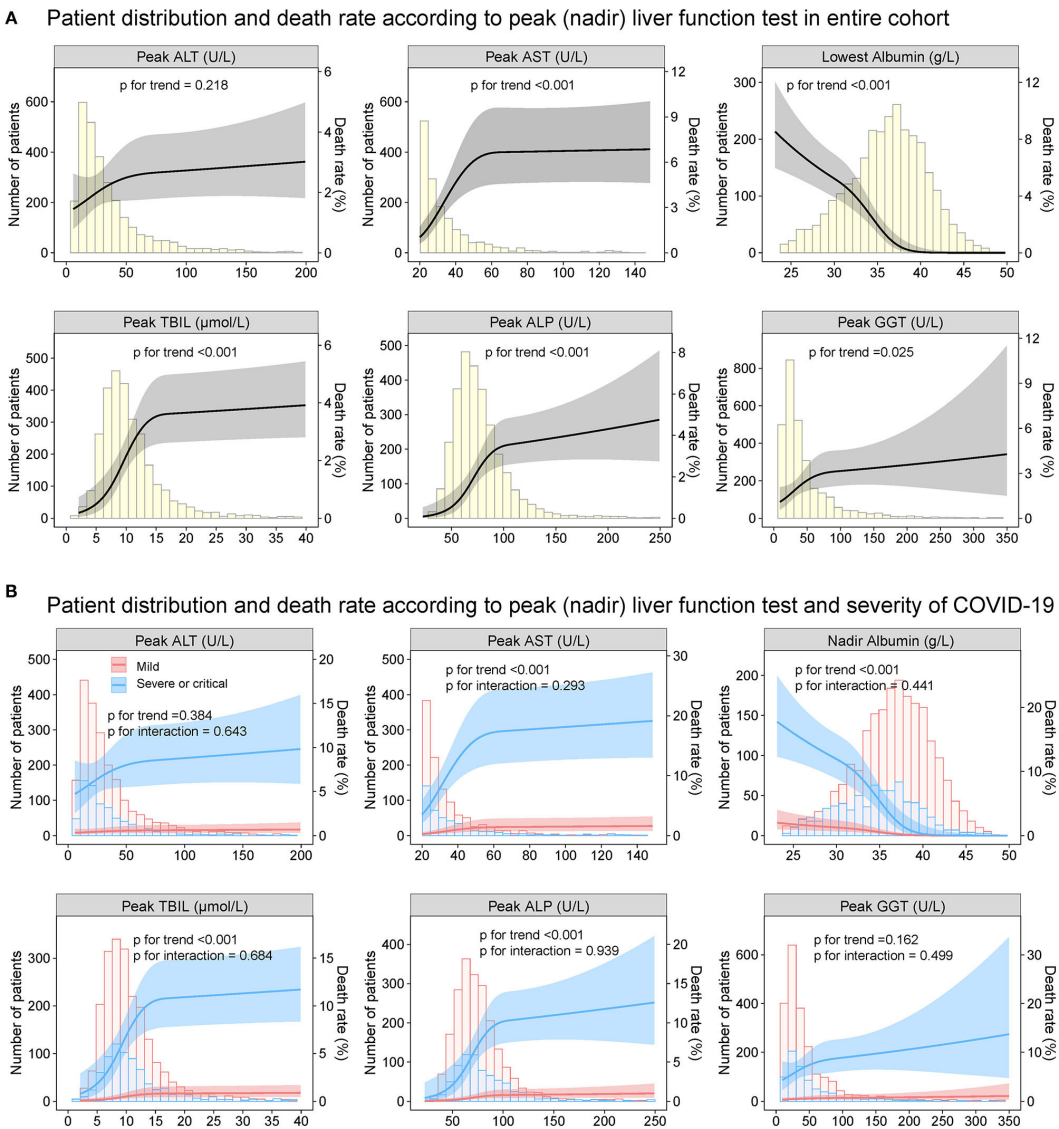
Patient distribution and death rates according to peak (nadir) liver function test in entire cohort. Patient distribution and death rate according to peak ALT, peak AST, nadir albumin, peak TBIL, peak ALP, and peak GGT during hospitalization **(A)** in entire cohort **(B)** by severity of COVID-19 infection (mild vs. severe/critical). Restricted cubic splines were generated using logistic regression models. ALT, alanine aminotransferase; AST, aspartate transaminase; ALP, alkaline phosphatase; COVID-19, coronavirus disease 2019; GGT, gamma-glutamyltransferase; TBIL, total bilirubin.

**Figure 13:**
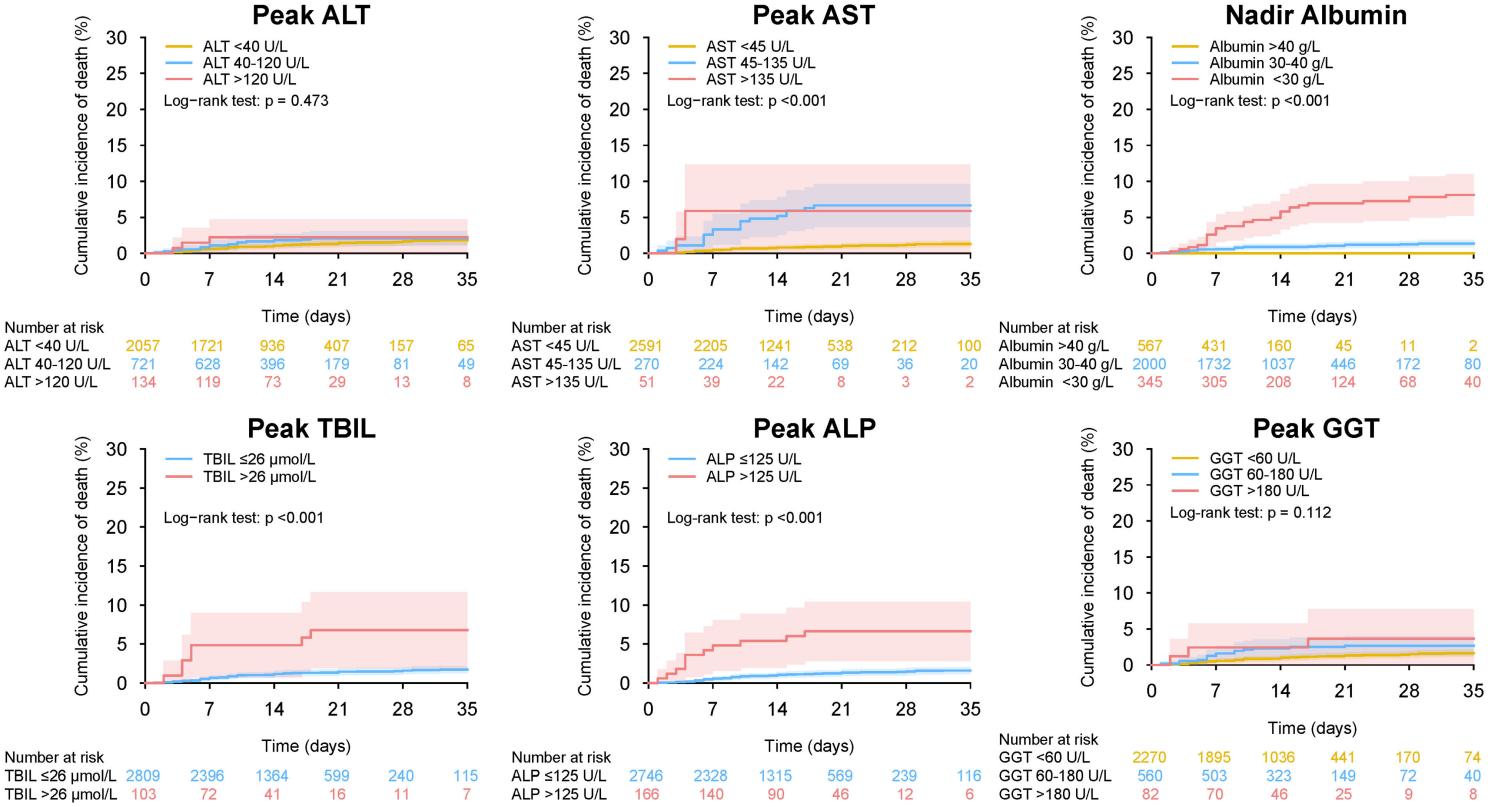
Mortality during hospitalization in patients with different level of peak (nadir) liver function test in entire cohort. Cumulative incidence of death during hospitalization in patients with different level of peak ALT, peak AST, nadir albumin, peak TBIL, peak ALP, and peak GGT during hospitalization. ALT, alanine aminotransferase; AST, aspartate transaminase; ALP, alkaline phosphatase; GGT, gamma-glutamyltransferase; TBIL, total bilirubin abnormal.

Overall, 1,782 patients (61.2%) had abnormal LFTs during hospitalization in the entire cohort. Peak ALT, AST, TBIL, ALP, and GGT above ULN was observed in 855 (29.4%), 321 (11.0%), 103 (3.5%), 166 (5.7%), and 642 (22.0%) patients, respectively. Nadir albumin <35 g/L was observed in 1,114 (38.3%) patients during hospitalization. The baseline characteristics of patients grouped according to LFTs abnormalities during hospitalization are shown in Supplementary Table 5. Compared with those with normal liver function impairment during hospitalization, patients with abnormal LFTs had severer COVID-19 disease and more common of respiratory and digestive symptoms. Similar to baseline LFTs abnormality, abnormal LFTs during hospitalization, peak AST, TBIL, ALP, GGT, and nadir albumin but not peak ALT were significantly (or a trend toward) associated with adverse outcomes of COVID-19 (Figures 11–13, Supplementary Tables 6, 7, and Supplementary Figures 15–19).

## Discussion

In this observational study of 2,912 hospitalized patients with COVID-19, we present the patterns and trajectories of LFTs as well as depict their clinical significance. The major findings were: (i) the derangement of liver function was generally mild (1–2 time of ULN) in non-severe patients but more frequent and to a greater extent in patients with severe/critical COVID-19 infection; (ii) Pattern of LFTs abnormality is predominantly hepatocellular rather than cholestatic; (iii) abnormality of LFTs was transient and tended to resolve over time; (iv) common factors associated with the peak (nadir) LFTs were C-reactive protein, lactate dehydrogenase, platelet count, hemoglobin, and male gender; (v) abnormal LFTs (AST, albumin, TBIL, ALP, and GGT but not ALT) were independently associated with increased risks of mortality, ICU admission, and mechanical ventilation requirement, which was homogeneous across the severity of COVID-19 infection. The strengths and novelties of the current study lie in: (i) use of a death-based primary endpoint, which is an objectively assessed and clinically relevant endpoint; (ii) a large sample size which allow providing estimates with narrow CIs; (iii) multivariate and subgroup analysis, which permitted adjustment for potential confounding factors and explore the effect homogeneity; (iv) adopting not only categorized but continuous LFT analysis; (v) comprehensive liver function parameters and outcomes analyses; (vi) differentiation between baseline and in-hospital elevations of liver enzymes; (vii) significant amount of data on pre-existing liver disease and therapies used during hospitalization.

In our cohort, 48.6% had abnormal liver biochemistries at admission and 61.2% had liver biochemistries derangement during hospitalization, which was slightly higher than what has been reported in the literature (34, 35). Disparity may be attributed to the diverse definition of abnormality of LFTs. Indeed, the liver enzymes (ALT, AST, ALP, and GGT) elevation observed here is similar to those in previous cohorts (11–27). Nevertheless, the hypoalbuminemia was considered as part of abnormal LFTs in our definition while it was not included in most previous studies. As in other reports (11–27), liver enzyme elevations in COVID-19, even in the severe COVID-19 category, are mild-to-moderate in most of the cases, and the pattern of abnormal liver biochemistries was characterized by slight increases in hepatocyte-related enzymes, including ALT and AST, with accompanying GGT elevation. Pure cholestatic alterations characterized by ALP elevation were rare, and an increase in TBIL was less commonly observed (36, 37). However, significant hypoalbuminemia was observed, particularly among patients with severe COVID-19 disease. The possible explanation might be that albumin is a negative acute phase reactant rather than a manifestation of a hepatic synthetic dysfunction.

Furthermore, when stratifying according to disease severity of COVID-19 infection, we found that the AST, TBIL, ALP, and GGT were elevated more frequently and to a greater extent in patients with severe COVID-19 compared to those with mild disease. However, ALT elevation was not significantly higher in the severe/critical patients. This observation may be related to the mechanism of LFTs abnormality. Available evidence suggests that hepatic involvement in COVID-19 could be related to the direct cytopathic effect of the virus, an uncontrolled immune reaction, sepsis, or drug-induced liver injury (2, 11, 37, 38). The postulated mechanism of viral entry is through the host angiotensin-converting enzyme 2 (ACE2) receptors (39, 40). However, the ACE2 receptor is much more heavily expressed in cholangiocytes than in hepatocytes. Furthermore, the concentrations of serum ALP was normal in most patients with COVID-19, suggest the most common mechanism of liver damage is not due to a direct cytopathic effect of the SARS-CoV-2 virus. Our analysis showed that peak (nadir) liver function markers were commonly correlated with the direct or indirect markers of inflammation (C-reactive protein, lactate dehydrogenase, platelet count, hemoglobin at baseline), which support the point that most cases of liver derangement may reflect sepsis related cholestasis and inflammatory changes, or hepatotoxicity from concomitant medications (41, 42). Furthermore, studies have confirmed increased NETosis, a form of non-apoptotic and highly immunogenic cell death causing bystander damage and coagulation changes, accompanies disease severity (42, 43). It can be imagined that the alteration of immune balance occurs with increased severity of COVID-19, thus explaining why increases in serum AST, ALP, and TBIL levels but not ALT tend to parallel the severity of pulmonary disease, in an analogous fashion to patterns seen in sepsis (44). Lymphocyte count, use of quinolones and cephalosporins were independently were associated with *de novo* abnormalities of LFTs during hospitalization, suggesting drug-induced liver injury should not be overlooked in patients with COVID-19. With further analysis of longitudinal patterns, we found that the abnormality of LFTs manifested as transient elevation in most cases and liver involvement tended to resolve during prolonged disease course, indicating that supportive care alone might be sufficient to achieve liver recovery. Therefore, we advise checking baseline LFTs in all patients on admission and monitoring of LFTs throughout the hospitalization, particularly in patients undergoing drug therapy for COVID-19 with potential hepatotoxicity.

Our results showed that abnormalities of LFTs on admission as well during hospitalization were associated with death, ICU admission and mechanical ventilation requirement in COVID-19 patients. More importantly, these associations were independent from the most commonly described predictors of the evaluated outcomes in multivariable analysis. Furthermore, the effects of LFTs on the evaluated outcomes were homogeneous across the severity of COVID-19, suggesting the impact of LFTs on the evaluated outcomes were not modified by the severity of COVID-19. Several studies have reported on the association between the abnormal LFTs and severity of disease or outcomes, with conflicting results (11–27). Most of them reported the results of univariate analyses without appropriately adjust for potential confounders. Thus, it is unclear whether the influence of abnormal LFTs on the prognosis was real or mediated by its association with other co-existing diseases. A large multicenter study of 5,771 Chinese individuals showed that peak liver biochemistries (AST, ALT, ALP, and TBIL) predicted mortality, after adjusting for age, gender, and comorbidities in Cox regression model (18). Similarly, an Italian study with 565 inpatients showed that abnormality of LFTs (ALT, AST, ALP, GGT, and TBIL) observed at admission was independently associated to a composite endpoint of transfer to the ICU or death (24). In contrast, another Italian study by Ponziani et al. (22) suggested baseline liver test (AST, ALT, and GGT) abnormalities were associated with increased risk of ICU admission but not with mortality. The discrepancy might be due to the somewhat low incidence of death in the latter study, which may reduce the likelihood of association between LFTS and mortality of COVID-19, with a wide CI of the HR. Thus, patients with abnormal LFTs should be closely followed up due to the potential worse outcomes.

In our study, while AST, albumin, TBIL, ALP, and GGT were significantly associated with adverse outcomes, no such an association were observed in ALT. This was in agreement with the study by Hao et al. (27) showing no differences in the severity, discharge rate, and median hospitalization time between patients with and without ALT elevation. However, this finding is in contrast with two previous studies where higher peak ALT values were significantly associated with increased risk of mortality or discharge to hospice (OR = 1.14 or 1.43) (18, 19). The main reason for the discrepancy was not clear. Nevertheless, it should be noted that the association between ALT and death was not so strong (18, 19), and our patients were generally healthier compared with previously published cohorts.

The prevalence of chronic liver disease in our cohort was 2.3%, which is within the range (2–11%) reported in recent data from other cohorts (45–48). Previous studies showed that those with chronic liver disease are more likely to have more adverse outcomes and mortality when compared to those without (49–52). In our study, however, the presence of chronic liver disease was not significantly associated with disease progression and mortality, which may be due in part to the overall low numbers of patients with these disease entities. Another possible explanation may be that the severity of chronic liver disease in our patients is generally mild, with no patient having cirrhosis. Indeed, the term “chronic liver disease” constitutes a spectrum of patients with varying prognosis ranging from chronic hepatitis, cirrhosis, decompensated cirrhosis to acute-on-chronic liver failure that may differentially affect outcomes (53).

Our study has several limitations. First, the single-center nature may limit its representativeness. However, quality control was ensured because all the diagnostic and therapeutic algorithms were uniform. Second, potential bias in the selection of samples is inherent to its retrospective design. Nevertheless, we included all consecutive patients with confirmed COVID-19 admitted to the hospital, which minimizes the risk of selection bias. Third, although multivariate regression analyses were conducted to adjust for potential confounders, our findings may be biased due to unidentified confounding. Fourth, liver biochemistries and other important laboratory markers were not assessed daily on every patient because this was not required for clinical decision making. Fifth, our study patients represent an exclusively inpatient population. Therefore, this information may not be generalizable to outpatients. Sixth, this is an observational study. Thus, the association should not be regarded as causal effect. Seventh, alcohol abuse and hepatotoxic drug intake prior to development of COVID-19 have not been considered. Finally, with only a few cases of incompletely characterized chronic liver disease in this cohort, we cannot draw conclusions about hepatic impairment and other outcomes for those patients.

In conclusion, abnormal liver function was common and associated with adverse clinical outcomes in COVID-19 patients. Thus, clinicians should keep close monitoring of liver biochemistries and cautiously use appropriate medications with least hepatotoxicity in such patients. Due to the nature of such retrospective study, these results should be interpreted with caution and are needed to be confirmed in future large prospective studies.

## Data Availability Statement

The raw data supporting the conclusions of this article will be made available by the authors, without undue reservation.

## Ethics Statement

The studies involving human participants were reviewed and approved by National Health Commission of China and the institutional review board at Huoshenshan Hospital. Written informed consent for participation was not required for this study in accordance with the national legislation and the institutional requirements.

## Author Contributions

Study concept and design: YL and HX, acquisition of data: YL, XZ, YW, JZ, CM, XF, YM, YZ, LY, GH, and HX, analysis and interpretation of data, drafting of the manuscript, and statistical analysis: YL, critical revision of the manuscript for important intellectual content: HX and GH. All authors contributed to the article and approved the submitted version.

## Conflict of Interest

The authors declare that the research was conducted in the absence of any commercial or financial relationships that could be construed as a potential conflict of interest.

## Abbreviations

ACE2: angiotensin converting enzyme 2
ALT: alanine aminotransferase
ALP: alkaline phosphatase
AST: aspartate aminotransferase
CI: confidence interval
COVID-19: coronavirus disease 2019
GGT: gamma-glutamyltransferase
HR: hazard ratio
ICU: intensive care unit
LFTs: liver function tests
SARSCoV-2: severe acute respiratory syndrome coronavirus 2
TBIL: total bilirubin
ULN: upper limit of normal.
