# Supplementary material for: Abnormal Liver Function Tests Were Associated With Adverse Clinical Outcomes: An Observational Cohort Study of 2,912 Patients With COVID-19
Source: Front Med (Lausanne). 2021 Jun 9;8:639855. doi: 10.3389/fmed.2021.639855 (PMC8219933; doi:10.3389/fmed.2021.639855)
Supplement: Supplementary file 1 [file Presentation_1.pdf]

Supplemented to:

Abnormal liver function tests were associated with adverse clinical outcomes: an observational cohort study of 2912 patients with COVID-19

Authors:

Yong Lv [1#], Xiaodi Zhao [1#], Yan Wang [2#], Jingpu Zhu [3], Chengfei Ma [3], Xiaodong Feng [3], Yao Ma [3], Yipeng Zheng [3], Liyu Yang [3], Guohong Han [4*], Huahong Xie [1,5*]

# Yong Lv, Xiaodi Zhao, Yan Wang contributed equally to this work.

***** Huahong Xie, Guohong Han shared the senior and corresponding authors.

Corresponding author:

Huahong Xie, M.D., Ph.D.

State key Laboratory of Cancer Biology, National Clinical Research Center for Digestive Diseases and Xijing Hospital of Digestive Diseases, Fourth Military Medical University;

E-mail: fangfang1@fmmu.edu.cn

Tel: +86-29-84775221

Fax: +86-29-82539041;

Prof. Guohong Han,

Department of Liver Diseases and Digestive Interventional Radiology, Xi'an International Medical Center Hospital of Digestive Diseases, Northwestern University, No,777 Xitai Road, High-tech Zone, Xi'an, 710000, China.

Tel: +86-29-68302656, Fax: +86-29-68302656.

Email: applehghong@126.com

Table of Contents

Supplementary Materials and Methods 3
Supplementary Figure 1 4
Supplementary Figure 2 5
Supplementary Figure 3 7
Supplementary Figure 4 8
Supplementary Figure 5 9
Supplementary Figure 6 10
Supplementary Figure 7 11
Supplementary Figure 8 12
Supplementary Figure 9 13
Supplementary Figure 10 14
Supplementary Figure 11 15
Supplementary Figure 12 16
Supplementary Figure 13 18
Supplementary Figure 14 20
Supplementary Figure 15 21
Supplementary Figure 16 23
Supplementary Figure 17 24
Supplementary Figure 18 25
Supplementary Figure 19 26
Supplementary Table 1 27
Supplementary Table 2 29
Supplementary Table 3 30
Supplementary Table 4 33
Supplementary Table 5 34
Supplementary Table 6 37
Supplementary Table 7 38

Supplementary Materials and Methods

Missing values and multiple imputations

The number of patients missing baseline data never exceeded 153 (5.3%). Among them, only alanine aminotransferase (ALT) was missed in 18 (0.6%) patients, only aspartate aminotransferase (AST) was missed in 10 (0.3%) patients, only albumin was missed in 65 (2.2%) patients, total bilirubin (TBIL) was missed in 70 (2.4%) patients, only alkaline palkaline hosphatase (ALP) was missed in 70 (2.4%) patients and only gamma-glutamyltransferase [GGT] was missed in 71(2.4%) patients.

To use the complete set of patients for regression analysis, multiple imputations was performed with the *aregImpute* function from the *Hmisc* R package (*https://cran.r-project.org/web/packages/Hmisc/*). With this method, different bootstrap resamples are used for each imputation by fitting a flexible parametric additive regression model on a sample with replacement from the original data. This model is used to predict all of the original missing and non-missing values for the target variable for the current imputation. This produces 10 sets of complete data that subsequently are used for regression analysis. This was associated with less bias in subsequent regression analysis.

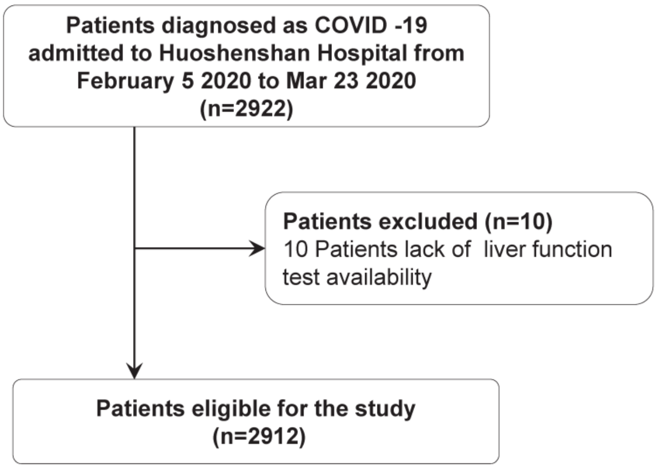

Supplementary Figure 1: Flowchart showing study design and patients disposition.

Abbreviations: COVID-19, coronavirus disease 2019.

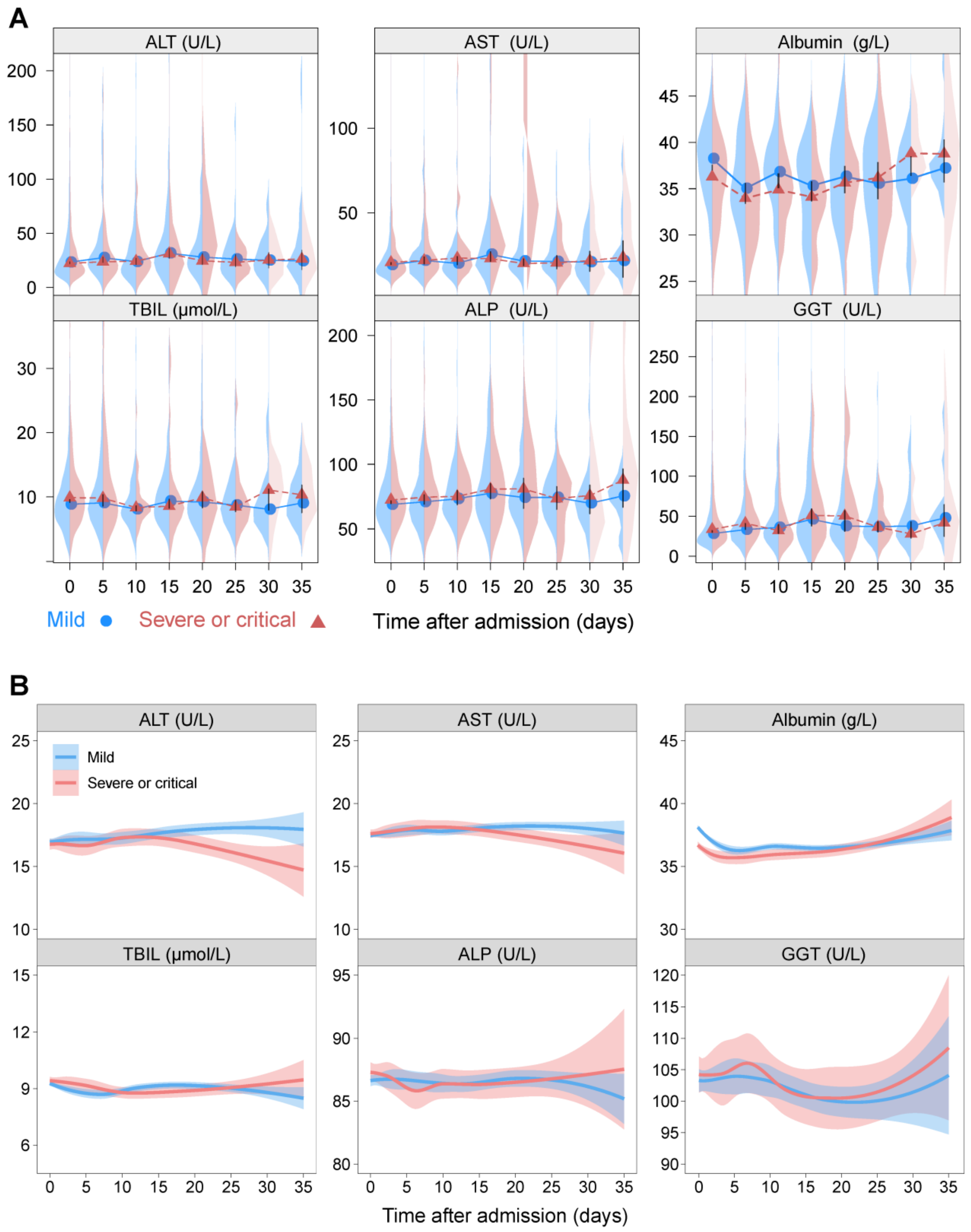

Supplementary Figure 2: liver function variation according to severity of COVID19.

(**A**) Longitudinal back-to-back violin plots showing the variations of ALT, AST, albumin, TBIL, ALP, and GGT during follow-up stratified by severity of COVID-19 on admission. Circles and triangles indicate medians. The black vertical bars have lengths equal to one-half the length of the 95% confidence interval for the difference in medians. When this bar does not touch the circles and triangles, there is a significant difference in medians at the 0.05 level. (**B**) Smooth trajectories of mean values of ALT, AST, albumin,

TBIL, ALP, and GGT by disease severity with 95% confidence band based on locally weighted scatterplot smoothing stratified by severity of COVID-19 on admission.

Abbreviations: ALT, alanine aminotransferase; ALP, alkaline phosphatase; AST, aspartate aminotransferase; COVID-19, coronavirus disease 2019; GGT, gamma-glutamyltransferase; TBIL, total bilirubin.

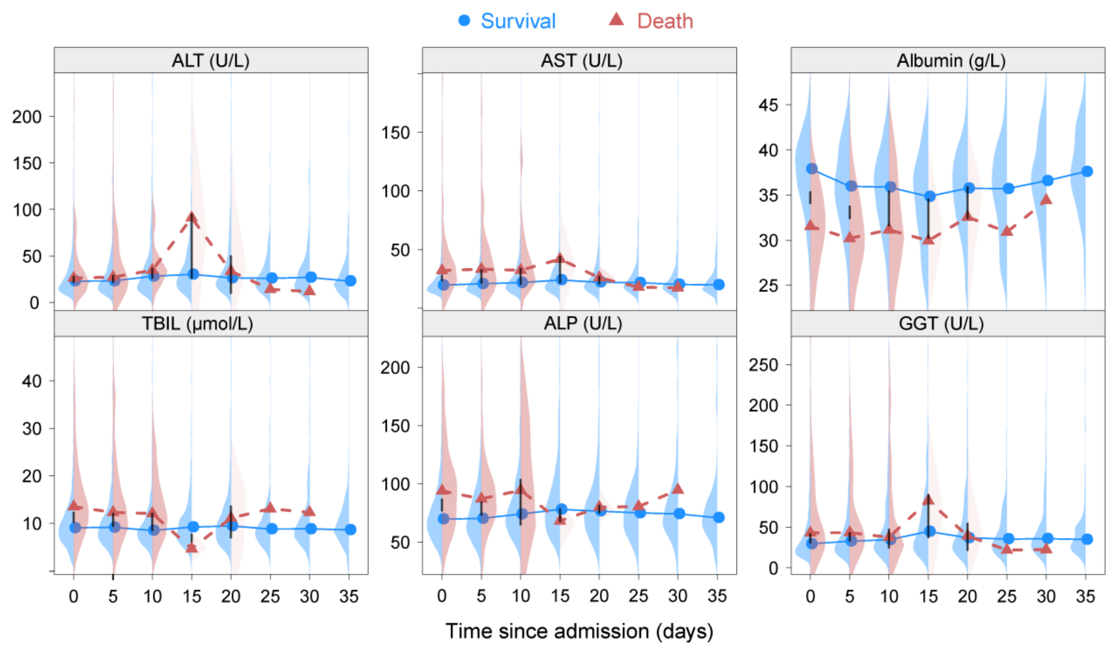

Supplementary Figure 3: liver function variation according to death or survival

Longitudinal back-to-back violin plots showing the variations of ALT, AST, albumin, TBIL, ALP, and GGT during follow-up stratified by death or not. Circles and triangles indicate medians. The black vertical bars have lengths equal to one-half the length of the 95% confidence interval for the difference in medians. When this bar does not touch the circles and triangles, there is a significant difference in medians at the 0.05 level.

Abbreviations: ALT, alanine aminotransferase; ALP, alkaline phosphatase; AST, aspartate aminotransferase; GGT, gamma-glutamyltransferase; TBIL, total bilirubin.

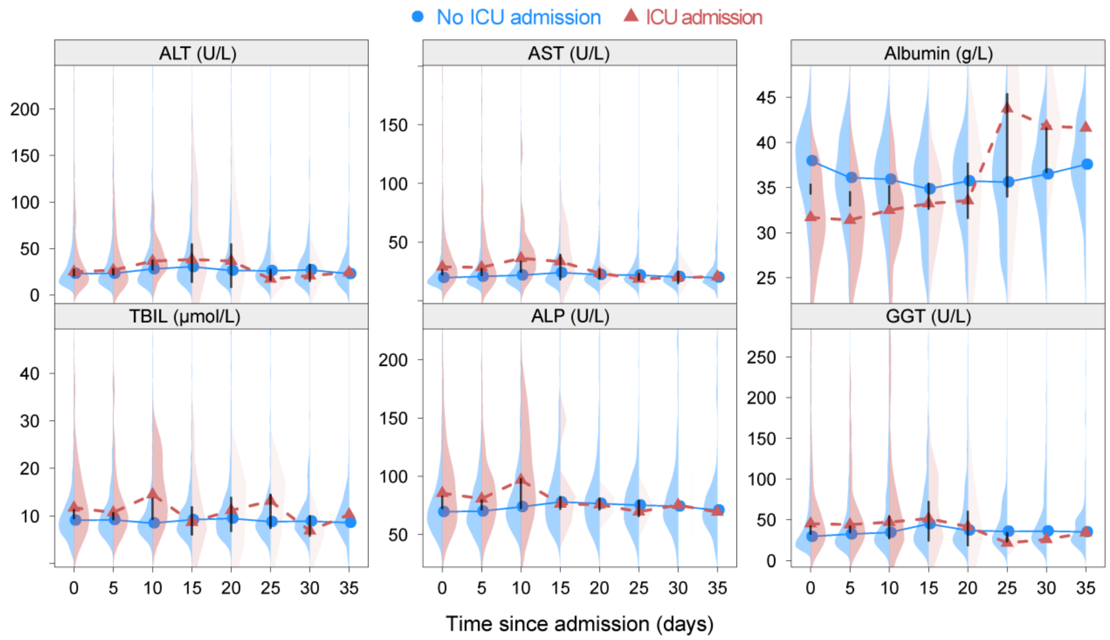

Supplementary Figure 4: liver function variation according to ICU admission or not

Longitudinal back-to-back violin plots showing the variations of ALT, AST, albumin, TBIL, ALP, and GGT during follow-up stratified by ICU admission or not. Circles and triangles indicate medians. The black vertical bars have lengths equal to one-half the length of the 95% confidence interval for the difference in medians. When this bar does not touch the circles and triangles, there is a significant difference in medians at the 0.05 level.

Abbreviations: ALT, alanine aminotransferase; ALP, alkaline phosphatase; AST, aspartate aminotransferase; GGT, gamma-glutamyltransferase; ICU, intensive care unit; TBIL, total bilirubin.

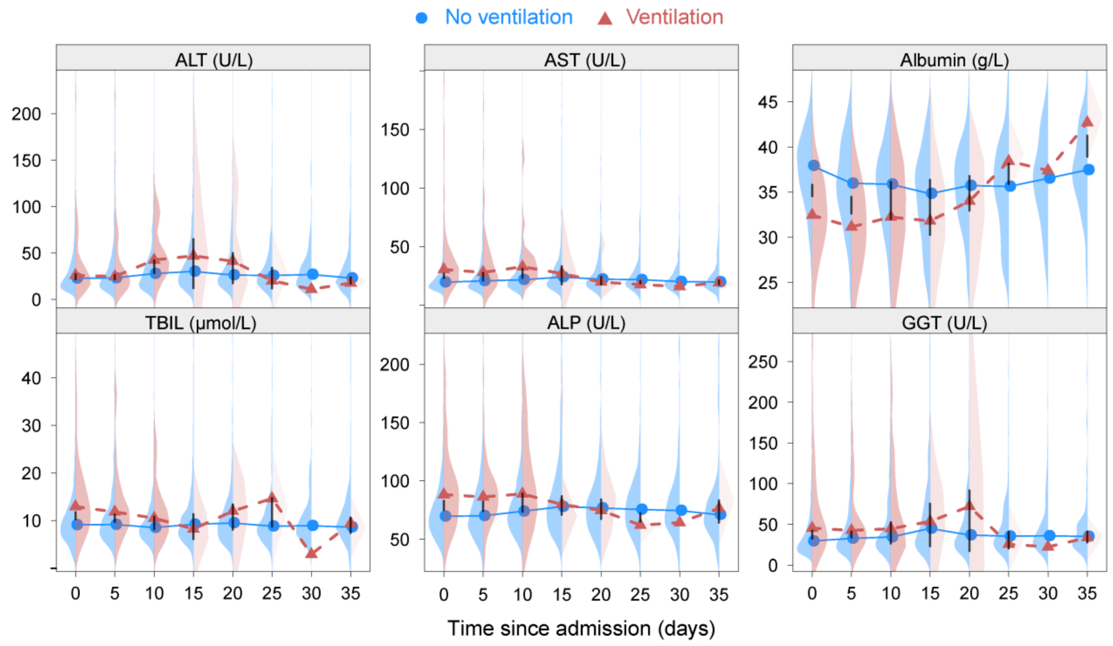

Supplementary Figure 5: liver function variation according to mechanical ventilation or not

Longitudinal back-to-back violin plots showing the variations of ALT, AST, albumin, TBIL, ALP, and GGT during follow-up stratified by mechanical ventilation or not. Circles and triangles indicate medians. The black vertical bars have lengths equal to one-half the length of the 95% confidence interval for the difference in medians. When this bar does not touch the circles and triangles, there is a significant difference in medians at the 0.05 level

Abbreviations: ALT, alanine aminotransferase; ALP, alkaline phosphatase; AST, aspartate aminotransferase; GGT, gamma-glutamyltransferase; TBIL, total bilirubin.

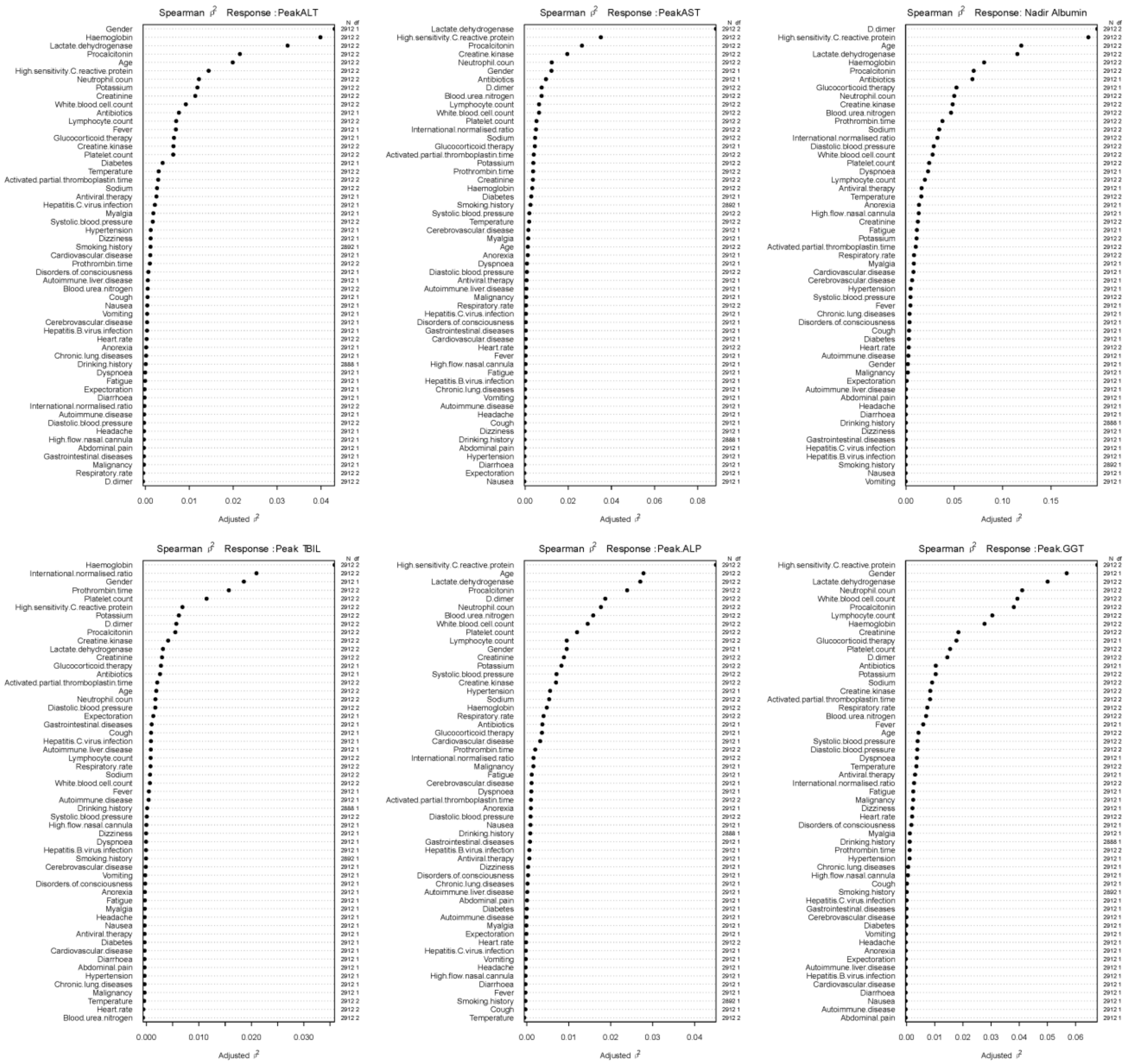

Supplementary Figure 6: Generalized squared rank correlations between baseline parameters and peak (nadir) values of liver function tests during hospitalization. Parameters were allocated in descending order of ρ^2.

Abbreviations: ALT, alanine aminotransferase; ALP, alkaline phosphatase; AST, aspartate aminotransferase; GGT, gamma-glutamyltransferase; TBIL, total bilirubin.

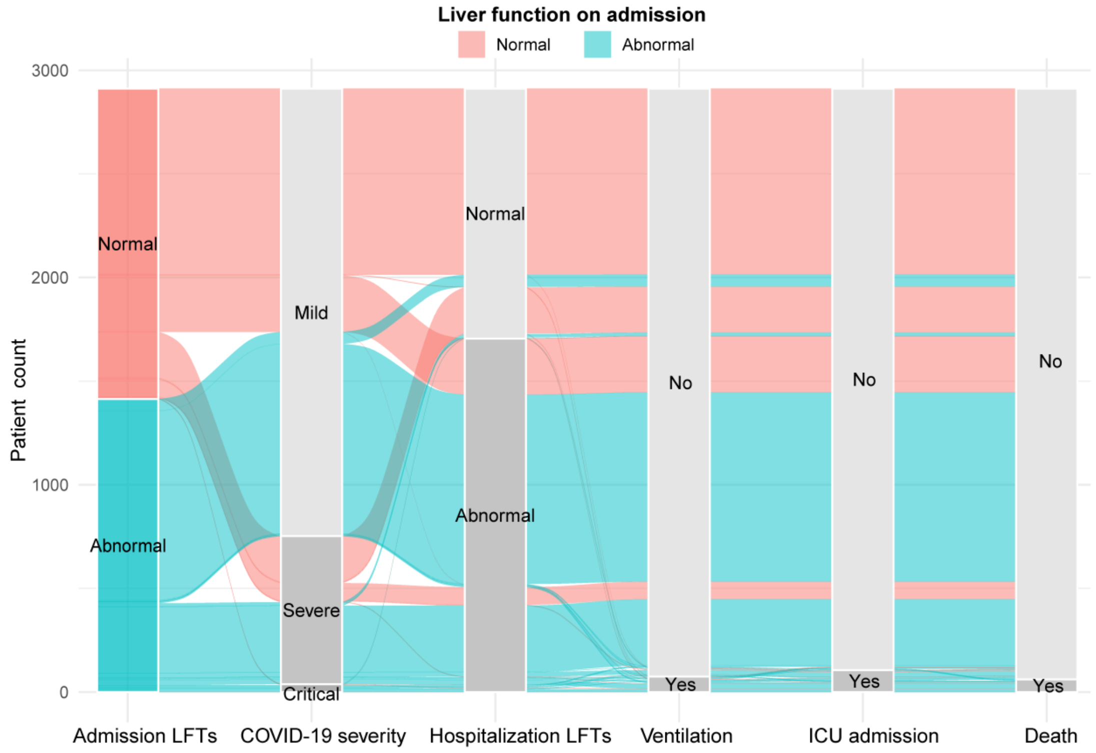

Supplementary Figure 7: The Sankey diagram showing the status flow of all 2912 patients with COVID-19 by the final follow-up.

Abbreviations: COVID-19, coronavirus disease 2019; GGT, gamma-glutamyltransferase; ICU, intensive care unit; LFTs, liver function tests

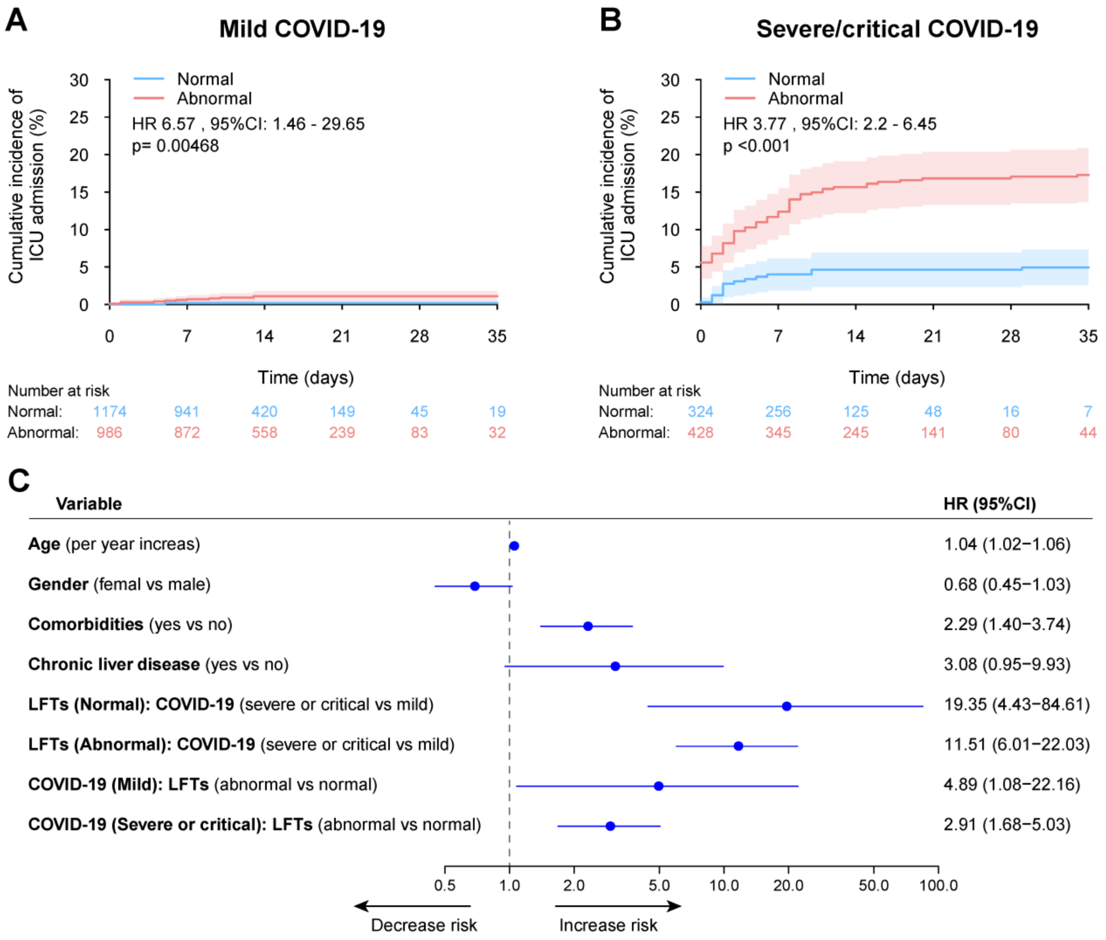

Supplementary Figure 8: Cumulative incidence of ICU admission according to admission abnormal versus normal liver function tests (LFTs) and severity of COVID-19 infection

(A) Cumulative incidence of ICU admission in patients with abnormal versus normal LFTs on admission and mild COVID-19 infection. **(B)** Cumulative incidence of ICU admission in patients with abnormal versus normal LFTs on admission and severe/critical COVID-19 infection. **(C)** Forest plot showing the interaction test of the LFTs (normal vs abnormal) and severity of COVID-19 infection (mild vs severe/critical) on ICU admission after adjustment for potential confounders using the Cox multivariable regression models. $P_{interaction}$=0.482, showing a homogeneous effect of LFTs on ICU admission across the severity of COVID-19 infection.

Note: Comorbidities include hypertension, cardiovascular disease, diabetes, chronic pulmonary diseases, cerebrovascular disease, malignancy and autoimmune disease. Chronic liver diseases include hepatitis B virus infection, hepatitis C virus infection and autoimmune liver disease.

Abbreviation: COVID-19, coronavirus disease 2019; ICU, intensive care unit; LFTs, liver function tests.

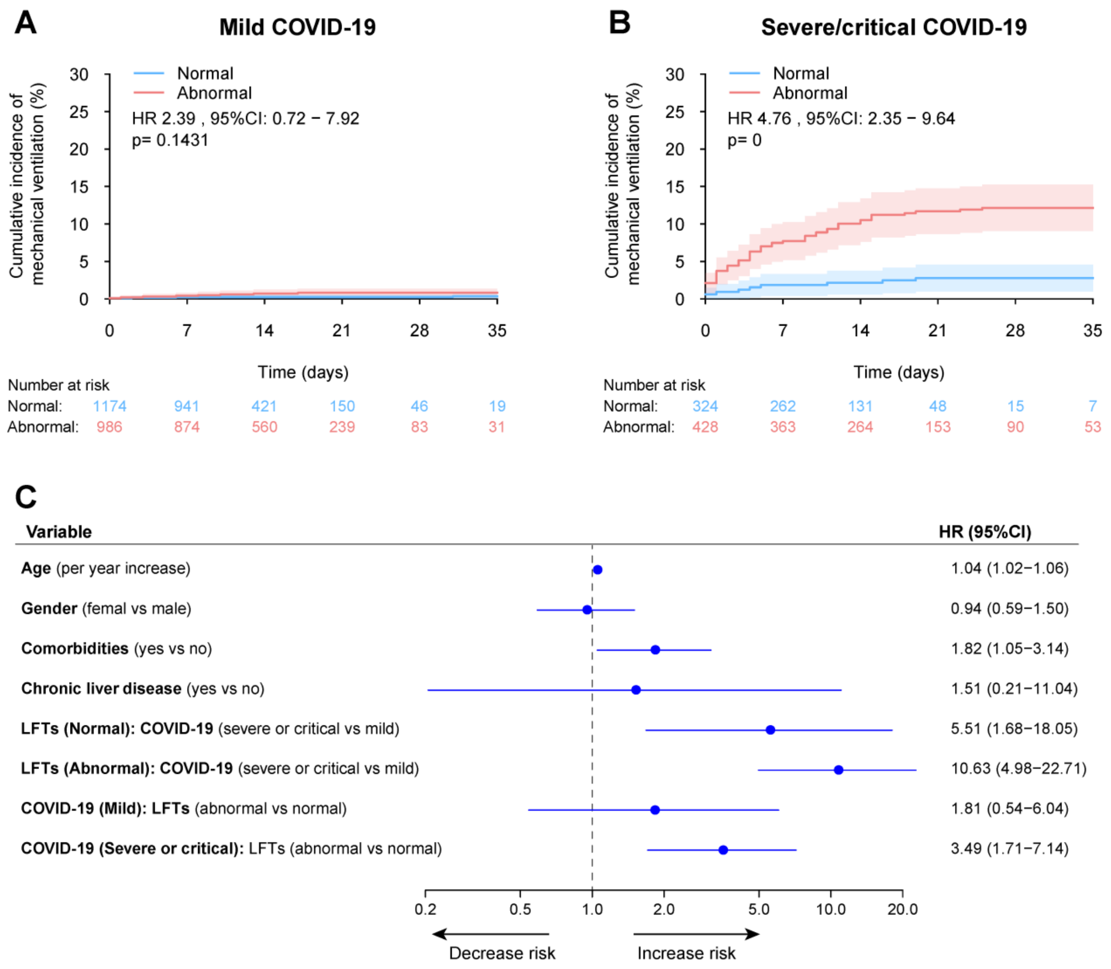

Supplementary Figure 9: Cumulative incidence of mechanical ventilation according to admission abnormal versus normal liver function tests (LFTs) and severity of COVID-19 infection

(A) Cumulative incidence of mechanical ventilation in patients with abnormal versus normal LFTs on admission and mild COVID-19 infection. (B) Cumulative incidence of mechanical ventilation in patients with abnormal versus normal LFTs on admission and severe/critical COVID-19 infection. (C) Forest plot showing the interaction test of the LFTs (normal vs abnormal) and severity of COVID-19 infection (mild vs severe/critical) on mechanical ventilation after adjustment for potential confounders using the Cox multivariable regression models. $P_{interaction}$=0.326, showing a homogeneous effect of LFTs on mechanical ventilation across the severity of COVID-19 infection.

Note: Comorbidities include hypertension, cardiovascular disease, diabetes, chronic pulmonary diseases, cerebrovascular disease, malignancy and autoimmune disease. Chronic liver diseases include hepatitis B virus infection, hepatitis C virus infection and autoimmune liver disease.

Abbreviation: COVID-19, coronavirus disease 2019; LFTs, liver function tests.

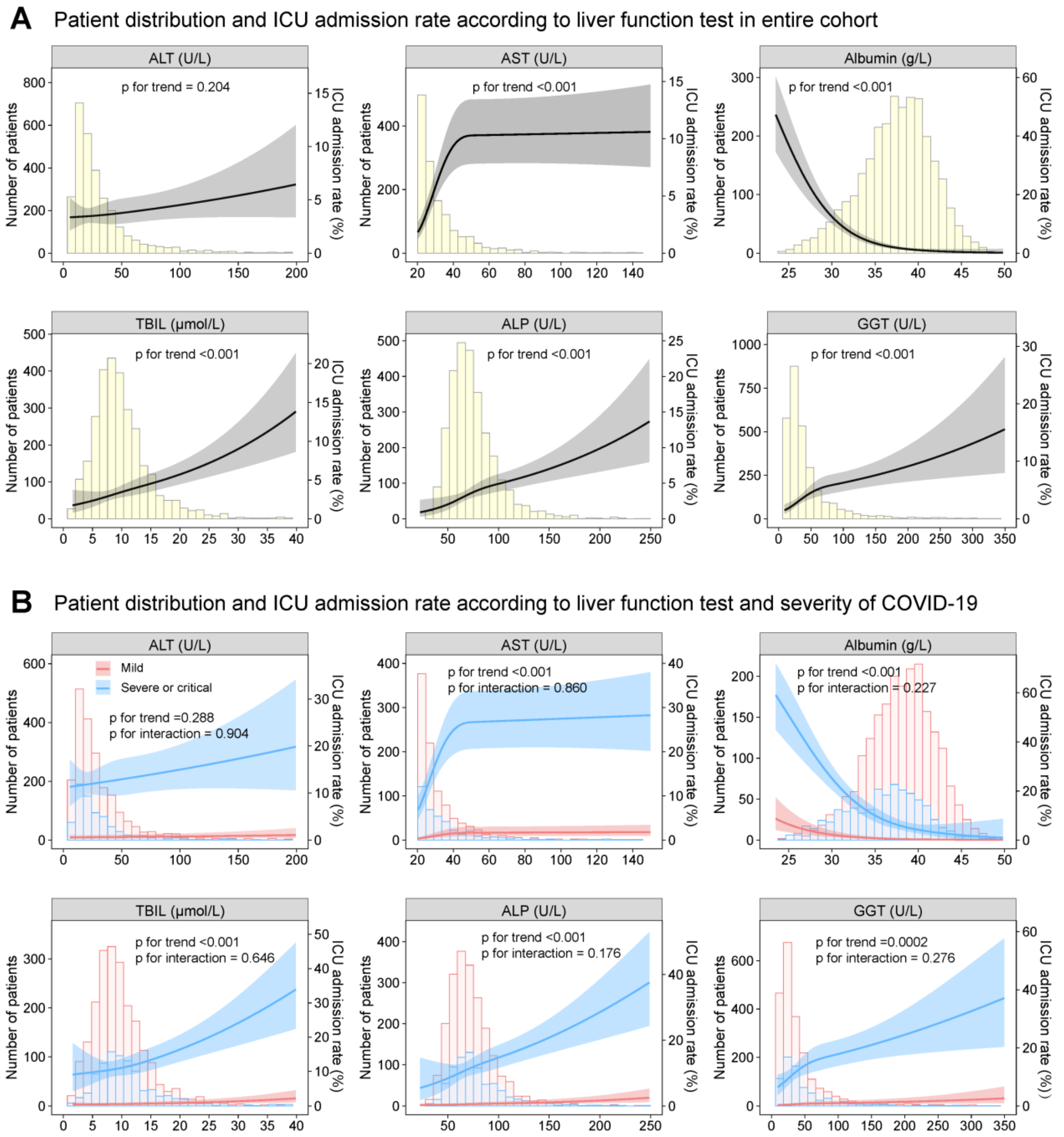

Supplementary Figure 10: Patient distribution and ICU admission rate according to liver function test

Patient distribution and ICU admission rate according to ALT, AST, albumin, TBIL, ALP, and GGT on admission **(A)** in entire cohort **(B)** by severity of COVID-19 infection (mild vs severe/critical). Restricted cubic splines were generated using logistic regression models.

Abbreviation: ALT, alanine aminotransferase; AST, aspartate transaminase; ALP, alkaline phosphatase; COVID-19, coronavirus disease 2019; GGT, gamma-glutamyltransferase; ICU, intensive care unit; TBIL, total bilirubin.

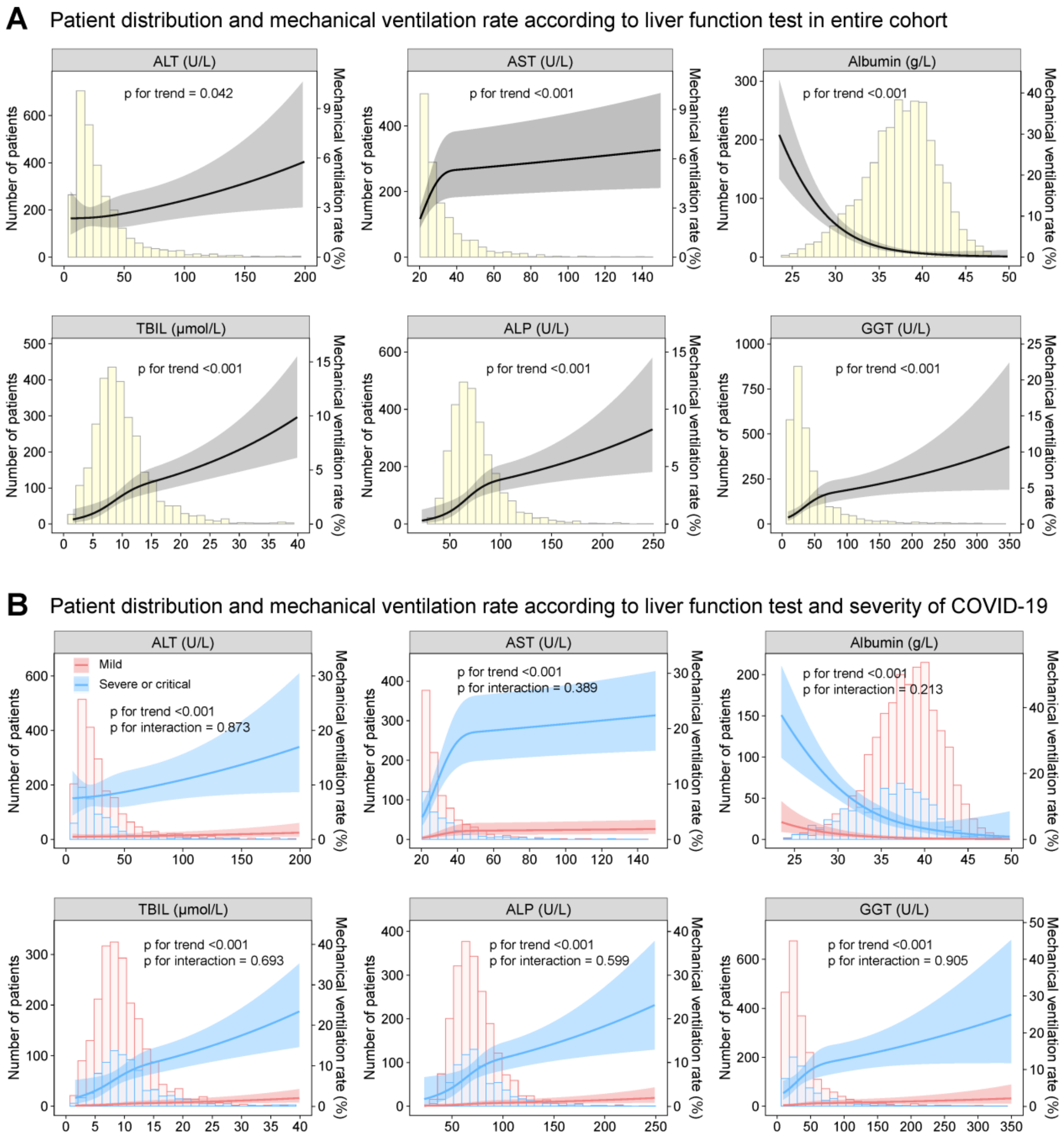

Supplementary Figure 11: Patient distribution and mechanical ventilation rate according to baseline liver function tests

Patient distribution and mechanical ventilation rate according to ALT, AST, albumin, TBIL, ALP, and GGT on admission **(A)** in entire cohort **(B)** by severity of COVID-19 infection (mild vs severe/critical). Restricted cubic splines were generated using logistic regression models.

Abbreviations: ALT, alanine aminotransferase; AST, aspartate transaminase; ALP, alkaline phosphatase; COVID-19, coronavirus disease 2019; GGT, gamma-glutamyltransferase; TBIL, total bilirubin.

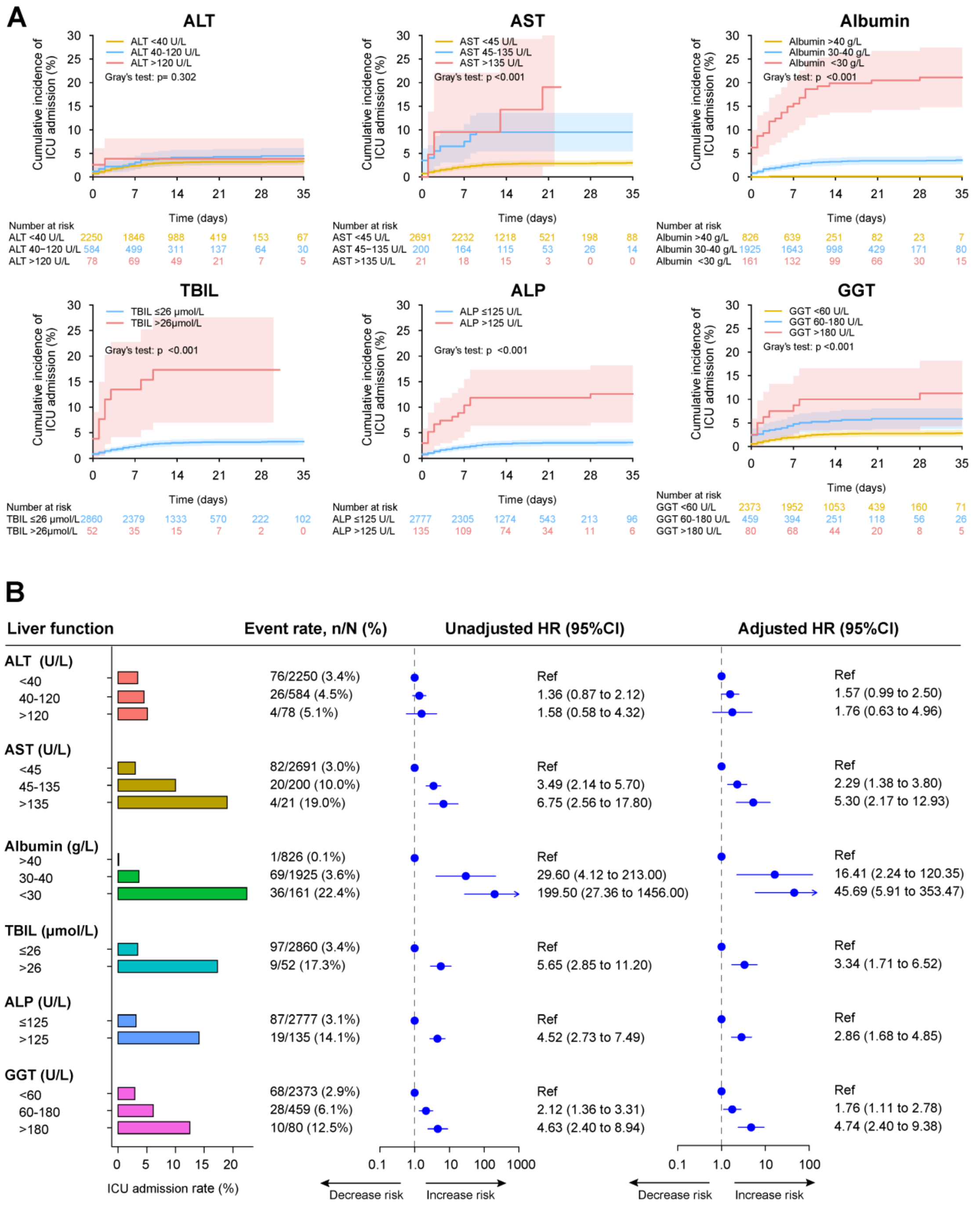

Supplementary Figure 12: ICU admission rate in COVID-19 patients with different level of liver function tests

(A) Cumulative incidence of ICU admission during hospitalization in patients with different level of liver function test on admission. **(B)** ICU admission rate in patients with different level of liver function test on admission, the unadjusted adjusted effect of liver function test at different level on the ICU admission during hospitalization.

Note: Adjusted HRs are derived from multivariate Cox regression models, adjusted for age, gender,

comorbidities (hypertension, cardiovascular disease, diabetes, chronic pulmonary diseases, cerebrovascular disease, malignancy and autoimmune disease) and chronic liver diseases (hepatitis B virus infection, hepatitis C virus infection and autoimmune liver disease) .

Abbreviations: ALT, alanine aminotransferase; ALP, alkaline phosphatase; AST, aspartate aminotransferase; CI, confidence interval; GGT, gamma-glutamyltransferase; ICU, intensive care unit; HR, hazard ratio; TBIL, total bilirubin.

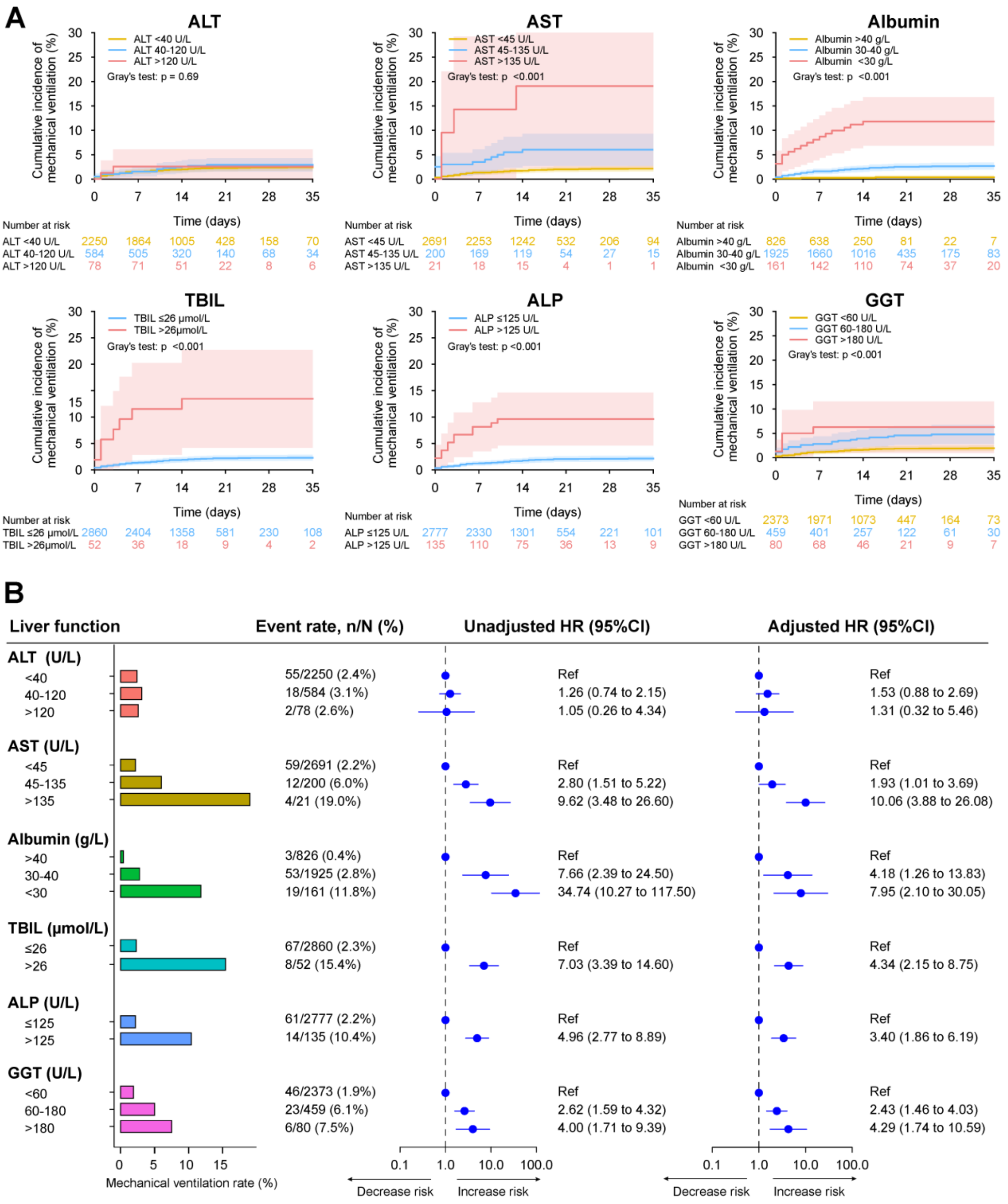

Supplementary Figure 13: Mechanical ventilation rate in patients with different level of liver function tests

(A) Cumulative incidence of mechanical ventilation during hospitalization in patients with different level of liver function test on admission. **(B)** Mechanical ventilation rate in patients with different level of liver function test on admission, the unadjusted adjusted effect of liver function test at different level on the mechanical ventilation during hospitalization.

Note: Adjusted HRs are derived from multivariate Cox regression models, adjusted for age, gender, comorbidities (hypertension, cardiovascular disease, diabetes, chronic pulmonary diseases,

cerebrovascular disease, malignancy and autoimmune disease) and chronic liver diseases (hepatitis B virus infection, hepatitis C virus infection, autoimmune liver disease) .

Abbreviations: ALT, alanine aminotransferase; ALP, alkaline phosphatase; AST, aspartate aminotransferase; CI, confidence interval; GGT, gamma-glutamyltransferase; HR, hazard ratio; TBIL, total bilirubin.

Variable	Level	Liver function tests: Normal (n=1130)	Liver function tests: Abnormal (n=368)	Univariable analysis: Units	Odds ratios	p value
Age	mean (sd)	56.1 (14)	58.1 (14.7)		1.01 (1.00−1.02)	0.018
Gender	Female	680 (60.2)	217 (59.0)	Female vs Male	0.95 (0.75−1.21)	0.726
Smoking.history	yes	84 (7.4)	26 (7.1)	yes vs no	0.95 (0.60−1.49)	0.904
Drinking.history	yes	55 (4.9)	15 (4.1)	yes vs no	0.83 (0.46−1.49)	0.630
Severity of COVID-19	Severe or critical	228 (20.2)	96 (26.1)	Severe or critical vs Mild	1.40 (1.06−1.84)	0.020
Comorbidities	yes	457 (40.4)	159 (43.2)	yes vs no	1.12 (0.88−1.42)	0.382
Hypertension	yes	341 (30.2)	120 (32.6)	yes vs no	1.12 (0.87−1.44)	0.416
Cardiovascular.disease	yes	75 (6.6)	32 (8.7)	yes vs no	1.34 (0.87−2.06)	0.224
Diabetes	yes	158 (14.0)	47 (12.8)	yes vs no	0.90 (0.64−1.28)	0.617
Chronic.lung.diseases	yes	45 (4.0)	18 (4.9)	yes vs no	1.24 (0.71−2.17)	0.545
Cerebrovascular.disease	yes	36 (3.2)	19 (5.2)	yes vs no	1.65 (0.94−2.92)	0.111
Malignancy	yes	19 (1.7)	6 (1.6)	yes vs no	0.97 (0.38−2.45)	1.000
Gastrointestinal.diseases	yes	19 (1.7)	3 (0.8)	yes vs no	0.48 (0.14−1.63)	0.342
Chronic.liver.disease	yes	25 (2.2)	6 (1.6)	yes vs no	0.73 (0.30−1.80)	0.638
Hepatitis.B.virus.infection	yes	20 (1.8)	6 (1.6)	yes vs no	0.92 (0.37−2.31)	1.000
Fever	yes	778 (68.8)	251 (68.2)	yes vs no	0.97 (0.75−1.25)	0.868
Cough	yes	752 (66.5)	246 (66.8)	yes vs no	1.01 (0.79−1.30)	0.966
Expectoration	yes	148 (13.1)	38 (10.3)	yes vs no	0.76 (0.52−1.11)	0.190
Dyspnoea	yes	472 (41.8)	179 (48.6)	yes vs no	1.32 (1.04−1.67)	0.025
Fatigue	yes	515 (45.6)	182 (49.5)	yes vs no	1.17 (0.92−1.48)	0.216
Myalgia	yes	259 (22.9)	98 (26.6)	yes vs no	1.22 (0.93−1.60)	0.167
Anorexia	yes	169 (15.0)	59 (16.0)	yes vs no	1.09 (0.79−1.50)	0.677
Nausea	yes	29 (2.6)	9 (2.4)	yes vs no	0.95 (0.45−2.03)	1.000
Vomiting	yes	24 (2.1)	7 (1.9)	yes vs no	0.89 (0.38−2.09)	0.961
Abdominal.pain	yes	14 (1.2)	3 (0.8)	yes vs no	0.66 (0.19−2.29)	0.702
Diarrhoea	yes	45 (4.0)	11 (3.0)	yes vs no	0.74 (0.38−1.45)	0.475
Headache	yes	21 (1.9)	9 (2.4)	yes vs no	1.32 (0.60−2.92)	0.628
Dizziness	yes	21 (1.9)	3 (0.8)	yes vs no	0.43 (0.13−1.46)	0.252
Disorders.of.consciousness	yes	2 (0.2)	3 (0.8)	yes vs no	4.64 (0.77−27.85)	0.186
Temperature	mean (sd)	36.5 (0.4)	36.5 (0.4)		1.34 (1.00−1.79)	0.050
Systolic.blood.pressure	mean (sd)	130.8 (17)	128.7 (15.1)		0.99 (0.99−1.00)	0.039
Diastolic.blood.pressure	mean (sd)	81.7 (11.6)	80.7 (10.4)		0.99 (0.98−1.00)	0.116
Heart.rate	mean (sd)	87.3 (13.4)	86.8 (13.2)		1.00 (0.99−1.01)	0.499
Respiratory.rate	mean (sd)	20.2 (2.9)	20 (1.9)		0.97 (0.92−1.03)	0.332
White.blood.cell.count	mean (sd)	5.8 (2.1)	6 (2.1)		1.04 (0.99−1.10)	0.105
Neutrophil.coun	mean (sd)	3.5 (1.5)	3.8 (1.9)		1.11 (1.04−1.19)	<0.01
Lymphocyte.count	mean (sd)	0.6 (0.9)	0.8 (1.9)		1.14 (1.04−1.25)	<0.01
Haemoglobin	mean (sd)	125.2 (16.6)	124.2 (14.7)		1.00 (0.99−1.00)	0.316
Platelet.count	mean (sd)	226.2 (70.7)	222.4 (67.7)		1.00 (1.00−1.00)	0.370
Creatinine	mean (sd)	66.3 (34.2)	70.9 (76.7)		1.00 (1.00−1.00)	0.113
Blood.urea.nitrogen	mean (sd)	5.3 (17.4)	5 (3.4)		1.00 (0.99−1.01)	0.726
Potassium	mean (sd)	4.3 (0.4)	4.5 (2.9)		1.12 (0.95−1.32)	0.011
Sodium	mean (sd)	141.7 (4.7)	141.6 (2.7)		0.99 (0.97−1.02)	0.608
D-dimer	mean (sd)	0.7 (2.8)	0.7 (1)		1.00 (0.95−1.05)	0.926
Prothrombin.time	mean (sd)	11 (5.3)	10.5 (5.1)		0.98 (0.96−1.00)	0.125
Antiviral.therapy	yes	443 (39.2)	151 (41.0)	yes vs no	1.08 (0.85−1.37)	0.574
Abidor	yes	414 (36.6)	142 (38.6)	yes vs no	1.09 (0.85−1.38)	0.542
Ribavirin	yes	16 (1.4)	6 (1.6)	yes vs no	1.15 (0.45−2.97)	0.962
Oseltamivir	yes	47 (4.2)	16 (4.3)	yes vs no	1.05 (0.59−1.87)	0.994
Interferon	yes	94 (8.3)	19 (5.2)	yes vs no	0.60 (0.36−1.00)	0.060
Antibiotics	yes	262 (23.2)	110 (29.9)	yes vs no	1.41 (1.09−1.84)	0.012
Quinolones	yes	180 (15.9)	85 (23.1)	yes vs no	1.59 (1.19−2.12)	<0.01
Cephalosporins	yes	5 (0.4)	9 (2.4)	yes vs no	5.64 (1.88−16.94)	<0.01
Macrolides	yes	10 (0.9)	2 (0.5)	yes vs no	0.61 (0.13−2.81)	0.763
Traditional.Chinese.medicine	yes	1024 (90.6)	338 (91.8)	yes vs no	1.17 (0.76−1.78)	0.543
Immunoglobin	yes	25 (2.2)	10 (2.7)	yes vs no	1.23 (0.59−2.60)	0.720
Glucocorticoid.therapy	yes	78 (6.9)	38 (10.3)	yes vs no	1.55 (1.03−2.33)	0.043
High.flow.nasal.cannula	yes	636 (56.3)	212 (57.6)	yes vs no	1.06 (0.83−1.34)	0.700
Mechanical ventilation	yes	6 (0.5)	7 (1.9)	yes vs no	3.63 (1.21−10.88)	0.032

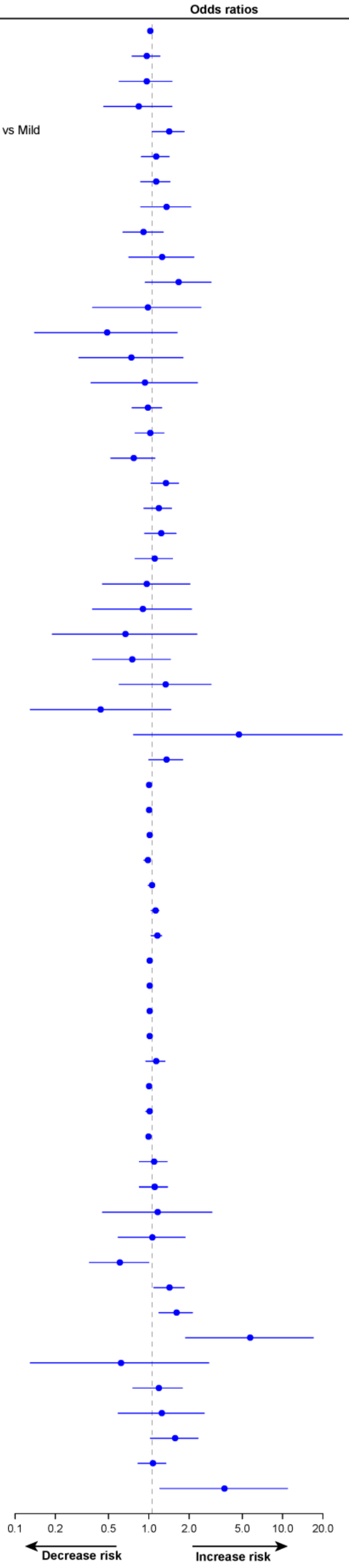

Supplementary Figure 14: Univariable analysis of factors associated with de novo abnormal versus normal liver function tests (LFTs) during hospitalization.

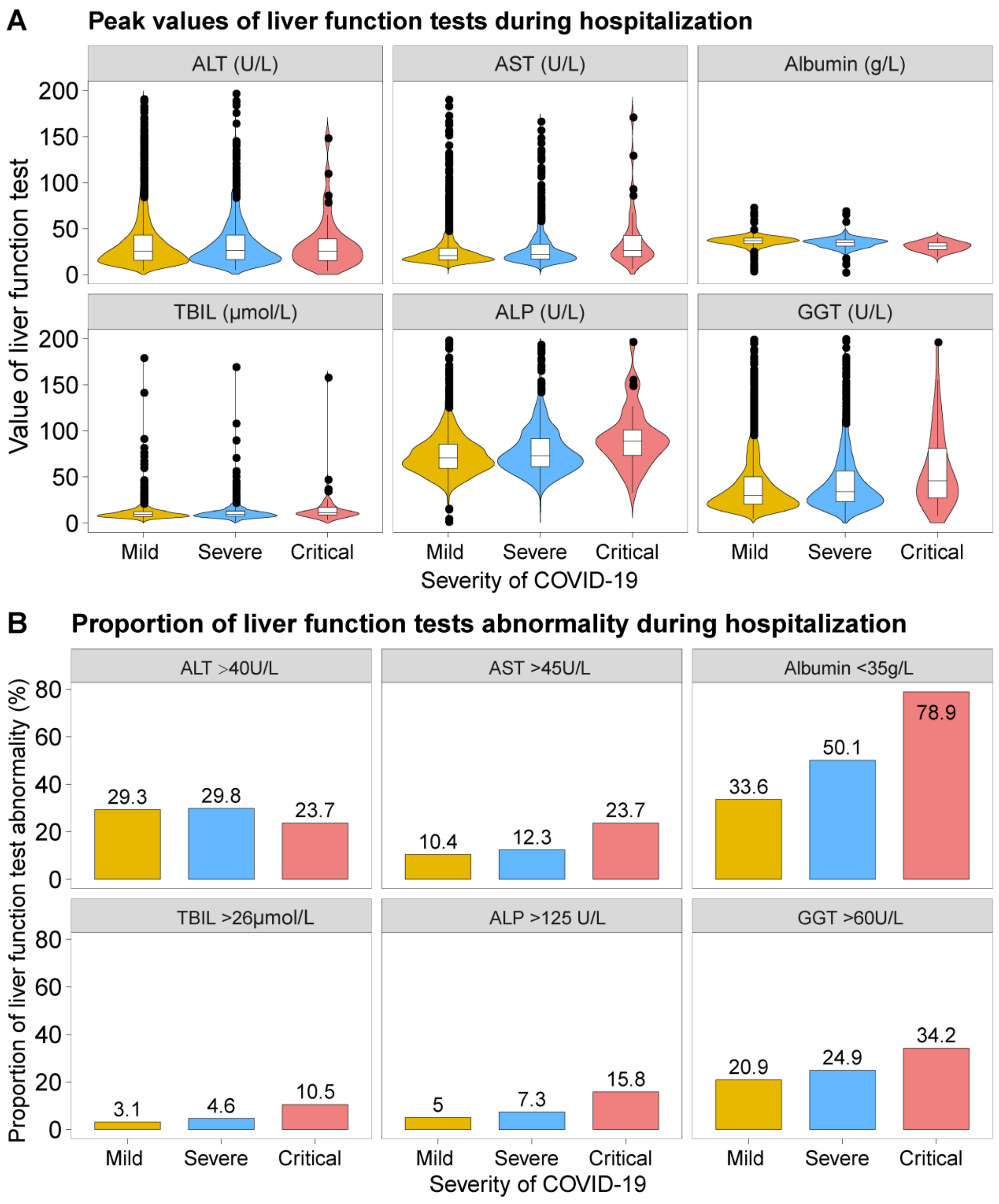

Supplementary Figure 15: Peak (nadir) values of liver function tests during hospitalization

(A) Violin and box plots showing the median values of peak ALT, peak AST, nadir albumin, peak TBIL, peak ALP, and peak GGT during hospitalization by severity of the COVID-19 disease. (B) Bar plots showing the proportion of abnormal peak ALT, peak AST, nadir albumin, peak TBIL, peak ALP, and peak GGT during hospitalization by severity of the COVID-19 disease.

Abbreviation: ALT, alanine aminotransferase; AST, aspartate transaminase; ALP, alkaline phosphatase; COVID-19, coronavirus disease 2019; GGT, gamma-glutamyltransferase; TBIL, total bilirubin.

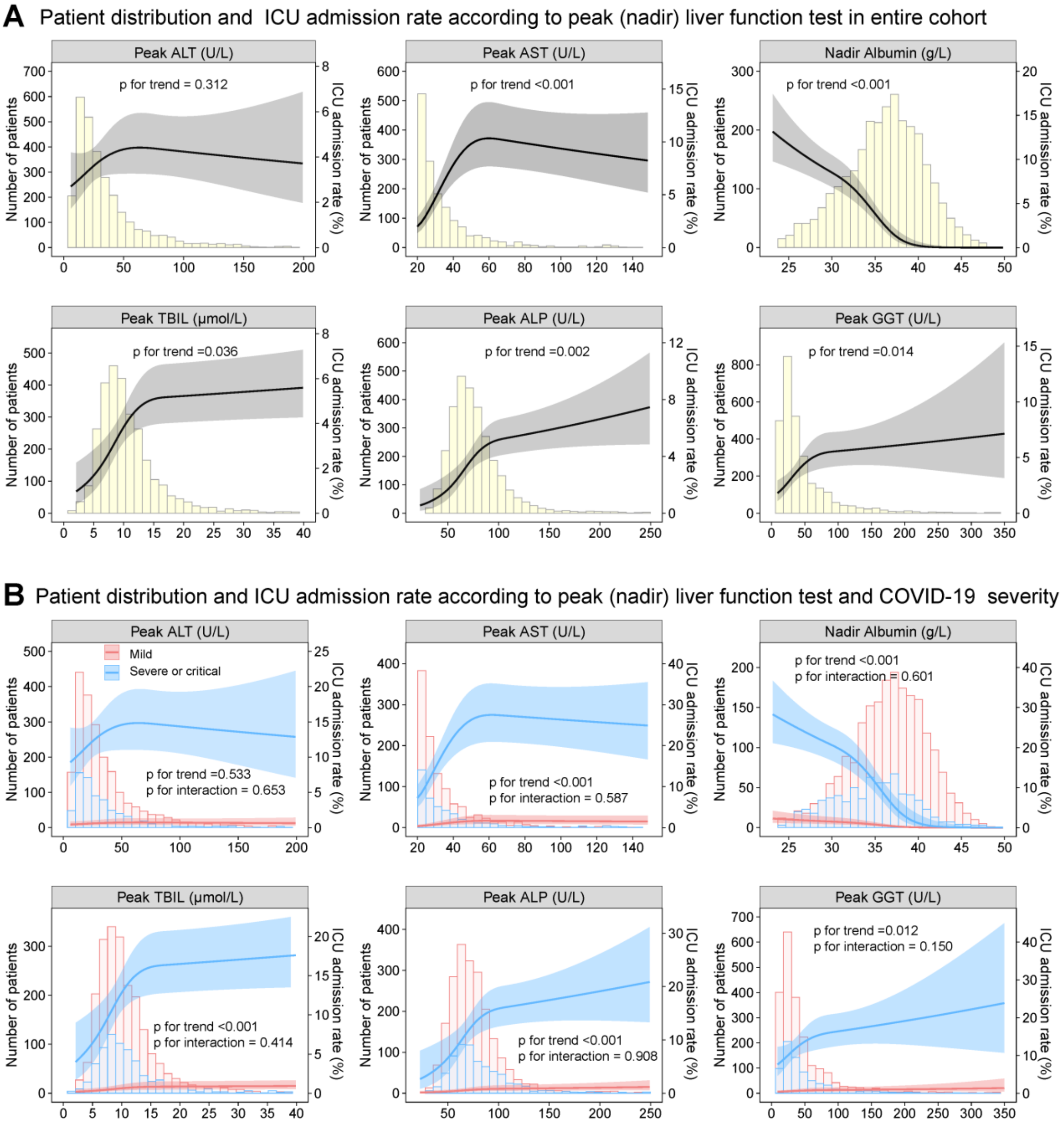

Supplementary Figure 16: Patient distribution and ICU admission rates according to peak (nadir) liver function test in entire cohort

Patient distribution and ICU admission rate according to peak ALT, peak AST, nadir albumin, peak TBIL, peak ALP, and peak GGT during hospitalization **(A)** in entire cohort **(B)** by severity of COVID19 infection (mild vs severe/critical). Restricted cubic splines were generated using logistic regression models.

Abbreviation: ALT, alanine aminotransferase; AST, aspartate transaminase; ALP, alkaline phosphatase; ICU, intensive care unit; GGT, gamma-glutamyltransferase; TBIL, total bilirubin abnormal.

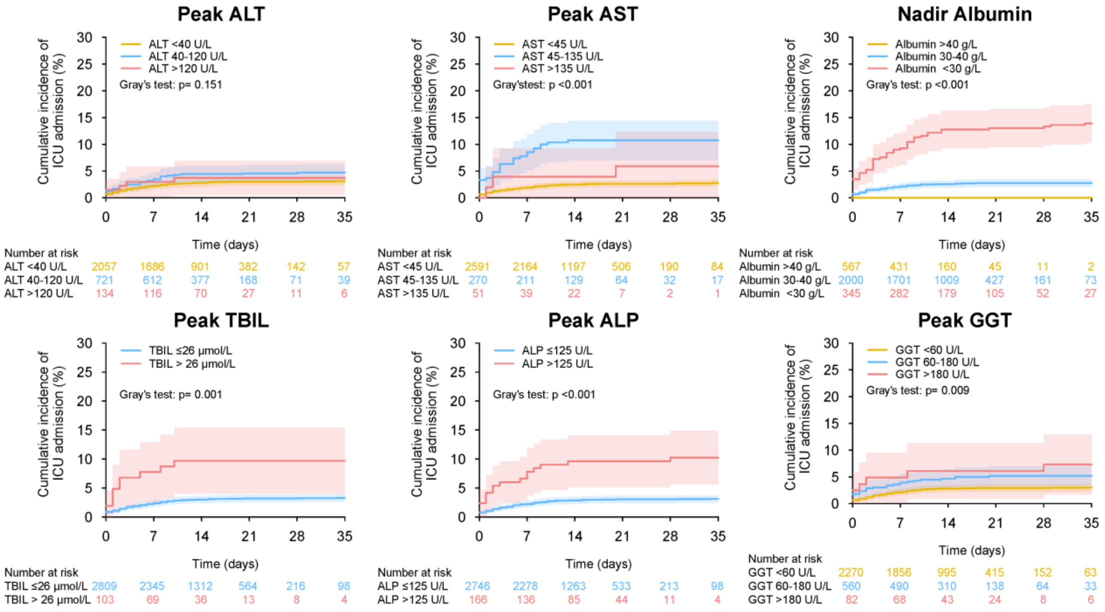

Supplementary Figure 17: ICU admission rates during hospitalization in patients with different level of peak (nadir) liver function test in entire cohort

Cumulative incidence of ICU admission rate during hospitalization in patients with different level of peak ALT, peak AST, nadir albumin, peak TBIL, peak ALP, and peak GGT during hospitalization

Abbreviation: ALT, alanine aminotransferase; AST, aspartate transaminase; ALP, alkaline phosphatase; GGT, gamma-glutamyltransferase; TBIL, total bilirubin abnormal.

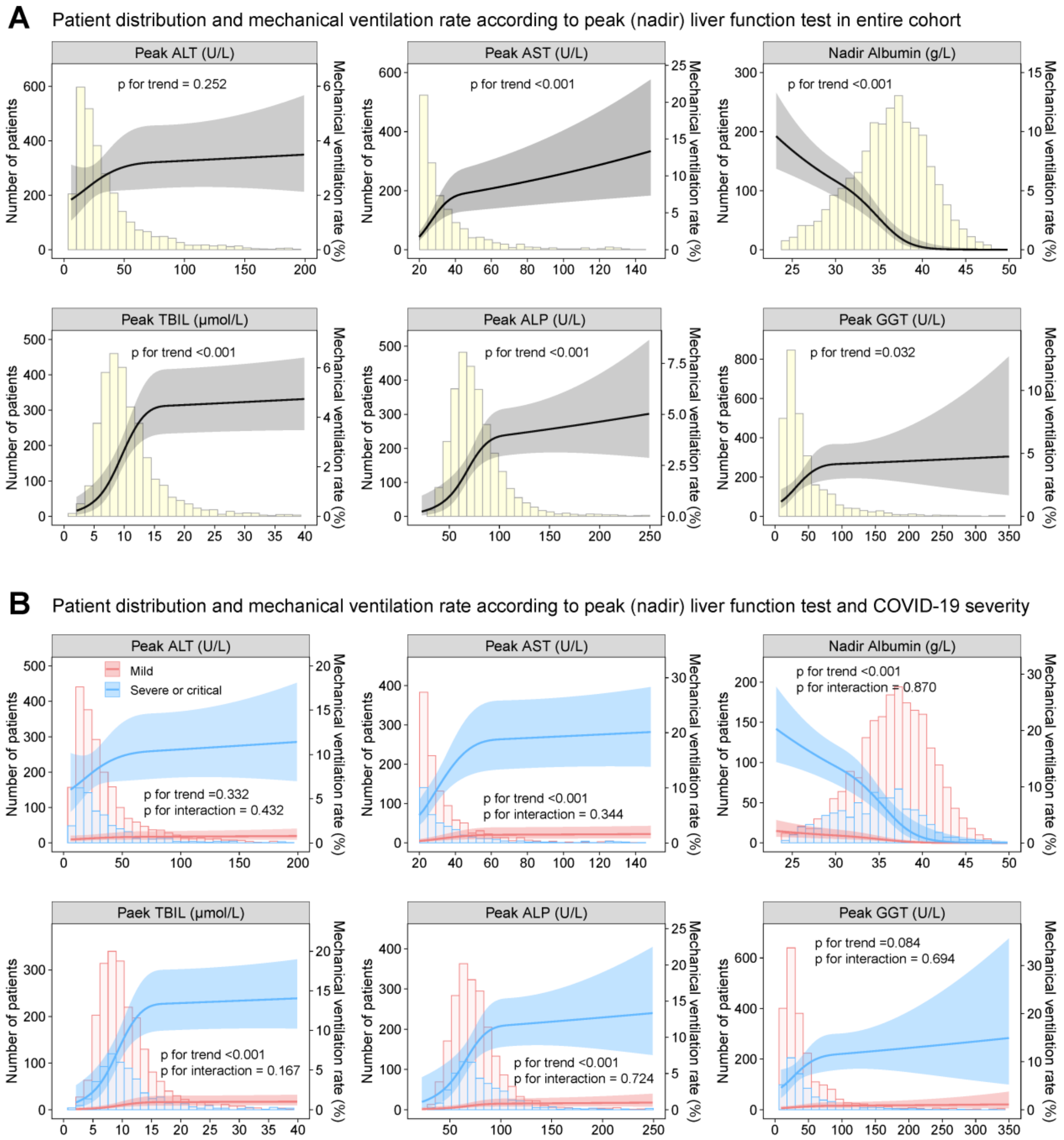

Supplementary Figure 18: Patient distribution and mechanical ventilation rate according to peak (nadir) liver function test in entire cohort

Patient distribution and mechanical ventilation rate according to peak ALT, peak AST, nadir albumin, peak TBIL, peak ALP, and peak GGT during hospitalization **(A)** in entire cohort **(B)** by severity of COVID19 infection (mild vs severe/critical). Restricted cubic splines were generated using logistic regression models.

Abbreviation: ALT, alanine aminotransferase; AST, aspartate transaminase; ALP, alkaline phosphatase; GGT, gamma-glutamyltransferase; TBIL, total bilirubin abnormal.

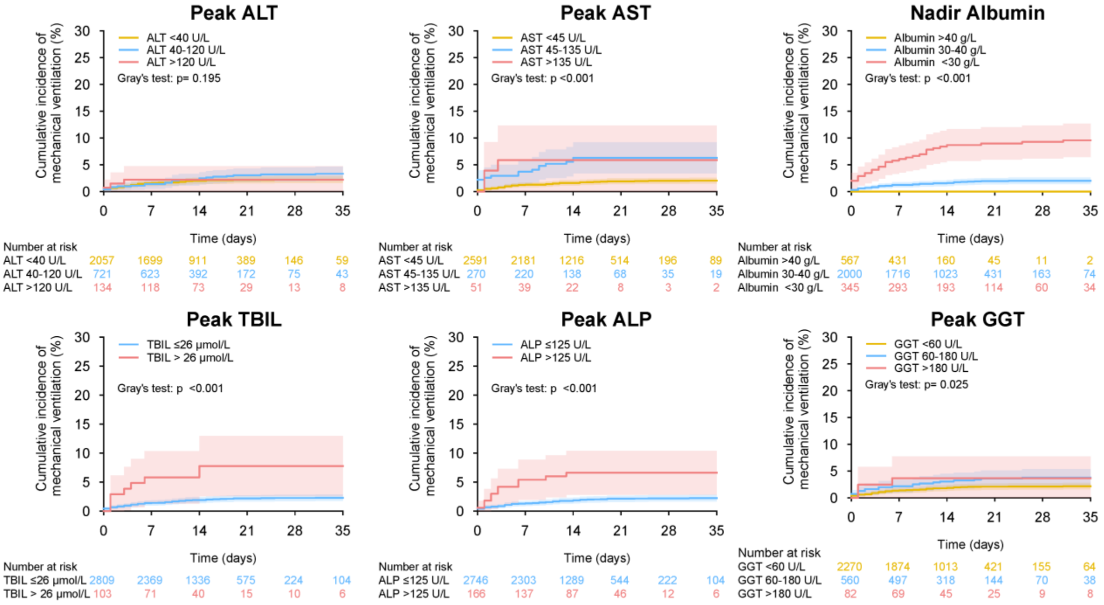

Supplementary Figure 19: Mechanical ventilation rate in patients with different level of peak (nadir) liver function test in entire cohort

Cumulative incidence of mechanical ventilation during hospitalization in patients with different level of peak ALT, peak AST, nadir albumin, peak TBIL, peak ALP, and peak GGT during hospitalization.

Abbreviation: ALT, alanine aminotransferase; AST, aspartate transaminase; ALP, alkaline phosphatase; GGT, gamma-glutamyltransferase; TBIL, total bilirubin abnormal.

Supplementary Table 1. Baseline characteristics of study patients according to the severity of COVID-19 infection on admission

Variable	Total (n=2912)	Mild (n=2160)	Severe (n=714)	Critical (n=38)	P value
Age, years	58.4 ± 14.4	56.4 ± 14.3	64.1 ± 12.9	66.7 ± 15.2	<0.001
Female gender, n (%)	1,512 (51.9%)	1164 (53.9%)	337 (47.2%)	11 (28.9%)	<0.001
Smoking history, n (%)	217 (7.5%)	167 (7.7%)	49 (6.9%)	1 (2.6%)	0.390
Drinking history, n (%)	130 (4.5%)	100 (4.6%)	30 (4.2%)	0 (0.0%)	0.362
**Comorbidities on admission**					
All comorbidities, n (%)	1,236 (42.4%)	803 (37.2%)	409 (57.3%)	24 (63.2%)	<0.001
Hypertension, n (%)	910 (31.2%)	575 (26.6%)	320 (44.8%)	15 (39.5%)	<0.001
Cardiovascular disease, n (%)	219 (7.5%)	126 (5.8%)	88 (12.3%)	5 (13.2%)	<0.001
Diabetes, n (%)	392 (13.5%)	246 (11.4%)	135 (18.9%)	11 (28.9%)	<0.001
Chronic lung diseases, n (%)	141 (4.8%)	82 (3.8%)	54 (7.6%)	5 (13.2%)	<0.001
Cerebrovascular disease, n (%)	125 (4.3%)	64 (3.0%)	55 (7.7%)	6 (15.8%)	<0.001
Malignancy, n (%)	63 (2.2%)	44 (2.0%)	16 (2.2%)	3 (7.9%)	0.048
Autoimmune disease, n (%)	20 (0.7%)	13 (0.6%)	7 (1.0%)	0 (0.0%)	0.498
**Chronic liver diseases, n (%)**	68 (2.3%)	59 (2.7%)	9 (1.3%)	0 (0.0%)	0.049
Hepatitis B virus infection, n (%)	58 (2.0%)	50 (2.3%)	8 (1.1%)	0 (0.0%)	0.095
Hepatitis C virus infection, n (%)	8 (0.3%)	7 (0.3%)	1 (0.1%)	0 (0.0%)	0.681
Autoimmune liver disease, n (%)	2 (0.1%)	2 (0.1%)	0 (0.0%)	0 (0.0%)	0.706
**Clinical characteristics on admission**					
Fever, n (%)	2,057 (70.6%)	1504 (69.6%)	528 (73.9%)	25 (65.8%)	0.072
Cough, n (%)	2,001 (68.7%)	1465 (67.8%)	507 (71.0%)	29 (76.3%)	0.168
Expectoration, n (%)	420 (14.4%)	293 (13.6%)	114 (16.0%)	13 (34.2%)	<0.001
Dyspnoea, n (%)	1,394 (47.9%)	968 (44.8%)	398 (55.7%)	28 (73.7%)	<0.001
Fatigue, n (%)	1,461 (50.2%)	1047 (48.5%)	385 (53.9%)	29 (76.3%)	<0.001
Myalgia, n (%)	774 (26.6%)	583 (27.0%)	177 (24.8%)	14 (36.8%)	0.182
Anorexia, n (%)	523 (18.0%)	383 (17.7%)	125 (17.5%)	15 (39.5%)	<0.001
Nausea, n (%)	63 (2.2%)	47 (2.2%)	11 (1.5%)	5 (13.2%)	<0.001
Vomiting, n (%)	47 (1.6%)	34 (1.6%)	12 (1.7%)	1 (2.6%)	0.865
Abdominal pain, n (%)	31 (1.1%)	19 (0.9%)	12 (1.7%)	0 (0.0%)	0.159
Diarrhoea, n (%)	126 (4.3%)	96 (4.4%)	27 (3.8%)	3 (7.9%)	0.416
Headache, n (%)	54 (1.9%)	40 (1.9%)	12 (1.7%)	2 (5.3%)	0.280
Dizziness, n (%)	36 (1.2%)	28 (1.3%)	7 (1.0%)	1 (2.6%)	0.591
Disorders of consciousness, n (%)	19 (0.7%)	8 (0.4%)	8 (1.1%)	3 (7.9%)	<0.001
Systolic blood pressure, mmHg	129.7 ± 16.2	129 ± 15.5	131.9 ± 17.5	130.6 ± 20.7	<0.001
Diastolic blood pressure, mmHg	80.8 ± 11.6	81 ± 11.2	80.6 ± 12.7	75 ± 13.3	<0.001
Heart rate, beat per minute	86.8 ± 13.4	86.2 ± 12.7	88.1 ± 14.8	93.2 ± 19.1	<0.001
Respiratory rate, breaths per minute	20.4 ± 3.0	20 ± 2.4	21.2 ± 4.0	23.7 ± 6.0	<0.001
**Chest CT on admission**, n (%)					<0.001
Normal	50 (1.7%)	50 (2.3%)	0 (0.0%)	0 (0.0%)	
Interstitial pneumonia	1,389 (47.7%)	1055 (48.8%)	327 (45.8%)	7 (18.4%)	
Ground glass opacity	1,362 (46.8%)	1008 (46.7%)	343 (48.0%)	11 (28.9%)	

Local consolidation	69 (2.4%)	35 (1.6%)	26 (3.6%)	8 (21.1%)	
Bilateral consolidation	42 (1.4)	12 (0.6)	18 (2.5)	12 (31.6)	
**Laboratory examination on admission**					
White blood cell count, ×10^9/L	6.2 ± 2.8	6 ± 2.4	6.7 ± 3.6	10.2 ± 5.4	<0.001
Neutrophil count, ×10^9/L	4.0 ± 2.7	3.8 ± 2.2	4.7 ± 3.6	8.9 ± 5.4	<0.001
Lymphocyte count, ×10^9/L	0.7 ± 2.6	0.6 ± 2.4	0.8 ± 3.3	0.9 ± 2.6	0.254
Haemoglobin, g/L	124.2 ± 18.4	125.6 ± 17.8	120.3 ± 19.3	116 ± 20.5	<0.001
Platelet count, ×10^9/L	232.1 ± 82.4	235.3 ± 79.3	224.2 ± 88.4	195 ± 118.1	<0.001
Alanine aminotransferase, U/L	33 ± 34.8	32.9 ± 33.6	33.1 ± 37.9	37.2 ± 38.4	0.758
Aspartate aminotransferase, U/L	25.7 ± 41.3	25 ± 43.5	26.9 ± 33.8	42.9 ± 44.3	0.019
Albumin, g/L	37.8 ± 9.4	38.3 ± 9	36.6 ± 10.5	32.5 ± 7	<0.001
Total.bilirubin, μmol/L	10.3 ± 6.6	9.9 ± 5.8	11.1 ± 6.7	17.5 ± 21.7	<0.001
Alkaline phosphatase, U/L	75.6 ± 32.8	74.1 ± 32.1	79.4 ± 34.1	93.5 ± 40.2	<0.001
Gamma-glutamyl transpeptidase, U/L	45.3 ± 49.3	43.4 ± 46.2	50 ± 54.9	69.3 ± 85.4	<0.001
Creatinine, μmol/L	70.5 ± 48.8	68.9 ± 38.8	72.7 ± 47.2	123.9 ± 230.4	<0.001
Blood urea nitrogen, mmol/L	5.4 ± 12.1	5.2 ± 13.8	5.6 ± 3.8	9.9 ± 7.8	0.056
Potassium, mmol/L	4.4 ± 2.9	4.3 ± 1	4.5 ± 5.5	4.3 ± 0.6	0.259
Sodium, mmol/L	141.7 ± 24.3	142.1 ± 27.6	140.4 ± 9.5	142.7 ± 7.3	0.240
D-dimer, μg/mL	1.1 ± 4.4	0.8 ± 2.8	1.8 ± 7.3	4.1 ± 4	<0.001
Prothrombin time, seconds	10.8 ± 6	10.6 ± 6.3	11.4 ± 5.1	12.8 ± 6	<0.001
Activated partial thromboplastin time, seconds	28.1 ± 6.8	28.1 ± 5.7	27.8 ± 8.9	30.7 ± 13	0.027
International normalised ratio	1.2 ± 3.1	1.2 ± 2.7	1.3 ± 4.1	1.6 ± 2.5	0.407
Creatine kinase, U/L	62.1 ± 64.2	59.7 ± 51.1	64.6 ± 77.1	152.8 ± 219.6	<0.001
Lactate dehydrogenase,U/L	198.5 ± 90	185.9 ± 76.1	228.1 ± 106.7	357 ± 166.7	<0.001
Procalcitonin, ng/mL	0.2 ± 0.7	0.1 ± 0.8	0.2 ± 0.6	0.4 ± 0.6	0.045
C-reactive protein, mg/L	13.6 ± 30.4	8.9 ± 19.7	25.1 ± 44.8	67.6 ± 67.6	<0.001

Plus-minus values are means ± standard deviation.

Abbreviations: COVID-19, coronavirus disease 2019, CT, computed tomography.

Supplementary Table 2. In-hospital treatment and outcomes according to the severity of COVID-19 infection on admission

Variable	Total (n=2912)	Mild (n=2160)	Severe (n=714)	Critical (n=38)	P value
Antiviral therapy, n (%)	1,338 (45.9%)	917 (42.5%)	400 (56.0%)	21 (55.3%)	<0.001
Abidor	1,191 (40.9%)	790 (36.6%)	381 (53.4%)	20 (52.6%)	<0.001
Ribavirin	89 (3.1%)	65 (3.0%)	23 (3.2%)	1 (2.6%)	0.949
Oseltamivir	223 (7.7%)	188 (8.7%)	30 (4.2%)	5 (13.2%)	<0.001
Interferon	235 (8.1%)	139 (6.4%)	93 (13.0%)	3 (7.9%)	<0.001
Antibiotics, n (%)	964 (33.1%)	602 (27.9%)	328 (45.9%)	34 (89.5%)	<0.001
Quinolones	699 (24.0%)	445 (20.6%)	239 (33.5%)	15 (39.5%)	<0.001
Cephalosporins	87 (3.0%)	27 (1.2%)	51 (7.1%)	9 (23.7%)	<0.001
Macrolides	31 (1.1%)	23 (1.1%)	6 (0.8%)	2 (5.3%)	0.035
Traditional Chinese medicine, n (%)	2,627 (90.2%)	1971 (91.2%)	633 (88.7%)	23 (60.5%)	<0.001
Immunoglobin, n (%)	134 (4.6%)	45 (2.1%)	79 (11.1%)	10 (26.3%)	<0.001
Systemic glucocorticoid therapy, n (%)	414 (14.2%)	193 (8.9%)	197 (27.6%)	24 (63.2%)	<0.001
High-flow nasal cannula, n (%)	1,771 (60.8%)	1217 (56.3%)	520 (72.8%)	34 (89.5%)	<0.001
Continuous renal replacement therapy, n (%)	10 (0.3%)	1 (0.0%)	5 (0.7%)	4 (10.5%)	<0.001
Extracorporeal membrane oxygenation, n (%)	3 (0.1%)	2 (0.1%)	1 (0.1%)	0 (0.0%)	0.924
Mechanical ventilation, n (%)	75 (2.6%)	12 (0.6%)	41 (5.7%)	22 (57.9%)	<0.001
Non-invasive	28 (1.0%)	6 (0.3%)	17 (2.4%)	5 (13.2%)	
Invasive	12 (0.4%)	2 (0.1%)	4 (0.6%)	6 (15.8%)	
Noninvasive and Invasive	35 (1.2%)	4 (0.2%)	20 (2.8%)	11 (28.9%)	
ICU admission, n (%)	106 (3.6%)	13 (0.6%)	65 (9.1%)	28 (73.7%)	<0.001
Death, n (%)	61 (2.1%)	10 (0.5%)	31 (4.3%)	20 (52.6%)	<0.001

Plus-minus values are means ± standard deviation.

ICU, intensive care unit.

Supplementary Table 3. Baseline characteristics of patients according to with versus without de novo abnormal liver function during hospitalization (n=1498)

Variable	Total (n=1498)	Without de novo abnormal LFTs (n=1130)	With de novo abnormal LFTs (n=368)	p-value
Age (years)	56.6 ± 14.2	56.1 ± 14	58.1 ± 14.7	0.018
Female gender, n (%)	897 (59.9%)	680 (60.2%)	217 (59.0%)	0.726
Smoking history, n (%)	110 (7.3%)	84 (7.4%)	26 (7.1%)	0.904
Drinking history, n (%)	70 (4.7%)	55 (4.9%)	15 (4.1%)	0.630
**Severity of COVID-19 infection**, n (%)				<0.001
Mild	1,174 (78.4%)	902 (79.8%)	272 (73.9%)	
Severe	319 (21.3%)	227 (20.1%)	92 (25.0%)	
Critical	5 (0.3%)	1 (0.1%)	4 (1.1%)	
**Comorbidities on admission**	616 (41.1%)	457 (40.4%)	159 (43.2%)	0.382
Hypertension, n (%)	461 (30.8%)	341 (30.2%)	120 (32.6%)	0.416
Cardiovascular disease, n (%)	107 (7.1%)	75 (6.6%)	32 (8.7%)	0.224
Diabetes, n (%)	205 (13.7%)	158 (14.0%)	47 (12.8%)	0.617
Chronic pulmonary diseases, n (%)	63 (4.2%)	45 (4.0%)	18 (4.9%)	0.545
Cerebrovascular disease, n (%)	55 (3.7%)	36 (3.2%)	19 (5.2%)	0.111
Malignancy, n (%)	25 (1.7%)	19 (1.7%)	6 (1.6%)	1
Gastrointestinal diseases, n (%)	22 (1.5%)	19 (1.7%)	3 (0.8%)	0.342
Autoimmune disease, n (%)	7 (0.5%)	5 (0.4%)	2 (0.5%)	1
**Chronic liver diseases, n (%)**	31 (2.1%)	25 (2.2%)	6 (1.6%)	0.638
Hepatitis B virus infection, n (%)	26 (1.7%)	20 (1.8%)	6 (1.6%)	1
Hepatitis C virus infection, n (%)	5 (0.3%)	5 (0.4%)	0 (0.0%)	0.449
Autoimmune liver disease, n (%)	0 (0.0%)	0 (0.0%)	0 (0.0%)	NA
**Clinical characteristics on admission**				
Fever (>37.3 C), n (%)	1,029 (68.7%)	778 (68.8%)	251 (68.2%)	0.868
Cough, n (%)	998 (66.6%)	752 (66.5%)	246 (66.8%)	0.966
Expectoration, n (%)	186 (12.4%)	148 (13.1%)	38 (10.3%)	0.190
Dyspnea, n (%)	651 (43.5%)	472 (41.8%)	179 (48.6%)	0.025
Fatigue, n (%)	697 (46.5%)	515 (45.6%)	182 (49.5%)	0.216
Myalgia, n (%)	357 (23.8%)	259 (22.9%)	98 (26.6%)	0.167
Anorexia, n (%)	228 (15.2%)	169 (15.0%)	59 (16.0%)	0.677
Nausea, n (%)	38 (2.5%)	29 (2.6%)	9 (2.4%)	1
Vomiting, n (%)	31 (2.1%)	24 (2.1%)	7 (1.9%)	0.961
Abdominal pain, n (%)	17 (1.1%)	14 (1.2%)	3 (0.8%)	0.702
Diarrhea, n (%)	56 (3.7%)	45 (4.0%)	11 (3.0%)	0.475
Headache, n (%)	30 (2.0%)	21 (1.9%)	9 (2.4%)	0.628
Dizziness, n (%)	24 (1.6%)	21 (1.9%)	3 (0.8%)	0.252
Disorders of consciousness, n (%)	5 (0.3%)	2 (0.2%)	3 (0.8%)	0.186
Systolic blood pressure (mmHg)	130.3 ± 16.6	130.8 ± 17	128.7 ± 15.1	0.039
Diastolic blood pressure (mmHg)	81.5 ± 11.3	81.7 ± 11.6	80.7 ± 10.4	0.116
Heart rate (beat per minute)	87.2 ± 13.3	87.3 ± 13.4	86.8 ± 13.2	0.499

Respiratory rate (breaths per minute)	20.1 ± 2.7	20.2 ± 2.9	20 ± 1.9	0.332
**Chest CT on admission**, n (%)				
Normal	33 (2.2)	29 (2.6)	4 (1.1)	
Interstitial pneumonia	707 (47.2)	520 (46.0)	187 (50.8)	
Ground glass opacity	719 (48.0)	554 (49.0)	165 (44.8)	
Local consolidation	29 (1.9)	20 (1.8)	9 (2.4)	
Bilateral consolidation	10 (0.7)	7 (0.6)	3 (0.8)	0.204
**Laboratory examination on admission**				
White blood cell count ($\times 10^9$/L)	5.9 ± 2.1	5.8 ± 2.1	6 ± 2.1	0.105
Neutrophil count ($\times 10^9$/L)	3.6 ± 1.6	3.5 ± 1.5	3.8 ± 1.9	<0.001
Lymphocyte count ($\times 10^9$/L)	0.6 ± 1.2	0.6 ± 0.9	0.8 ± 1.9	<0.001
Hemoglobin (g/L)	124.9 ± 16.2	125.2 ± 16.6	124.2 ± 14.7	0.316
Platelet count ($\times 10^9$/L)	225.3 ± 69.9	226.2 ± 70.7	222.4 ± 67.7	0.370
Alanine aminotransferase, U/L	19.6 ± 8.4	19.6 ± 8.3	19.4 ± 8.6	0.730
Aspartate aminotransferase, U/L	18.2 ± 5.2	18.2 ± 5	18.5 ± 5.6	0.262
Albumin, g/L	39.7 ± 9.4	39.6 ± 3.5	40 ± 17.9	0.549
Total.bilirubin, μmol/L	9.6 ± 4.2	9.6 ± 4.2	9.6 ± 4.3	0.939
Alkaline phosphatase, U/L	68.8 ± 17.6	68.5 ± 17.6	70 ± 17.7	0.139
Gamma-glutamyl transpeptidase, U/L	26.9 ± 12.3	26.6 ± 11.7	27.7 ± 13.9	0.142
Creatinine, μmol/L	67.4 ± 48.3	66.3 ± 34.2	70.9 ± 76.7	0.113
Blood urea nitrogen, mmol/L	5.2 ± 15.2	5.3 ± 17.4	5 ± 3.4	0.726
Potassium, mmol/L	4.3 ± 1.5	4.3 ± 0.4	4.5 ± 2.9	0.011
Sodium, mmol/L	141.7 ± 4.3	141.7 ± 4.7	141.6 ± 2.7	0.608
D-dimer, μg/mL	0.7 ± 2.5	0.7 ± 2.8	0.7 ± 1	0.926
Prothrombin time, seconds	10.8 ± 5.3	11 ± 5.3	10.5 ± 5.1	0.125
Activated partial thromboplastin time, seconds	27.8 ± 3.9	27.8 ± 3.9	27.6 ± 3.9	0.421
International normalised ratio	1.2 ± 3.8	1.3 ± 4.4	1.1 ± 0.2	0.432
Creatine kinase, U/L	59.1 ± 42.8	58.7 ± 35.1	60.2 ± 60.6	0.549
Lactate dehydrogenase,U/L	175.8 ± 64.2	173.7 ± 66.2	182.3 ± 57.6	0.026
Procalcitonin, ng/mL	0.1 ± 0.7	0.1 ± 0.8	0.1 ± 0.3	0.728
C-reactive protein, mg/L	5.5 ± 15.8	4.4 ± 10.8	8.9 ± 25.2	<0.001
**Liver function tests during hospitalization**				
Peak aspartate aminotransferase (ALT), U/L	31.6 ± 56.7	19.9 ± 8.5	67.5 ± 105.7	<0.001
ALT <40 U/L, n (%)	1,303 (87.0%)	1,130 (100.0%)	173 (47.0%)	
ALT 40-120 U/L, n (%)	156 (10.4%)	0 (0.0%)	156 (42.4%)	
ALT >120 U/L, n (%)	39 (2.6%)	0 (0.0%)	39 (10.6%)	
Peak aspartate aminotransferase (AST), U/L	27.2 ± 51.6	18.3 ± 5.2	54.3 ± 98.9	<0.001
AST <45 U/L, n (%)	1,409 (94.1%)	1,130 (100.0%)	279 (75.8%)	
AST 45-135 U/L, n (%)	67 (4.5%)	0 (0.0%)	67 (18.2%)	
AST >135 U/L, n (%)	22 (1.5%)	0 (0.0%)	22 (6.0%)	
Nadir albumin, g/L	37.6 (4.8	39.2 (3.4	32.7 (5.3	<0.001
Albumin >40 g/L, n (%)	391 (26.1%)	374 (33.1%)	17 (4.6%)	
Albumin 30-40 g/L, n (%)	1,015 (67.8%)	756 (66.9%)	259 (70.4%)	
Albumin <30 g/L, n (%)	92 (6.1%)	0 (0.0%)	92 (25.0%)	

Peak total bilirubin (TBIL), μmol/L	10.9 ± 8	9.8 ± 3.8	14.5 ± 14.2	<0.001
TBIL > 26 μmol/L, n (%)	34 (2.3%)	0 (0.0%)	34 (9.2%)	
Peak alkaline phosphatase (ALP), U/L	73.1 ± 28.8	68.7 ± 17.5	86.5 ± 47	<0.001
ALP >125 U/L, n (%)	34 (2.3%)	0 (0.0%)	34 (9.2%)	
Peak γ-glutamyl transpeptidase (GGT), U/L	32.9 ± 30.9	26.6 ± 11.6	52.2 ± 54.6	<0.001
GGT <60 U/L, n (%)	1,396 (93.2%)	1,130 (100.0%)	266 (72.3%)	
GGT 60-180 U/L, n (%)	92 (6.1%)	0 (0.0%)	92 (25.0%)	
GGT >180 U/L, n (%)	10 (0.7%)	0 (0.0%)	10 (2.7%)	

Plus-minus values are means ± standard deviation.

Abbreviations: COVID-19, coronavirus disease 2019, CT, computed tomography.

Supplementary Table 4. In-hospital treatment and outcomes according to with versus without de novo abnormal liver function during hospitalization

Variable	Total (n=1498)	Without de novo abnormal LFTs (n=1130)	With de novo abnormal LFTs (n=368)	P value
Antiviral therapy, n (%)	594 (39.7%)	443 (39.2%)	151 (41.0%)	0.574
Include abidor, n (%)	556 (37.1%)	414 (36.6%)	142 (38.6%)	0.542
Include ribavirin, n (%)	22 (1.5%)	16 (1.4%)	6 (1.6%)	0.962
Include oseltamivir, n (%)	63 (4.2%)	47 (4.2%)	16 (4.3%)	0.994
Include interferon, n (%)	113 (7.5%)	94 (8.3%)	19 (5.2%)	0.06
Antibiotics, n (%)	372 (24.8%)	262 (23.2%)	110 (29.9%)	0.012
Quinolones, n (%)	265 (17.7%)	180 (15.9%)	85 (23.1%)	<0.001
Cephalosporins, n (%)	14 (0.9%)	5 (0.4%)	9 (2.4%)	<0.001
Macrolides, n (%)	12 (0.8%)	10 (0.9%)	2 (0.5%)	0.763
Traditional Chinese medicine, n (%)	1,362 (90.9%)	1024 (90.6%)	338 (91.8%)	0.543
Immunoglobin, n (%)	35 (2.3%)	25 (2.2%)	10 (2.7%)	0.72
Glucocorticoid therapy, n (%)	116 (7.7%)	78 (6.9%)	38 (10.3%)	0.043
High flow nasal cannula, n (%)	848 (56.6%)	636 (56.3%)	212 (57.6%)	0.7
Continuous renal replacement therapy, n (%)	0 (0.0%)	0 (0.0%)	0 (0.0%)	NA
Extracorporeal membrane oxygenation, n (%)	0 (0.0%)	0 (0.0%)	0 (0.0%)	NA
Mechanical ventilation, n (%)	13 (0.9%)	6 (0.5%)	7 (1.9%)	0.032
Non-invasive	7 (0.5%)	4 (0.4%)	3 (0.8%)	
Invasive	3 (0.2%)	1 (0.1%)	2 (0.5%)	
Noninvasive + Invasive	3 (0.2%)	1 (0.1%)	2 (0.5%)	0.07
Admission or transfer to ICU, n (%)	18 (1.2%)	8 (0.7%)	10 (2.7%)	<0.001
Death, n (%)	7 (0.5%)	2 (0.2%)	5 (1.4%)	0.014
Length of hospital stay (days)	12.8 ± 7.4	12.5 ± 7.4	14 ± 7.4	<0.001
Composite endpoint †	22 (1.5%)	10 (0.9%)	12 (3.3%)	<0.001

Plus-minus values are means ± standard deviation.

† The composite end-points consist of admission to intensive care unit, mechanical ventilation or death

ICU, intensive care unit.

Supplementary Table 5. Baseline characteristics of patients according to normal versus abnormal liver function during hospitalization in entire cohort (n=2912)

**Variable**	**Total (n=2912)**	**Normal (n=1130)**	**Abnormal (n=1782)**	**P value**
Age (years)	58.4 ± 14.4	56.1 ± 14	59.8 ± 14.5	<0.001
Female gender, n (%)	1,512 (51.9%)	680 (60.2%)	832 (46.7%)	<0.001
Smoking history, n (%)	217 (7.5%)	84 (7.4%)	133 (7.5%)	1.000
Drinking history, n (%)	130 (4.5%)	55 (4.9%)	75 (4.2%)	0.455
**Severity of COVID19**, n (%)				
Mild	2,160 (74.2%)	902 (79.8%)	1,258 (70.6%)	
Severe	714 (24.5%)	227 (20.1%)	487 (27.3%)	
Critical	38 (1.3%)	1 (0.1%)	37 (2.1%)	<0.001
**Comorbidities on admission**	1,236 (42.4%)	457 (40.4%)	779 (43.7%)	0.089
Hypertension, n (%)	910 (31.2%)	341 (30.2%)	569 (31.9%)	0.340
Cardiovascular disease, n (%)	219 (7.5%)	75 (6.6%)	144 (8.1%)	0.172
Diabetes, n (%)	392 (13.5%)	158 (14.0%)	234 (13.1%)	0.549
Chronic pulmonary diseases, n (%)	141 (4.8%)	45 (4.0%)	96 (5.4%)	0.103
Cerebrovascular disease, n (%)	125 (4.3%)	36 (3.2%)	89 (5.0%)	0.024
Malignancy, n (%)	63 (2.2%)	19 (1.7%)	44 (2.5%)	0.196
Gastrointestinal diseases, n (%)	53 (1.8%)	19 (1.7%)	34 (1.9%)	0.762
Autoimmune disease, n (%)	20 (0.7%)	5 (0.4%)	15 (0.8%)	0.298
**Chronic liver diseases, n (%)**	68 (2.3%)	25 (2.2%)	43 (2.4%)	0.823
Hepatitis B virus infection, n (%)	58 (2.0%)	20 (1.8%)	38 (2.1%)	0.585
Hepatitis C virus infection, n (%)	8 (0.3%)	5 (0.4%)	3 (0.2%)	0.311
Autoimmune liver disease, n (%)	2 (0.1%)	0 (0.0%)	2 (0.1%)	0.689
**Clinical characteristics on admission**				
Fever (>38 ^{o}C), n (%)	2,057 (70.6%)	778 (68.8%)	1279 (71.8%)	0.100
Cough, n (%)	2,001 (68.7%)	752 (66.5%)	1249 (70.1%)	0.049
Expectoration, n (%)	420 (14.4%)	148 (13.1%)	272 (15.3%)	0.117
Dyspnea, n (%)	1,394 (47.9%)	472 (41.8%)	922 (51.7%)	<0.001
Fatigue, n (%)	1,461 (50.2%)	515 (45.6%)	946 (53.1%)	<0.001
Myalgia, n (%)	774 (26.6%)	259 (22.9%)	515 (28.9%)	<0.001
Anorexia, n (%)	523 (18.0%)	169 (15.0%)	354 (19.9%)	<0.001
Nausea, n (%)	63 (2.2%)	29 (2.6%)	34 (1.9%)	0.289
Vomiting, n (%)	47 (1.6%)	24 (2.1%)	23 (1.3%)	0.112
Abdominal pain, n (%)	31 (1.1%)	14 (1.2%)	17 (1.0%)	0.586
Diarrhea, n (%)	126 (4.3%)	45 (4.0%)	81 (4.5%)	0.526
Headache, n (%)	54 (1.9%)	21 (1.9%)	33 (1.9%)	1.000
Dizziness, n (%)	36 (1.2%)	21 (1.9%)	15 (0.8%)	0.025
Disorders of consciousness, n (%)	19 (0.7%)	2 (0.2%)	17 (1.0%)	0.021
Systolic blood pressure (mmHg)	129.7 ± 16.2	130.8 ± 17	129.1 ± 15.5	<0.001
Diastolic blood pressure (mmHg)	80.8 ± 11.6	81.7 ± 11.6	80.3 ± 11.6	<0.001
Heart rate (beat per minute)	86.8 ± 13.4	87.3 ± 13.4	86.4 ± 13.4	0.083
Respiratory rate (breaths per minute)	20.4 ± 3.0	20.2 ± 2.9	20.5 ± 3.1	0.013

**Chest CT on admission**, n (%)				
Normal	50 (1.7%)	29 (2.6%)	21 (1.2%)	
Interstitial pneumonia	1,389 (47.7%)	520 (46.0%)	869 (48.8%)	
Ground glass opacity	1,362 (46.8%)	554 (49.0%)	808 (45.3%)	
Local consolidation	69 (2.4%)	20 (1.8%)	49 (2.7%)	
Bilateral consolidation	42 (1.4%)	7 (0.6%)	35 (2.0%)	<0.001
**Laboratory examination on admission**				
White blood cell count (×10^9/L)	6.2 ± 2.8	5.8 ± 2.1	6.4 ± 3.2	<0.001
Neutrophil count (×10^9/L)	4.0 ± 2.7	3.5 ± 1.5	4.4 ± 3.2	<0.001
Lymphocyte count (×10^9/L)	0.7 ± 2.6	0.6 ± 0.9	0.8 ± 3.3	0.019
Hemoglobin (g/L)	124.2 ± 18.4	125.2 ± 16.6	123.6 ± 19.4	0.022
Platelet count (×10^9/L)	232.1 ± 82.4	226.2 ± 70.7	235.8 ± 88.9	<0.001
Alanine aminotransferase (ALT), U/L	33 ± 34.8	19.6 ± 8.3	41.5 ± 41.8	<0.001
ALT <40 U/L, n (%)	2,250 (77.3%)	1,130 (100.0%)	1,120 (62.9%)	
ALT 40-120 U/L, n (%)	584 (20.1%)	0 (0.0%)	584 (32.8%)	
ALT >120 U/L, n (%)	78 (2.7%)	0 (0.0%)	78 (4.4%)	
Aspartate aminotransferase (AST), U/L	25.7 ± 41.3	18.2 ± 5	30.5 ± 52.1	<0.001
AST <45 U/L, n (%)	2,691 (92.4%)	1,130 (100.0%)	1,561 (87.6%)	
AST 45-135 U/L, n (%)	200 (6.9%)	0 (0.0%)	200 (11.2%)	
AST >135 U/L, n (%)	21 (0.7%)	0 (0.0%)	21 (1.2%)	
Albumin (g/L)	37.8 ± 9.4	39.6 ± 3.5	36.7 ± 11.6	<0.001
Albumin >40 g/L, n (%)	826 (28.4%)	437 (38.7%)	389 (21.8%)	
Albumin 30-40 g/L, n (%)	1,925 (66.1%)	693 (61.3%)	1232 (69.1%)	
Albumin <30 g/L, n (%)	161 (5.5%)	0 (0.0%)	161 (9.0%)	
Total bilirubin (TBIL), μmol/L	10.3 ± 6.6	9.6 ± 4.2	10.7 ± 7.7	<0.001
TBIL >26μmol/L	52 (1.8%)	0 (0.0%)	52 (2.9%)	<0.001
Alkaline phosphatase (ALP), U/L	75.6 ± 32.8	68.5 ± 17.6	80.2 ± 38.9	<0.001
ALP >125 U/L, n (%)	135 (4.6%)	0 (0.0%)	135 (7.6%)	<0.001
Gamma-glutamyl transpeptidase (GGT), U/L	45.3 ± 49.3	26.6 ± 11.7	57.2 ± 59.3	<0.001
GGT <60 U/L, n (%)	2,373 (81.5%)	1,130 (100.0%)	1,243 (69.8%)	
GGT 60-180 U/L, n (%)	459 (15.8%)	0 (0.0%)	459 (25.8%)	
GGT >180 U/L, n (%)	80 (2.7%)	0 (0.0%)	80 (4.5%)	
Creatinine (μmol/L)	70.5 ± 48.8	66.3 ± 34.2	73.2 ± 55.9	<0.001
Blood urea nitrogen (mmol/L)	5.4 ± 12.1	5.3 ± 17.4	5.4 ± 6.7	0.806
Potassium (mmol/L)	4.4 ± 2.9	4.3 ± 0.4	4.4 ± 3.6	0.108
Sodium (mmol/L)	141.7 ± 24.3	141.7 ± 4.7	141.7 ± 30.8	0.991
D-dimer (μg/mL)	1.1 ± 4.4	0.7 ± 2.8	1.3 ± 5.1	<0.001
Prothrombin time (seconds)	10.8 ± 6	11 ± 5.3	10.7 ± 6.4	0.288
Activated partial thromboplastin time (seconds)	28.1 ± 6.8	27.8 ± 3.9	28.3 ± 8.1	0.089
International normalised ratio	1.2 ± 3.1	1.3 ± 4.4	1.2 ± 1.9	0.440
Creatine kinase (U/L)	62.1 ± 64.2	58.7 ± 35.1	64.3 ± 77.1	0.023
Lactate dehydrogenase (U/L)	198.5 ± 90	173.7 ± 66.2	214.2 ± 99.1	<0.001
Procalcitonin (ng/mL)	0.2 ± 0.7	0.1 ± 0.8	0.2 ± 0.7	<0.001
C-reactive protein, mg/L)	13.6 ± 30.4	4.4 ± 10.8	19.5 ± 36.7	<0.001

**Liver function tests during hospitalization**				
Peak aspartate aminotransferase (ALT), U/L	41.4 ± 60.3	19.9 ± 8.5	55 ± 73.6	<0.001
ALT <40 U/L, n (%)	2,057 (70.6%)	1,130 (100.0%)	927 (52.0%)	
ALT 40-120 U/L, n (%)	721 (24.8%)	0 (0.0%)	721 (40.5%)	
ALT >120 U/L, n (%)	134 (4.6%)	0 (0.0%)	134 (7.5%)	<0.001
Peak aspartate aminotransferase (AST), U/L	31.4 ± 50.1	18.3 ± 5.2	39.6 ± 62.5	<0.001
AST <45 U/L, n (%)	2,591 (89.0%)	1,130 (100.0%)	1,461 (82.0%)	
AST 45-135 U/L, n (%)	270 (9.3%)	0 (0.0%)	270 (15.2%)	
AST >135 U/L, n (%)	51 (1.8%)	0 (0.0%)	51 (2.9%)	<0.001
Nadir albumin, g/L	35.9 ± 5.5	39.2 ± 3.4	33.8 ± 5.5	<0.001
Albumin >40 g/L, n (%)	567 (19.5%)	374 (33.1%)	193 (10.8%)	
Albumin 30-40 g/L, n (%)	2,000 (68.7%)	756 (66.9%)	1244 (69.8%)	
Albumin <30 g/L, n (%)	345 (11.8%)	0 (0.0%)	345 (19.4%)	<0.001
Peak total bilirubin (TBIL), μmol/L	11.8 ± 16.8	9.8 ± 3.8	13.1 ± 21.1	<0.001
TBIL > 26 μmol/L, n (%)	103 (3.5%)	0 (0.0%)	103 (5.8%)	<0.001
Peak alkaline phosphatase (ALP), U/L	79.4 ± 42.8	68.7 ± 17.5	86.2 ± 51.8	<0.001
ALP >125 U/L, n (%)	166 (5.7%)	0 (0.0%)	166 (9.3%)	<0.001
Peak γ-glutamyl transpeptidase (GGT), U/L	49.1 ± 54.3	26.6 ± 11.6	63.4 ± 64.8	<0.001
GGT <60 U/L, n (%)	2,270 (78.0%)	1,130 (100.0%)	1,140 (64.0%)	
GGT 60-180 U/L, n (%)	560 (19.2%)	0 (0.0%)	560 (31.4%)	
GGT >180 U/L, n (%)	82 (2.8%)	0 (0.0%)	82 (4.6%)	<0.001

Plus-minus values are means ± standard deviation.

Abbreviations: COVID19, coronavirus disease 2019.

Supplementary Table 6. In-hospital treatment and outcomes according to normal versus abnormal liver function during hospitalization

Variable	Total (n=2912)	Normal (n=1130)	Abnormal (n=1782)	P value
Antiviral therapy, n (%)	1,338 (45.9%)	443 (39.2%)	895 (50.2%)	<0.001
Include abidor, n (%)	1,191 (40.9%)	414 (36.6%)	777 (43.6%)	<0.001
Include ribavirin, n (%)	89 (3.1%)	16 (1.4%)	73 (4.1%)	<0.001
Include oseltamivir, n (%)	223 (7.7%)	47 (4.2%)	176 (9.9%)	<0.001
Include interferon, n (%)	235 (8.1%)	94 (8.3%)	141 (7.9%)	0.747
Antibiotics, n (%)	964 (33.1%)	262 (23.2%)	702 (39.4%)	<0.001
Quinolones, n (%)	699 (24.0%)	180 (15.9%)	519 (29.1%)	<0.001
Cephalosporins, n (%)	87 (3.0%)	5 (0.4%)	82 (4.6%)	<0.001
Macrolides, n (%)	31 (1.1%)	10 (0.9%)	21 (1.2%)	0.571
Traditional Chinese medicine, n (%)	2,627 (90.2%)	1024 (90.6%)	1603 (90.0%)	0.6
Immunoglobin, n (%)	134 (4.6%)	25 (2.2%)	109 (6.1%)	<0.001
Glucocorticoid therapy, n (%)	414 (14.2%)	78 (6.9%)	336 (18.9%)	<0.001
High flow nasal cannula, n (%)	1,771 (60.8%)	636 (56.3%)	1135 (63.7%)	<0.001
Continuous renal replacement therapy, n (%)	10 (0.3%)	0 (0.0%)	10 (0.6%)	0.028
Extracorporeal membrane oxygenation, n (%)	3 (0.1%)	0 (0.0%)	3 (0.2%)	0.431
Mechanical ventilation, n (%)	75 (2.6%)	6 (0.5%)	69 (3.9%)	<0.001
Non-invasive	28 (1.0%)	4 (0.4%)	24 (1.3%)	
Invasive	12 (0.4%)	1 (0.1%)	11 (0.6%)	
Noninvasive + Invasive	35 (1.2%)	1 (0.1%)	34 (1.9%)	
ICU admission, n (%)	106 (3.6%)	8 (0.7%)	98 (5.5%)	<0.001
Death, n (%)	61 (2.1%)	2 (0.2%)	59 (3.3%)	<0.001
length of hospital stay (days)	14.9 ± 9.0	12.5 ± 7.4	16.5 ± 9.6	<0.001
Composite endpoint	121 (4.2%)	10 (0.9%)	111 (6.2%)	<0.001

Plus-minus values are means ± standard deviation.

† The composite end-points consist of admission to intensive care unit, mechanical ventilation or death

ICU, intensive care unit.

Supplementary Table 7. Associations between peak (nadir) liver function tests and clinical outcomes

Liver function test	Event rate, n/N (%)	Unadjusted HR (95% CI)	P value	Adjusted HR (95% CI)	P value
**Death**					
**Peak ALT (U/L)**					
<40	39/2057 (1.9%)	Ref		Ref	
40-120	18/721 (2.5%)	1.06 (0.60 - 1.86)	0.844	1.05 (0.59 - 1.88)	0.865
>120	4/134 (3.0%)	1.35 (0.48 - 3.79)	0.567	1.20 (0.42 - 3.42)	0.737
**Nadir AST (U/L)**					
<45	38/2519 (1.5%)	Ref		Ref	
45-135	20/270 (7.4%)	4.30 (2.49 - 7.43)	<0.001	3.46 (1.97 - 6.07)	< 0.001
>135	3/51 (5.9%)	4.60 (1.42 - 14.93)	0.011	6.15 (1.88 - 20.13)	0.0027
**Nadir albumin (g/L)**					
>40	0/567 (0%)	NE	NE	NE	NE
30-40	30/2000 (1.5%)	NE	NE	NE	NE
<30	31/345 (9.0%)	NE	NE	NE	NE
**Peak TBIL (μmol/L)**					
≤26	52/2809 (1.9%)	Ref		Ref	
>26	9/103 (8.7%)	4.52 (2.20 - 9.28)	<0.001	2.73 (1.29 - 5.78)	0.00857
**Peak ALP (U/L)**					
≤125	50/2746 (1.8%)	Ref		Ref	
>125	11/166 (6.6%)	3.34 (1.73 - 6.44)	<0.001	2.94 (1.52 - 5.68)	0.00134
**Peak GGT (U/L)**					
<60	41/2270 (1.8%)	Ref		Ref	
60-80	17/560 (3.0%)	1.35 (0.77 - 2.39)	0.296	1.11 (0.62 - 1.99)	0.728
>180	3/82 (3.7%)	1.40 (0.43 - 4.59)	0.576	1.93 (0.58 - 6.41)	0.281
**Transfer to ICU**					
**Peak ALT (U/L)**					
<40	74/2591 (2.9%)	Ref		Ref	
40-120	29/270 (10.7%)	1.50 (0.99 - 2.28)	0.053	1.62 (1.07 - 2.48)	0.024
>120	3/51 (5.9%)	1.189 (0.48 - 2.95)	0.710	1.32 (0.52 - 3.35)	0.550
**Peak AST (U/L)**					
<45	74/2591 (2.9%)	Ref		Ref	
45-135	29/270 (10.7%)	4.0279 (2.62 - 6.19)	<0.001	3.52 (2.28 - 5.44)	<0.001
>135	3/51 (5.9%)	2.1438 (0.68 - 6.76)	0.190	2.95 (1.11 - 7.83)	0.003
**Nadir albumin (g/L)**					
>40	0/567 (0%)	NE	NE	NE	NE
30-40	55/2000 (2.8%)	NE	NE	NE	NE
<30	51/345 (14.8%)	NE	NE	NE	NE
**Peak TBIL (μmol/L)**					
≤26	96/2809 (3.4%)	Ref		Ref	
>26	10/103 (9.7%)	3.01 (1.57 - 5.79)	<0.001	2.30 (1.22 - 4.33)	<0.001
**Peak ALP (U/L)**					
≤125	88/2746 (3.2%)	Ref		Ref	

>125	18/166 (10.8%)	3.35 (2.00 - 5.62)	<0.001	2.4675 (1.43 - 4.26)	<0.001
**Peak GGT (U/L)**					
<60	70/2270 (3.1%)	Ref		Ref	
60-80	30/560 (5.4%)	1.72 1.12 - 2.66)	0.014	1.4733 (0.95 - 2.29)	0.086
>180	6/82 (7.3%)	2.46 (1.07 - 5.67)	0.034	2.9876 (1.26 - 7.07)	0.013
**Mechanical ventilation**					
**Peak ALT (U/L)**					
<40	46/2057 (2.2%)	Ref		Ref	
40-120	25/721 (3.5%)	1.55 (0.96 – 2.52)	0.076	1.72 (1.06 - 2.80)	0.029
>120	4/134 (3.0%)	1.34 (0.48 – 3.73)	0.570	1.63 (0.60 - 4.47)	0.340
**Peak AST (U/L)**					
<45	54/2591 (2.1%)	Ref		Ref	
45-135	18/270 (6.7%)	3.28 (1.93 – 5.59)	<0.001	2.86(1.67 - 4.87)	<0.001
>135	3/51 (5.9%)	2.91 (0.90 – 9.40)	<0.001	4.59 (1.53 - 13.72)	<0.001
**Nadir albumin (g/L)**					
>40	0/567 (0%)	NE	NE	NE	NE
30-40	41/2000 (2.1%)	NE	NE	NE	NE
<30	34/345 (9.9%)	NE	NE	NE	NE
**Peak TBIL (μmol/L)**					
≤26	66/2809 (2.3%)	Ref		Ref	
>26	9/103 (8.7%)	3.83 (1.91-7.65)	<0.001	3.02 (1.56 - 5.81)	<0.001
**Peak ALP (U/L)**					
≤125	63/2746 (2.3%)	Ref		Ref	
>125	12/166 (7.2%)	3.24 (1.75 – 6.00)	<0.001	2.52 (1.34 - 4.75)	<0.001
**Peak GGT (U/L)**					
<60	49/2270 (2.2%)	Ref		Ref	
60-80	22/560 (3.9%)	1.83 (1.110 – 3.03)	0.018	1.68 (1.01 - 2.79)	0.045
>180	4/82 (4.9%)	2.29 (0.83 – 6.32)	0.110	2.82 (1.00 - 8.00)	0.051

Abbreviations: ALT, alanine aminotransferase; ALP, alkaline phosphatase; AST, aspartate aminotransferase; CI, confidence interval; GGT, gamma-glutamyltransferase; NE, not evaluateble; HR, hazard ratio; TBIL, total bilirubin.
